# Assessing the role of iodination degree on biodegradation kinetics and transformation pathways of iodinated contrast media and derivatives

**DOI:** 10.1007/s10532-025-10213-6

**Published:** 2025-11-23

**Authors:** Yuki Bartels, Martin Jekel, Anke Putschew

**Affiliations:** Department of Water Quality Control, Institute of Environmental Technology, Faculty III – Process Sciences, Technische Universität Berlin, Strasse des 17. Juni 135, 10623 Berlin, Germany

**Keywords:** Iodinated contrast media, Reductive deiodination, Biodegradation kinetics, Transformation pathways, Bank filtration, Zahn-Wellens test

## Abstract

Triiodinated aromatic compounds used as iodinated contrast media in medical imaging are poorly biodegradable. Reductive dehalogenation enhances biotransformation, but the specific influence of iodination degree on biodegradation remains unclear. This study investigated the biodegradation of twelve model substances: iopromide and diatrizoate (both iodinated contrast media), 5-amino-2,4,6-triiodoisophthalic acid (ATIA, a precursor and transformation product), and their diiodinated, monoiodinated, and deiodinated derivatives. Biodegradation kinetics were assessed in Zahn-Wellens tests and nitrate-reducing aquifer material–water batch tests. DT_50_ values were calculated to compare degradation rates. Transformation pathways were reconstructed based on literature data and detected transformation products, and evaluated for dependencies on iodination degree. In Zahn-Wellens tests, the iopromide derivatives degraded rapidly (DT_50_: 0.7–1.2 d) regardless of iodination degree. In contrast, degradation of the diatrizoate derivatives (DT_50_: 0.9–65 d) and the ATIA derivatives (DT_50_: 0.3–44 d) was strongly influenced by iodine number and position. For iopromide, transformation pathways were consistent across derivatives and included several novel transformation products beyond the previously assumed final product DDPI. In nitrate-reducing aquifer suspensions, aerobic pathways also occurred for the iopromide derivatives (DT_50_: 38.7–42.3 d). In contrast, only the monoiodinated and deiodinated diatrizoate were transformed (DT_50_: 5.6–8.2 d). Mineralization of the ATIA derivatives, measured via dissolved organic carbon, was significantly enhanced for the monoiodinated and deiodinated compound. The findings underline the importance of iodination degree for biotransformation and mineralization. This is particularly relevant for bank filtration, where (partial) deiodination to iodinated aromatics occurs before these compounds enter aerobic drinking water treatment.

**Supplementary Information:**

The online version contains supplementary material available at 10.1007/s10532-025-10213-6.

## Introduction

Iodinated X-ray contrast media (ICM) are used to enhance the contrast of organs and vessels during X-ray examinations (Christiansen 2005). ICM as well as their aerobic transformation products (TPs) are highly polar triiodobenzoic acid derivatives, ubiquitously found in the urban water cycle (Putschew et al. 2000; Ternes and Hirsch 2000; Drewes et al. 2001; Pérez and Barceló 2007; Kormos et al. 2011).

The biodegradability of the two ICM, iopromide (IOP) and diatrizoate (DTZ), has been extensively studied. Under aerobic conditions, their transformation has been observed in biodegradation tests using activated sludge (Kalsch 1999; Steger-Hartmann et al. 2002; Batt et al. 2006; Haiss and Kümmerer 2006; Pérez et al. 2006), sediment, and soil (Kalsch 1999; Ternes and Hirsch 2000; Schittko et al. 2004; Schulz et al. 2008; Kormos et al. 2010, 2011; Redeker et al. 2014, 2018). These studies consistently reported that transformation was limited to the aliphatic side chains attached to the aromatic ring, whereas the triiodinated aromatic structure remained stable.

Schulz et al. (2008) proposed a detailed aerobic transformation pathway for IOP, identifying didespropanediol iopromide (DDPI) as the final stable TP. DDPI has also been detected in bank filtrate and groundwater influenced by wastewater. For DTZ, an aerobic transformation pathway resulting in the formation of the stable 3,5-diamino-2,4,6-triiodobenzoic acid was suggested by Kalsch (1999) and later confirmed by Haiss and Kümmerer (2006).

Anaerobic transformation of IOP and DTZ was studied using sediments from sulfate-reducing environments (Redeker et al. 2014, 2018) and river sediment under anoxic conditions (Kalsch 1999). Redeker et al. (2018) observed complete deiodination and hydrolysis of amide moieties of IOP, DDPI and its primary aniline desmethoxyacetyl iopromide (DAMI), but no mineralization was detected. Similarly, Redeker et al (2014) reported complete deiodination of DTZ under anaerobic conditions, with 3,5-diaminobenzoic acid (DABA) as the final TP. Subsequent aerobic treatment led to further degradation, highlighting the potential of sequential anaerobic–aerobic treatment, such as during bank filtration followed by drinking water treatment, to enhance the removal of iodinated compounds. Lecouturier et al. (2008) demonstrated successful mineralization of the triiodinated aromatic compound 5-amino-2,4,6-triiodoisophthalic acid (ATIA), an ICM precursor, in a two-stage bioreactor system combining anaerobic deiodination with subsequent aerobic degradation.

Microbial degradation of halogenated aromatic compounds typically proceeds via a three-stage pathway (Pimviriyakul et al. 2020). First, functional groups such as acetyl or phosphate moieties are removed simplifying the structure and generating intermediates such as halogenated phenols or benzoates. The second step involves dehalogenation, a crucial reaction given that the resistance of these compounds to microbial degradation strongly depends on the type and number of halogen substituents (Fetzner and Lingens 1994). Under anaerobic conditions, reductive dehalogenation is the primary mechanism (Häggblom 1992), although it requires long adaptation phases, reflecting its metabolic complexity. In the last step, the aromatic ring is cleaved, forming aliphatic TPs.

The presence of halogen substituents (Cl, F, Br) is known to inhibit biodegradation by reducing the activity of enzymes such as dioxygenases, which are responsible for aromatic ring cleavage (Dorn and Knackmuss 1978; Andreozzi et al. 2006). Two mechanisms are proposed for this inhibition: strong electron-withdrawing effects reduce the ring’s nucleophilicity hindering electrophilic attack by the dioxygenase, while the large atomic radius of halogens causes steric hindrance. These effects vary with the enzyme type and the position of halogen atoms (Dorn and Knackmuss 1978; Häggblom 1992; Cvetnic et al. 2017). While the influence of Cl, F and Br on the aerobic biodegradation of aromatic compounds have been already studied, there is a lack of investigation for iodinated aromatics.

Deiodination of ICM and ICM TPs occurs in anoxic or anaerobic zones in the subsurface of bank filtration sites, but it was shown, that the triiodinated compounds were only partially deiodinated (Oleksy-Frenzel et al. 1995, 2000; Drewes et al. 2001; Schittko et al. 2004; Jekel and Grünheid 2005). The environmental behavior of these partially deiodinated ICM and ICM TPs is still poorly investigated, limiting reliable assessments of their persistence, mobility, and relevance in drinking water systems, especially in the context of drinking water production from bank filtrate.

This study addresses this gap by investigating how the degree of iodination affects biodegradability of IOP, DTZ, and ATIA under aerobic and nitrate-reducing conditions. Aerobic biodegradation was investigated in Zahn-Wellens tests (ZWTs), a standardized method to assess inherent biodegradability under aerobic conditions, conducted according to OECD Guideline 302 B (OECD 1992) using activated sludge from a municipal wastewater treatment plant in Berlin. Biodegradation under nitrate-reducing conditions was examined in aquifer material–water batch tests with material from the riverbank of a Berlin lowland river. For IOP and DTZ derivatives, biodegradation kinetics were evaluated based on the decrease in the parent compound concentration, expressed as degradation half-lives (DT_50_), together with TP analysis to assess iodination-dependent transformation patterns. ATIA derivatives were investigated only in the ZWTs, where dissolved organic carbon (DOC) was additionally monitored to assess mineralization.

## Materials and methods

### Chemicals, standards and solvents

Information on chemicals, standards and solvents used for this study is provided in the Appendix A.1. Due to the lack of standards all partially and completely deiodinated compounds used were produced by reductive deiodination using the triiodinated compound as initial substance. The procedure is described in Bartels et al. (2023).

### Biodegradability tests

#### Zahn-Wellens tests

The ZWTs followed OECD Guideline 302 B (OECD 1992). Activated sludge was taken from the aerated zone of the Ruhleben wastewater treatment plant (Berlin, Germany; dry weather capacity: 247,500 m^3^/d; biological phosphate removal, nitrification, and denitrification; Berliner Wasserbetriebe 2025). Sludge for IOP and DTZ derivatives (batch 1) and for ATIA derivatives (batch 2) was collected on 15 May 2019 and 12 May 2020, respectively, with a dry matter content of 4.2 g/L in both batches. The sludge was washed twice with tap water, suspended in mineral medium, and stirred and aerated at room temperature prior to use. At the beginning, the dry matter content in all vessels was 1 g/L.

Single-compound tests with triiodinated or deiodinated compounds and multi-compound tests with mixtures of triiodinated, diiodinated, monoiodinated, and deiodinated derivatives of one compound were conducted in triplicate. The volume for ICM derivative tests was 200 mL, with initial single-compound concentrations of 12.6 µM (= 10 mg/L IOP). Multi-compound mixtures varied between 0.9 and 17.0 µM (0.4–10.0 mg/L) due to self-production, with no DTZ detected and low IOP–3I concentrations. For ATIA derivatives, the test volume was 500 mL to allow additional DOC quantification. Initial concentrations were adjusted to 10 mg/L DOC (excluding background DOC), corresponding to 58.2 mg/L ATIA and 18.9 mg/L ATIA–3I in single-compound tests and 4.1–14.5 mg/L in multi-compound tests.

Sterile controls were run in parallel: one without sludge and one with sludge plus formaldehyde (37 wt%, 10% of total volume) to inhibit biological activity. The first sampling (t₀) occurred 3 h after start, when sorption equilibrium to activated sludge is typically reached (OECD 1992). Microbial activity was verified by degradation of aniline (10 mg/L).

Tests were performed in a temperature-controlled room (18–22 °C) in the dark under gentle stirring and aerated via compressed air. Evaporation losses were monitored by weighing and compensated with deionized water before sampling. Before sampling, stirring and aeration were stopped to allow sludge settling; 1 mL of supernatant was filtered (0.45 µm). For ATIA tests, an additional 8 mL was taken on selected dates for DOC analysis to assess mineralization. On selected dates, pH and oxygen were measured in all vessels. Due to self-production of derivatives, titanium(III) citrate and cyanocobalamin were present (Table A.1) and were also added to tests with triiodinated compounds and aniline for consistency. Depending on the compound, experiments lasted 95–267 d.

#### Aquifer material–water tests

For biodegradability tests under nitrate-reducing conditions, aquifer material was collected in spring from the water-saturated zone (4–5 m depth) at two locations along a transect perpendicular to the urban lowland river Erpe (Berlin, Germany). The site, including geological setting, redox conditions, and groundwater chemistry, is described in detail by Schaper et al. (2025). Briefly, the aquifer exhibits predominantly sub-oxic to anoxic redox conditions, and the river is strongly impacted by effluent from the Münchehofe municipal wastewater treatment plant (Brandenburg, Germany). River water contains elevated nitrate (28.3 mg/L), DOC (11.8 mg/L), and various trace organic compounds, including ICM (Schaper et al. 2025). Redox potential-indicating ions in river water and bank filtrate are summarized in Table B.1.1.

Immediately after sampling, wet aquifer material was stored in airtight bags. One bag was opened for determination of ignition loss, fraction of organic carbon (f_OC_), pH (0.01 M CaCl₂), and grain size distribution (Table B.1.2). Pore water was also analyzed for ICM and known TPs. All pretreatments and biodegradability tests were conducted in an oxygen-free glove box (GS E-Line, Glovebox Systemtechnik, Maisch, Germany) under nitrogen atmosphere (0.1–1.0 ppm O₂). Aquifer material was sieved (< 2 mm) without washing to preserve the bacterial community.

For each test, 50 ± 0.05 g of aquifer material was combined with 250 mL of mineral medium (OECD 1992) supplemented with sodium nitrate to establish and maintain nitrate-reducing conditions, with target nitrate concentration based on river water values. Initial sorption was calculated from the concentration difference between t₀ and 48 h, which was selected as sorption equilibrium to sandy aquifer material is essentially reached within this period (Bartels et al. 2023). The 48 h value was used as the starting point for biodegradation tests. Tests were run in duplicate, with initial concentrations of 7.0–10.5 mg/L (IOP mixture) and 6.5–10.0 mg/L (DTZ mixture). The glove box temperature ranged from 18 to 25 °C.

Samples were periodically collected to monitor parent compound degradation and TP formation. On each sampling day, 1 mL was filtered (0.45 µm) into vials, removed from the glove box, and stored at 5–7 °C until analysis. Redox-indicating ions in suspensions were monitored (Table B.1.3). As analysis of manganese and iron required a high sample volume (10 mL), only three samples were collected per test; nitrate and sulphate were measured more frequently from 1 mL samples. Experiments lasted up to 140 d.

### Analyses

IOP, DTZ, ATIA, and their diiodinated, monoiodinated, and deiodinated derivatives were analyzed by liquid chromatography coupled to positive electrospray ionization mass spectrometry (LC–pos–ESI–MS; HP 1100, Agilent, Waldbronn, Germany; Quattro Micro, Waters, USA) operated in scan mode (m/z 80–900). LC–neg–ESI–MS in selective ion monitoring (SIM) mode for m/z 127 (iodide), combined with in-source fragmentation, was used to verify iodinated TPs by comparing retention times in LC–pos–ESI–MS and LC–neg–ESI–MS chromatograms (Putschew and Jekel 2003; Hütteroth et al. 2007). All operational parameters are given in Table A.2.1. High-performance liquid chromatography with UV detection (HPLC–UV; Agilent 1200 Series) was used for the quantification of aniline (Appendix A.3). The concentration of DOC was quantified using varioTOC Cube (elementar Analysensysteme, Germany). Sulphate and Nitrate were quantified by ion chromatography (IC, 930 Compact IC FLEX, Metrohm, Filderstadt, Germany), manganese and iron were quantified by flame atom absorption spectroscopy (AAS). All samples were filtered (0.45 µm) immediately after sampling for analysis, with AAS samples further acidified. The f_OC_ of the aquifer material was determined by CNS-Analyser Vario EL III (Elementar, Langenselbold, Germany).

### Calculations

DT_50_ was used to quantify the time required for a 50% decrease in the parent compound concentration, as derived from the rate constant (*k*). Two models were applied. The exponential approach (Eq. 1), assuming pseudo–first-order kinetics, uses normalized concentrations (*c*_*start*_ = 1) or MS signal areas to calculate DT_50,exp_ (Eq. 2).

1 $$ c_{t} = c_{start} \times e^{ - kt} $$

2 $$ DT_{50, \exp } = \frac{\ln 2}{{k }} $$

Because lag phases (*λ*) can cause underestimation of *k* (Filter et al. 2023), the modified Gompertz model (Eq. 3) proposed by Gibson et al. (1987) was also applied, where k is the relative growth rate at *t* = *L* (lag phase until the inflection point). All values were calculated using normalized data (*c*_*start*_ = 1, *c*_*end*_ = 0), and DT_50,mod.Gomp_ was derived as shown in Eq. (4).

3 $$ c_{t} = c_{start} + \left( {c_{end} - c_{start} } \right) \times e^{{ - e^{{ - k\left( {t - L} \right)}} }} $$

4 $$ DT_{50,\bmod . Gomp} = \frac{{ - \ln \left( { - \ln \left( {0.5} \right)} \right)}}{k} + L $$

Following Filter et al. (2023), DT_50,exp_ and DT_50,mod.Gomp_ are comparable; here, DT_50,exp_ was used for *λ* < 1 d and DT_50,mod.Gomp_ for *λ* ≥ 1 d (Eq. 5).

5 $$ \lambda = L - \frac{1}{k} $$

The DOC concentration was normalized according to Eq. (6), where c_blind_ represents the compound concentration in the suspension without activated sludge. As the multi-compound test suspensions contained titanium(III) citrate (TiCi) and cyanocobalamin (B12), their respective DOC concentrations (c_B12_ and c_TiCi_) were included in the normalization. In contrast, these concentrations were set to 0 in the single-compound tests.

6 $$ norm. c_{DOC} = \frac{{c_{t} - c_{t,blind} + c_{3h,B12} + c_{3h,TiCi} }}{{c_{3h} - c_{3h,blind} + c_{3h,B12} + c_{3h,TiCi} }} $$

## Results and discussion

In this study, the following terminology is applied consistently: Triiodinated denotes the parent compound containing three iodine atoms. Diiodinated (compound-1I), monoiodinated (compound-2I), and deiodinated (compound-3I) derivatives refer to TPs that have lost one, two, or all three iodine substituents, respectively.

### ZWTs

#### Validity of the ZWTs

In order to validate the microbial performance of the activated sludge used for the ZWTs, the biodegradability of aniline known as a well aerobically biodegradable compound was checked. Aniline was no longer detectable after 7 d at the latest using the two activated sludges taken at different days (Fig. B.2.1), confirming the functional capability of the microorganisms used for the ZWTs (OECD 1992).

The concentration of dissolved oxygen and the pH were measured throughout the experiment. The dissolved oxygen concentration in all tests ranged between 7 and 9 mg/L (Figs. B.2.2, B.2.3), and thus exceeded the required minimum threshold of 1 mg/L (OECD 1992). The pH values of all test suspensions were generally around pH 7, with only minor deviations below 6.5 (Figs. B.2.2, B.2.3), thus meeting the pH criteria specified in the Guideline 302 B.

#### Initial sorption and abiotic degradation

The initial sorption to activated sludge was quantified by the difference in compound concentration between the sterile control and the test suspension 3 h after the start, as sorption processes are much faster compared to the initial biotransformation step and an sorption equilibrium can be assumed (Joss et al. 2006). Due to the analytical quantification error demonstrated by the coefficient of variation (CV) plotted over concentration (Figs. A.2.1–2.3), sorption was only proven if the deviation of the compound concentration has been higher than the analytical error and positive. However, this was only possible for the triiodinated and deiodinated compounds for which standards were available. For the monoiodinated and diiodinated compounds, a 10% concentration decrease within the first 3 h was defined as the threshold for indicating initial sorption. Significant sorption was found for IOP–3I (16.4%) and ATIA–3I (9.5%) in the single-compound tests and ATIA–1I and ATIA–2I A (16.2–18.2%) in the multi-compound tests (Table B.2.1). All other compounds showed no significant initial sorption. In general, the results are in line with those observed in other studies (Bartels et al. 2023, 2024) showing that partially and completely deiodinated derivatives have a higher sorption affinity to organic material compared to their corresponding triiodinated compound. It is assumed that the three large iodine atoms cause steric hindrance and repulsive forces and therefore a (partial) deiodination increases the sorption affinity (Bartels et al. 2023). The very low or negligible sorption of DTZ and IOP to activated sludge observed in this study is in agreement with other studies (Ternes et al. 2004; Batt et al. 2006; Echeverría et al. 2014).

The presence of abiotic degradation was assessed by monitoring the compound concentrations in the sterile tests. No significant decrease in concentration was observed in the sterile tests for all compounds examined (Figs. B.2.4–2.6).

#### Initial biotransformation step

The influence of the number of bound iodine atoms on the initial aerobic biotransformation step of the selected compounds, was assessed using DT_50_ determined by the single- and multi-compound ZWTs. The mean DT_50_ and *λ* values for the models employed are presented in Table 1. Detailed values, model parameters, and plotted concentration curves with corresponding fittings are provided in the Appendices (Table C.1.1; Figs. C.1.1–C.1.6). Table 1 also lists the initial compound concentrations. In the multi-compound tests, these varied due to (i) self-produced derivative mixtures and (ii) specific experimental objectives. For IOP and DTZ, the focus was on the initial biotransformation step across iodination levels, whereas ATIA derivatives required higher concentrations to allow measurable DOC mineralization. For ATIA-2I, two isomers were detected, though the iodine position remains unknown.

**Table 1:** DT_50_ values (means and standard deviations of triplicates) for IOP, DTZ and ATIA as well as their diiodinated (–1I), monoiodinated (–2I), and deiodinated (–3I) derivatives determined in ZWTs

Test	Compound	Initial concentration (µM)	DT_50_ (d)	*λ* (d)	R^2^	Model
Single-compound	IOP	15.0 ± 0.3	0.85 ± 0.02	< 1.00	1.00 ± 0.00	1st Order Decay
	IOP–3I	16.9 ± 0.4	0.80 ± 0.02	< 1.00	1.00 ± 0.00	1st Order Decay
	DTZ^a^	16.2 ± 0.5	65.0	54.7	0.82	Mod. Gompertz
	DTZ–3I	23.3 ± 0.1	1.17 ± 0.18	< 1.00	0.96 ± 0.02	1st Order Decay
	ATIA	103.0 ± 4.7	44.0 ± 8.7	9.40 ± 1.36	0.95 ± 0.02	Mod. Gompertz
	ATIA–3I	100.4 ± 1.8	15.0 ± 0.9	13.6 ± 1.4	1.00 ± 0.00	Mod. Gompertz
Multi-compound	IOP	3.6 ± 0.2	1.16 ± 0.05	< 1.00	1.00 ± 0.00	1st Order Decay
	IOP–1I	⁓16^c^	0.96 ± 0.07	< 1.00	0.99 ± 0.00	1st Order Decay
	IOP–2I	⁓16^c^	1.09 ± 0.01	< 1.00	0.99 ± 0.00	1st Order Decay
	IOP–3I	0.9 ± 0.0	0.71 ± 0.02	< 1.00	1.00 ± 0.00	1st Order Decay
	DTZ	< LOD	n.d	n.d	n.d	–
	DTZ–1I^b^	⁓10^c^	59.4 ± 7.6	50.2 ± 4.4	0.88 ± 0.02	Mod. Gompertz
	DTZ–2I	⁓17^c^	5.76 ± 0.63	< 1.00	0.99 ± 0.00	1st Order Decay
	DTZ–3I	5.0 ± 0.3	0.88 ± 0.19	< 1.00	0.99 ± 0.01	1st Order Decay
	ATIA	15.1 ± 0.7	20.4 ± 2.1	6.22 ± 2.61	0.97 ± 0.01	Mod. Gompertz
	ATIA–1I	⁓21^c^	35.6 ± 3.6	10.6 ± 3.5	0.98 ± 0.00	Mod. Gompertz
	ATIA–2I A	⁓45^c^	0.30 ± 0.03	< 1.00	1.00 ± 0.00	1st Order Decay
	ATIA–2I B		41.6 ± 1.7	20.9 ± 2.1	0.98 ± 0.01	Mod. Gompertz
	ATIA–3I	21.8 ± 1.4	12.0 ± 1.3	5.00 ± 1.30	0.97 ± 0.00	Mod. Gompertz

*n.d.* not determined

^a^complete degradation occurred only in one triplicate

^b^mean and deviation of two triplicates

^c^calculated concentration

The λ values indicated that there was no lag phase (*λ* < 1 d) for all tested IOP derivatives and tests (Table 1), indicating immediate biodegradation. In contrast, a lag phase was recognized for DTZ in the single-compound tests and for DTZ–1I in the multi-compound tests showing *λ* values from 37 to 40 d. For ATIA, ATIA–1I, ATIA–2I B and ATIA–3I comparatively shorter lag phases were observed in both tests, with *λ* ranging between 5.0 and 20.9 d. In contrast, *λ* for ATIA–2I A was very low.

The observed differences in lag phases suggest varying microbial adaptation times depending on the compound structure (Swinnen et al. 2004; Bertrand 2019). For IOP derivatives, immediate degradation indicated that the required enzymes for the initial oxidation at a side chain carbon were already present and not sterically hindered. Alternatively, it is also conceivable that the initial transformation step is catalyzed by unspecific oxygenases. In contrast, the pronounced lag phases for DTZ, DTZ–1I and all ATIA derivatives with exception of ATIA–2I A point to the need for microbial adaptation, potentially due to steric hindrance from iodine atoms. For DTZ, the initial transformation is a deacetylation of amide groups (Kalsch 1999), which may be affected by iodine positioning. However, the position of the iodine atom(s) in the partially deiodinated compounds was not investigated. The exceptional behavior of ATIA–2I A, which degraded without delay, further suggests that structural features and reaction types strongly influence the adaptation time.

Overall, Table 1 shows a rapid decrease in concentration for all tested IOP derivatives, showing DT_50_ values in the range of 0.71 (IOP–3I) to 1.16 d (IOP). In contrast, those of DTZ and its partially and completely deiodinated derivatives showed high variations in DT_50_ over all tests ranging from 0.88 (DTZ–3I) to 65.0 d (DTZ, in only one triplicate). High variations were also observed for ATIA and its derivatives from 0.30 (ATIA–2I A) to 44.0 d (ATIA). The results generally indicate that in most of the cases iodine significantly increases the DT_50_ values and thus decreases the degradation rates of the tested compounds, although this effect varies depending on the type of compound.

A comparison of the determined DT_50_ values for IOP (Table 1) shows good agreement with those from other studies using activated sludge, where degradation half-lives calculated under the assumption of first-order degradation ranged from a few hours to 3 d (Kalsch 1999; Joss et al. 2006; Pérez et al. 2006). However, the results for DTZ differ considerably from previous studies, reporting from no transformation within 54 d (Kalsch 1999) to complete degradation within 23 d after a shorter adaptation period (Haiss and Kümmerer 2006). These discrepancies, observed under varying inocula (e.g., activated sludge or sediment) and initial concentrations, demonstrate a high dependency of the initial transformation of DTZ on experimental conditions, such as different bacterial community composition or initial compound concentration. Concerning the latter, it was reported that degradation times of IOP decreases with increasing initial concentrations (Kalsch 1999; Steger-Hartmann et al. 2002). However, it must also be considered that at lower concentrations, degradation is apparently quick as concentrations rapidly fall below the LOQ or LOD. This leads to higher calculated degradation rates and eventually to misinterpretation of the degradation. An example for this is IOP–3I of the multi-compound tests showing the fastest degradation of all derivatives and the lowest initial concentration used among all tested compounds (Table 1). For this reason, degradation rates are compared only within individual tests in this study.

In the single-compound tests, the pronounced decrease in DT_50_ values from DTZ to DTZ–3I (98%) as well as from ATIA to ATIA–3I (66%) demonstrated considerable stability of the triiodinated structure to aerobic biodegradation, which was attributed to the presence of the three iodine atoms, whereas deiodination had only a minor effect on the initial transformation of IOP (decrease of about 6%) (Table 1). This difference was attributed to the steric hindrance by the iodine atoms, which interfered with certain initial transformation mechanisms such as the amide hydrolysis near the aromatic ring. This hypothesis is consistent with findings from other studies, which reported that the side chain transformation of IOP and DTZ was influenced by iodine’s steric hindrance, significantly delaying critical amide hydrolysis near the aromatic ring (Redeker et al. 2014, 2018). Helbling et al. (2010) also observed steric hindrance of amide hydrolysis in case of o,o-disubstituted cyclohexyl groups under aerobic conditions, which are structurally comparable to our tested benzene derivatives. These findings agreed with the effects observed for ATIA and DTZ, but deviated from those for IOP. IOP, being a larger molecule than DTZ and ATIA, has long aliphatic side chains. We assume that steric hindrance is of minor meaning on the initial aerobic biotransformation step of iodinated derivatives of IOP, as the responsible initial transformation is not influenced by the iodinated aromatic ring. The distance between the bound iodine atoms and the initial reaction site likely minimizes the impact of steric effects on enzymatic access to the side chains.

In the multi-compound tests, the impact of (partial) deiodination on DT_50_ values varied significantly among the degree of iodination (Table 1). In most cases, however, there was no clear correlation between DT_50_ values and the number of bound iodine atoms. For IOP derivatives, DT_50_ differences were small and difficult to interpret, as models were fitted using only three data points due to rapid degradation. IOP–3I exhibited a significant DT_50_ decrease from 1.16 to 0.71 d (− 39%), while IOP–1I and IOP–2I had values of 0.96 and 1.09 d, respectively. Although no clear trend with iodine substitution emerged, the slight but significant difference for IOP–3I suggests a marginally faster degradation.

Distinct differences were observed among the tested derivatives of ATIA. While ATIA had a DT_50_ of 20.4 d, that of ATIA–1I as well as ATIA–2I B increased to 35.6 d and 41.6 d, respectively. Conversely, the DT_50_ values for ATIA–2I A and ATIA–3I were significantly lower with 0.30 and 12.0 d, respectively. These pronounced variations in DT_50_ values among the ATIA derivatives suggested that degradation kinetics were influenced not only by steric effects but also by the electronic effects and position of the iodine substituents (Häggblom 1992). Notably, all ATIA derivatives possess three functional groups, namely two carboxy groups and one amide group, directly attached to the aromatic ring. At this point, ring cleavage may be the next transformation step in the degradation process of halogenated aromatic compounds (Pimviriyakul et al. 2020). In this context, the electron-withdrawing effect of iodine plays a decisive role: halogen substituents reduce the nucleophilic character of the aromatic ring, thereby hindering electrophilic attack at the ring by dioxygenase enzymes and decreasing degradation rates (Dorn and Knackmuss 1978). These electronic effects are further dependent on the presence and position of other functional groups bound to the ring (Andreozzi et al. 2006). An evidence for the presence of multiple factors influencing the degradation is shown in Fig. C.1.7 showing no linear correlation between the theoretical primary biodegradation value (BIOWIN4) estimated using EPI Suite™ v4.11 (U.S. Environmental Protection Agency 2012) and experimentally determined k. The BIOWIN4 values are based only on electronic effects.

In contrast to IOP and ATIA, all DTZ derivatives demonstrated a decreasing trend in DT_50_ values with each step of partial deiodination in the multi-compound tests. The DT_50_ value decreased from 59.4 d for DTZ–1I to 5.76 d for DTZ–2I, and further to 0.73 d for DTZ–3I, representing a decrease of approximately one log order with each iodine atom removed. This consistent pattern indicates that steric hindrance by iodine is a dominant factor controlling the kinetics of DTZ degradation. Notably, DTZ data from the multi-compound test are missing as it was not detectable; however, the DT_50_ value of DTZ determined in the single-compound test (Table 1) aligns well with this trend.

#### Detection of TPs

##### TPs of IOP, IOP–1I, IOP–2I and IOP–3I

**Fig. 1:**
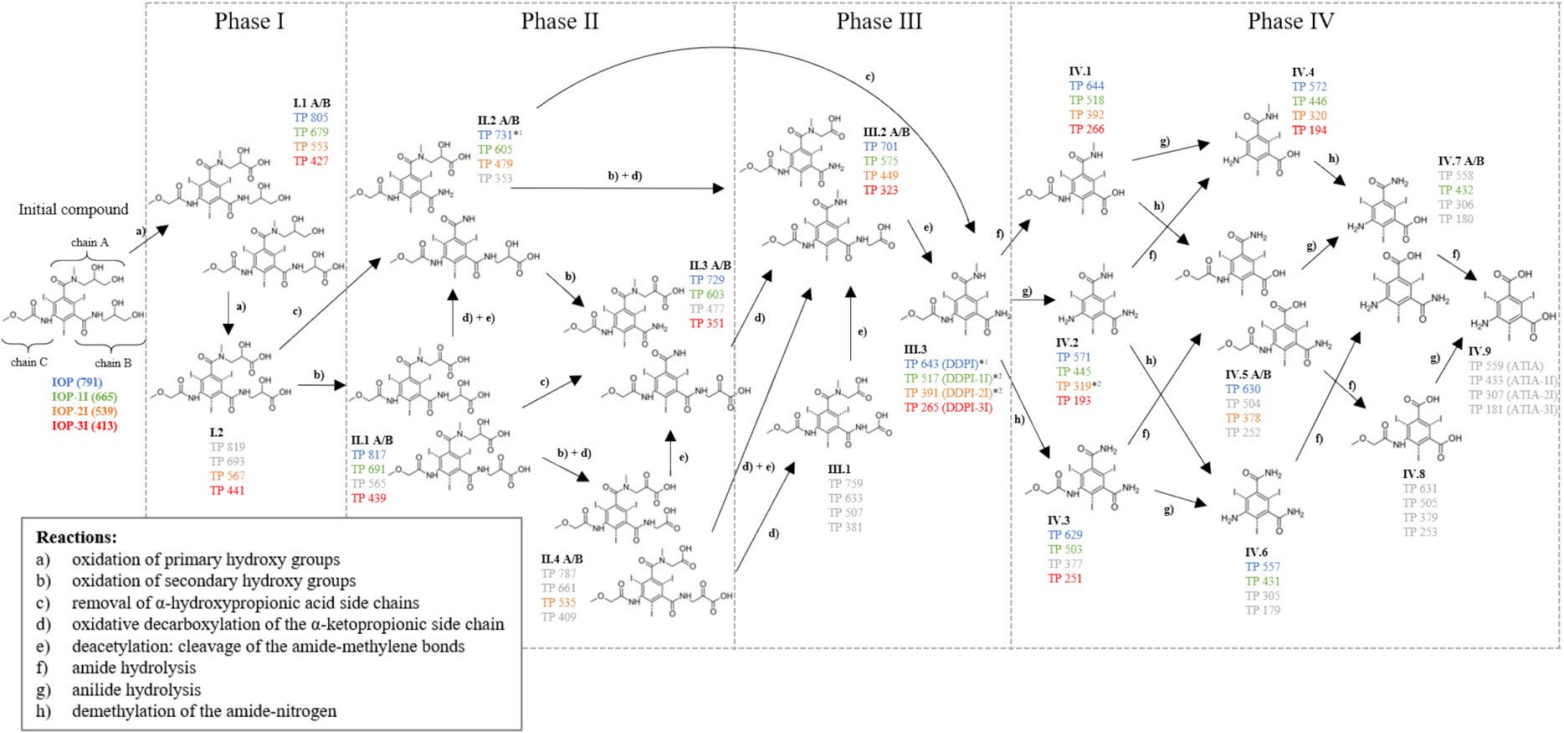
Aerobic transformation pathway of IOP (blue), IOP–1I (green), IOP–2I (orange), and IOP–3I (red). Data were compiled from the single- (IOP, IOP–3I) and multi-compound tests (IOP, IOP–1I, IOP–2I). Detected m/z values are presented as molecular masses in the color corresponding to the initial compound; grey masses were not detected. *^1^Masses detected only in the multi-compound tests. *^2^Two isomers were identified. (Color figure online)

Figure 1 shows the transformation pathway of IOP and its diiodinated, monoiodinated, and deiodinated derivatives. The pathway is summarized for IOP and IOP–3I from the single-compound tests, and for IOP, IOP–1I, and IOP–2I from the multi-compound tests. It is divided into four phases: the pathway including the TPs shown in Phase I–III was described by Schulz et al. (2008) and later subdivided into three phases by Kormos et al. (2011), while phase IV, including the proposed TPs, was developed in this study. Figure 1 also includes data from both single- and multi-compound tests. Detected m/z ratios (given as molecular masses) are highlighted in color. For orientation, the TPs were numbered using the phase number (Roman numeral) followed by consecutive numbers per phase (Arabic numeral). The concentration curves of the TPs over time are shown in Figs. C.1.8–C.1.27.

Given that the chains A and B of the IOP derivative structure differ only by the methylated amide-nitrogen of chain A, identical transformations can occur on both chains, resulting in constitutional isomers. These TPs were not differentiated in this study and the isomers were labelled with A and B in the pathway (Fig. 1).

In general, the detection of all proposed TPs of phase I-III given in Fig. 1 was incomplete; only the initial TP I.1 and the last two TPs from Phase III (TP III.2 and III.3) were identified for all iodination levels. With exception of TP III.1, for all other TPs (I.2, II.1, II.2, II.3 and II.4) at least one mass was detected. The detected masses appeared consistently and simultaneously across replicates (Figs. C.1.8–C.1.27).

Since both the initial TP I.1 and the final TP III.3 (derivatives of DDPI) of phase III were observed for all iodination levels, we conclude that (partial) deiodination of IOP does not significantly affect the aerobic biological transformation process. However, the absence of some intermediate TPs suggests that certain bonds or structures may be less stable in specific derivatives. As a result, transformation kinetics may be too fast for these TPs to reach signal intensities above the LOD. If different types of reactions occur and lead to distinct TPs, the kinetics determine the concentration ratios between them.

The influence of functional groups on the stability of the side chain has been previously proposed, with a higher stability of the amide–methylene bond in side chain A compared to chain B attributed to the presence of a methyl group at the amide moiety (Schulz et al. 2008). However, our data did not clearly show whether the number of bound iodine atoms affected the preference for specific transformation reactions. Regarding transformation kinetics, no significant influence was observed: TPs appeared simultaneously across the first, second, and third transformation phases, regardless of the iodination degree (Figs. C.1.8–1.27). Notably, TP III.3 was consistently detectable around day 20 for all iodination levels (Figs. C.1.14, C.1.18, C.1.22, C.1.26).

Contrary to the findings of Schulz et al. (2008), suggesting that TP III.3 643 (DDPI) is stable under aerobic conditions, a decline of concentration was observed for this TP as well as for the analogous TP III.3 517 (DDPI–1I) and III.3 391 (DDPI–2I) in the multi-compound tests (Figs. C.1.18, C.1.22, C.1.26) indicating further transformations within 95 d of the experimental period. Therefore, additional theoretical TPs were hypothesized and integrated into further transformation pathways shown in phase IV (Fig. 1). On the basis of the pathway prediction tool enviPath (Wicker et al. 2016) and Helbling et al. (2010) three different main reactions were suggested, all involving the primary and secondary amid structure: (i) amide hydrolysis of chain A or B, (ii) anilide hydrolysis of chain C and (iii) demethylation of the amide-nitrogen of chain A. Prior to anilide hydrolysis, demethylation of the methoxy group of chain C followed by an oxidation of the aldehyde were considered as another potential transformation step, but no TPs resulting from this transformation were found (Fig. C.1.28). The phase IV pathways were developed up to the basic structure of ATIA.

In phase IV, the masses of seven TPs (TPs IV.1–7) were detected (Fig. 1). Whereas the masses of all iodination levels were found for TP IV.1, IV.2 and IV.4, not all possible masses were detected for TP IV.3, IV.5, IV.6 and IV.7. No masses were found for IV.8 and IV.9. Overall, each proposed reaction, including demethylation of the amid nitrogen of chain A, amide hydrolysis of chain B as well as anilide hydrolysis of chain C was observed for every iodination level. TP IV.7 was observed solely for the diiodinated compound (TP 432) and constitutes the most structurally altered transformation product of IOP detected thus far.

The occurrence of TPs resulting from amide and anilide hydrolysis contrasts with findings from previous studies, in which the amide groups of IOP and structurally similar ICM such as iohexol, iopamidol, and iomeprol remained stable during aerobic aquifer material–groundwater batch experiments (Schulz et al. 2008; Kormos et al. 2010). This discrepancy may be attributed to differences in the bacterial communities present in the inocula. Kalsch (1999) already observed different TPs formed in sludge experiments compared to sediment–water systems. Consistent with this, our results obtained using activated sludge corroborate the findings of Steger-Hartmann et al. (2002), who reported a TP of IOP formed by amide hydrolysis in side chain C in a simulated lab-scale sewage treatment plant.

As shown in Fig. 1, TP IV.4 was formed by amide and anilide hydrolysis. Its occurrence, irrespective of the number of bound iodine atoms, suggests that these reactions are not sterically hindered by iodine atoms. The hydrolysis of the primary amide in side chain B is plausible, as primary amides are known to be rapidly hydrolyzed due to the absence of a second substituent on the nitrogen atom (Helbling et al. 2010). This rapid transformation is reflected in the immediate formation of TP IV.1, which appeared as the first TP of phase IV following the transformation of TP III.3 in all triplicates (Figs. C.1.15, C.1.23, C.1.27). A similarly early appearance was observed for TP IV.2, resulting from anilide hydrolysis (same figures), which is consistent with the findings of Helbling et al. (2010), who reported a very fast reaction of anilides. Although the hydrolysis of secondary amides is known to be strongly influenced by the surrounding molecular structure, the presence of one, two or three iodine atoms in close proximity to the anilide nitrogen showed no observable effect on the reaction in this study.

Although demethylation of the amide nitrogen in side chain A was initially observed only for the triiodinated, diiodinated, and deiodinated structures based on the formation of TP IV.3, the detection of TP IV.5 A/B confirms that demethylation also occurred for the monoiodinated TP (Fig. 1). Given the significantly slower temporal appearance of TP IV.3 compared to TP IV.1 and TP IV.2, as well as TP IV.5 compared to TP IV.4, we suggest that demethylation is less favored by the microorganisms. These findings are consistent with those of Helbling et al. (2010), who reported that compounds containing tertiary amides were predominantly transformed by other reactions, such as oxidation, rather than N-dealkylation.

Summarized, there is no observed correlation between the different suggested reactions and the number of bound iodine atoms. However, from the observation that the detection of the masses of IV.3, IV.5, IV.6, and IV.7 was not possible for all iodination levels, an influence is obvious but not explainable based on the data. The most degraded TP observed (IV.7) was only found for the diiodinated structure (TP 432) during the experimental period of 130 d (Fig. 1). However, since the TP concentrations (or MS signal areas) decrease with increasing branching of the transformation pathways and thus increasing number of different TPs (down to below the LOD), it is difficult to reliably exclude the presence of TPs from other iodination stages in the further course of phase IV.

##### TPs of DTZ, DTZ–1I, DTZ–2I and DTZ–3I

**Fig. 2:**
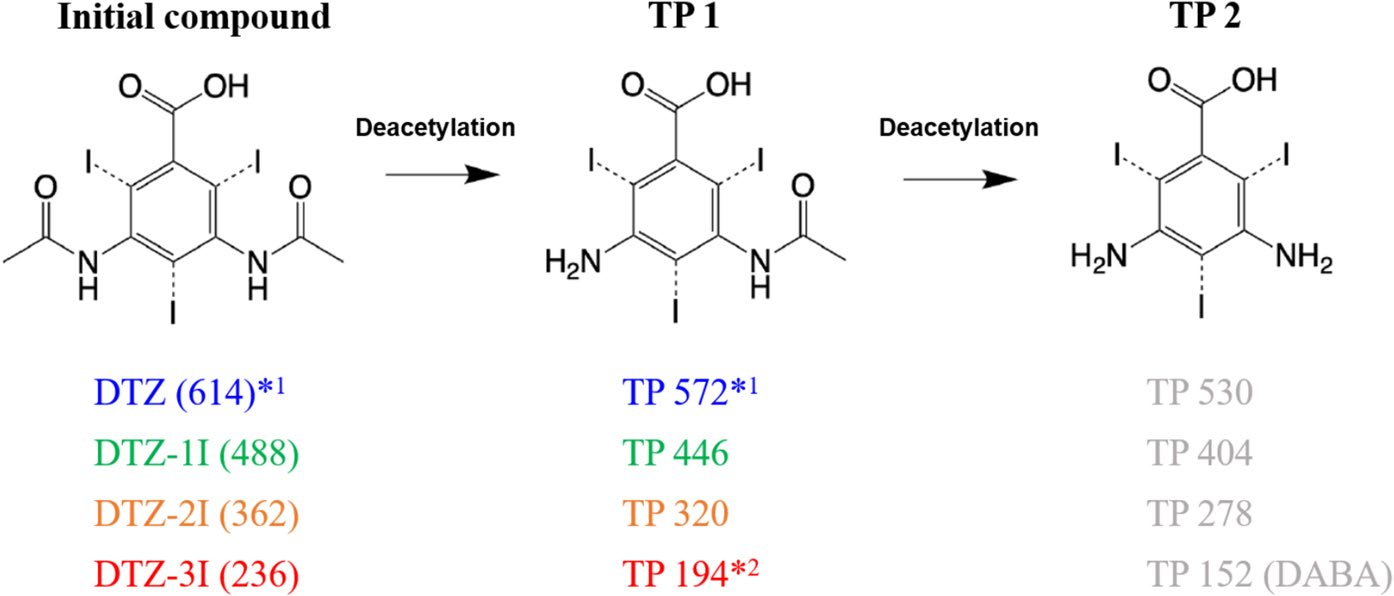
Aerobic transformation pathway of DTZ (blue), DTZ–1I (green), DTZ–2I (orange) and DTZ–3I (red) based on Kalsch (1999). Detected m/z values are presented as molecular masses and are shown in the color of the respective parent compound; grey masses were not detected. *^1^Masses detected only in the single-compound tests. *^2^Masses detected in both single- and multi-compound tests. (Color figure online)

The aerobic transformation pathway for DTZ, proposed by Kalsch (1999) is depicted in Fig. 2. Analogous to the triiodinated DTZ, the masses of the TPs are also shown for the corresponding diiodinated, monoiodinated, and deiodinated derivatives and are color-highlighted when detected. Figure 2 includes data from both single- and multi-compound tests. The concentration curves of the detected TPs over time are displayed in Figs. C.1.29–C.1.33. Two TPs known from the literature (Kalsch 1999; Haiss and Kümmerer 2006), named here TP 1 and TP 2, are products of the sequential deacetylation as a result of an amide hydrolysis.

Figure 2 shows, that only TP 1 was detected for all iodination levels of DTZ. The concentration curves given in Figs. C.1.29–C.1.33 show that TP 1 of DTZ–1I, DTZ–2I and DTZ–3I was unstable and underwent further transformation. A slight decrease in TP 1 concentration for DTZ was observed in only one replicate, which was attributed to the slow transformation of the parent compound (Fig. C.1.29). Since both TP 1 and TP 2 result from a deacetylation, the required enzymes are likely already expressed. Therefore, the formation of TP 2 was highly likely, even though it was not detected. One possible explanation is sorption to sludge, which had previously been observed for TP 2 152 (DABA) (Haiss and Kümmerer 2006). This is further supported by the fact that the sorption affinity of iodinated aromatic compounds to natural organic matter increases with successive deiodination (Bartels et al. 2023).

A search for further TPs derived from TP 2 of DTZ–2I and DTZ–3I using the pathway prediction tool enviPath (Wicker et al. 2016) did not yield any additional hypothetical TPs that were detectable in the ZWTs (Table C.1.2), which is consistent with observations from previous studies (Kalsch 1999; Haiss and Kümmerer 2006; Redeker et al. 2014). Redeker et al. (2014) suggested that TP 2 of DTZ–3I underwent either mineralization or complete assimilation into biomass, but noted that their chromatographic method was not optimized for low-molecular-weight, polar compounds. This methodological limitation may also explain why no further TPs were detected in our study.

Notably, analysis using the selective detection of organically bound iodine showed a peak with a retention time of 13 min at days 46 and 95 in all triplicates, which could not be assigned to any hypothesized TP (Table C.1.3; Figs. C.1.34–C.1.36). This indicates the formation of an unknown iodinated compound. Due to the absence of detectable TPs, a potential influence of iodine on the biodegradability of the tested DTZ derivatives could not be confirmed.

#### Mineralization

**Fig. 3:**
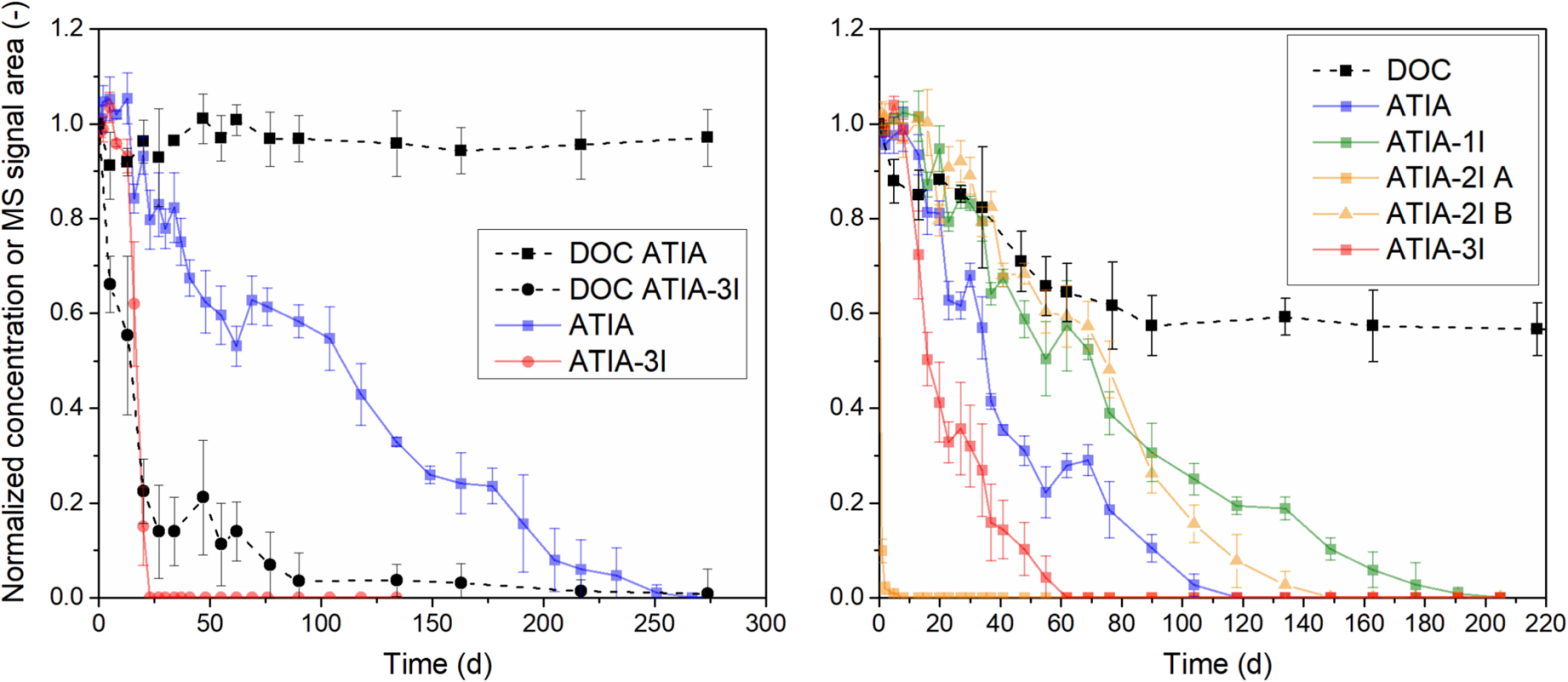
Normalized DOC and compound concentration in the single- (left) and multi-compound (right) ZWTs. DOC concentration was normalized according to Eq. 6. Initial DOC concentrations (mg/L) were 21.0 ± 0.2 (ATIA), 19.2 ± 0.4 (ATIA–3I) and 40.6 ± 0.5 (ATIA mix)

The influence of the number of bound iodine atoms on the mineralization of ICM TPs was assessed using ATIA and its diiodinated, monoiodinated, and deiodinated derivatives, which represent the final hypothesized TPs (IV 9) in the proposed IOP transformation pathway (Fig. 1). Figure 3 shows the normalized DOC concentrations from the single- and multi-compound ZWTs together with the corresponding compound concentrations.

In the ATIA single-compound tests, the DOC concentration remained nearly constant at approximately 96% throughout the 274-days test period, despite a continuous decrease in ATIA concentration, with the compound falling below the LOD after 250 d (Fig. 3). In contrast, the DOC concentration in the ATIA–3I tests decreased reaching a normalized concentration below 10% within 77 d. The concentration of ATIA–3I decreased too, indicating that ATIA–3I was transformed. Possible TPs, e.g. hydrolyzed carboxyl or amide groups could not be detected. The results indicate mineralization of ATIA–3I (Fig. 3), consistent with observations by Lecouturier et al. (2008), who reported mineralization of ATIA after deiodination. This is likely due to the absence of iodine substituents causing steric hindrance and electron-withdrawing effects, both known to inhibit ring-cleaving by oxygenase enzymes (Dorn and Knackmuss 1978). Potential aliphatic TPs were not found, possibly due to the low molecular weights of these TPs disappearing in the noise of the MS.

The normalized DOC concentration in the multi-compound ZWTs showed a decrease of about 40% within 90 d and then remained stable until the end of the test (205 d) (Fig. 3). ATIA–2I A was not detectable after 8 d and ATIA–3I after 60 d, whereas the other derivatives were detectable over more than 100 d. Due to differences in initial concentrations (Table 1), each derivative contributed differently to the total DOC: ATIA 21%, ATIA–1I 20%, ATIA–2I 43%, and ATIA–3I 16%. ATIA–2I was present as two isomers, whose individual DOC contributions could not be determined. Based on the results of the single-compound tests, ATIA–3I and ATIA–2I A were responsible for the observed DOC decrease, whereas the transformation of ATIA should not have affected the DOC concentration. It is likely, that the transformation of ATIA–1I and ATIA–2I B also influenced the DOC concentration. However, the stable DOC concentration after 90 d suggested that these transformations had only a minor effect on DOC.

### Nitrate-reducing aquifer material–water tests

#### Initial sorption

The initial decrease in compound concentration between the first sample (t_0_) and that after 48 h (t_1_) was evaluated in Table C.2.1 in terms of initial sorption. In general, significant sorption was observed for the deiodinated compounds and both aquifer materials, S1 and S2, used. The mean deviations of the concentration between t_1_ and t_0_ ranged from 11.8 to 16.3% and were significantly higher than the calibration CV at the corresponding concentration range (Table C.2.1). For all other derivatives, the small deviations between 0.1 and 7.5% were interpreted as no or no significant sorption. An exception is DTZ–2I and S2 indicating initial sorption of approximately 13.4%. In general, the results are in line with those observed for sandy aquifer materials showing higher sorption for partially and completely deiodinated derivatives compared to the triiodinated compound (Bartels et al. 2023).

#### Transformation of IOP derivatives

In general, the tested compounds IOP, IOP–1I, IOP–2I, and IOP–3I were primarily degraded within a similar time range following a distinct lag phase (Table C.2.2). The DT_50_ values in the suspensions using aquifer material S1 exhibited greater variability between duplicates (23.9–74.4 d) compared to S2 (38.7–42.3 d). *λ* ranged from 12.5 to 76.8 d in S1 and from 32.5 to 38.9 d in S2. Compared to the results of the ZWTs, these values are considerably higher (Table 1), indicating a significantly slower transformation of the IOP derivatives in the aquifer material–water tests under nitrate-reducing conditions, as well as a pronounced adaptation period. This extended lag phase may also be related to the presence of biodegradable DOC in the pore water (Table B.1.1), which may have been preferentially metabolized by the microbial community prior to IOP.

**Fig. 4:**
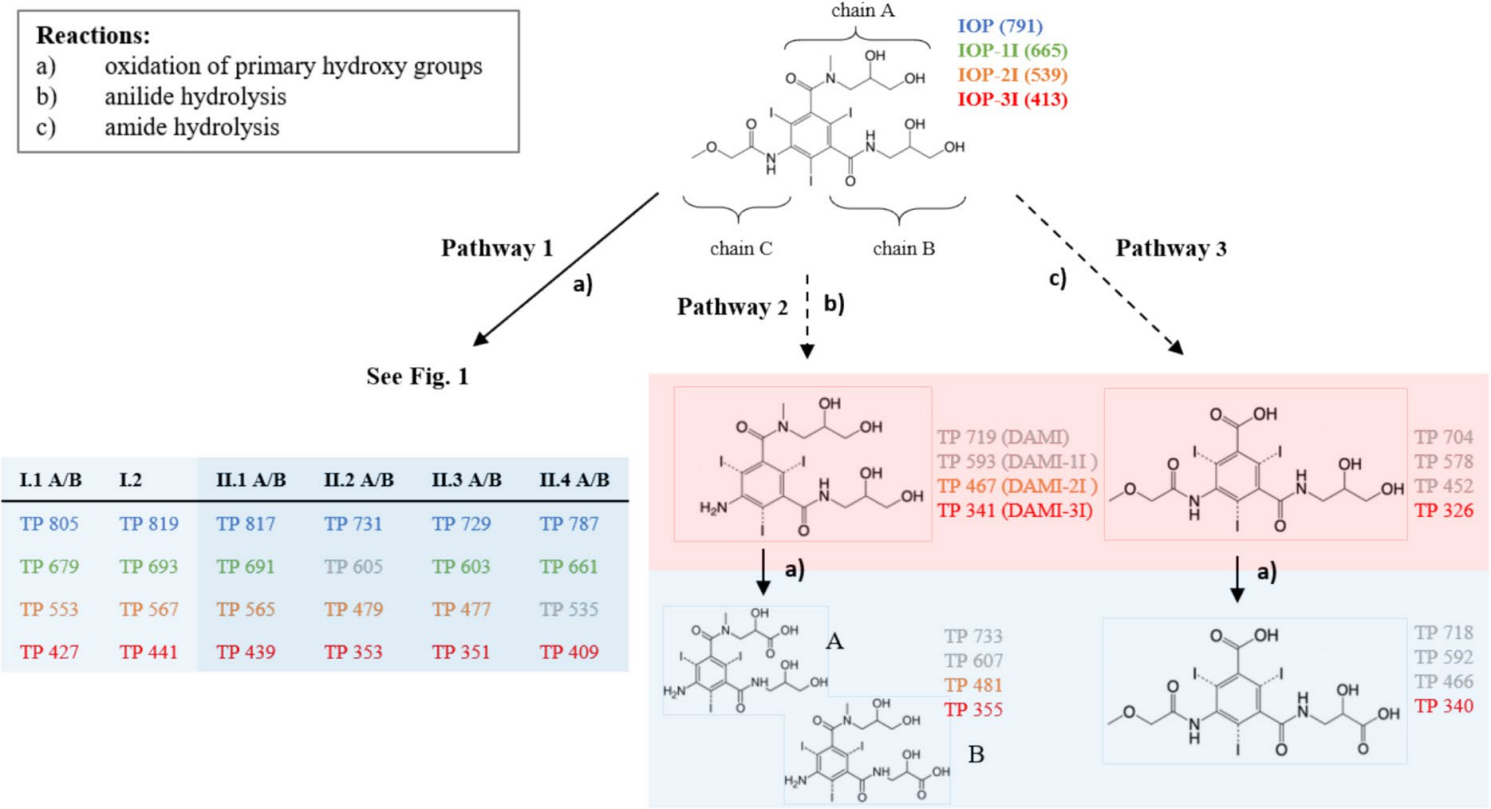
TPs detected during the nitrate-reducing water–aquifer material tests and possible transformation pathways. TPs resulting from aerobic reactions are shown with a blue background, those from anaerobic reactions with a red background. Detected m/z values are presented as molecular masses and are shown in the color of the respective initial compound; grey masses were not detected. Dashed arrows indicate hypothetical connections that have not been confirmed

All TPs found during the course of the tests are color-highlighted in Fig. 4. Prior to the tests, investigations of the pore water of S1 and S2 showed none of the initial compounds used, but the masses 467, 341 and 326 were detected in both aquifer material pore water samples (Fig. 4). These are likely ICM-related TPs. The masses 467 and 341 are known as anaerobic TPs of DAMI (Redeker et al. 2018). Both structures result from reductive (partial) deiodination and anilide hydrolysis of chain C. In TP 326 chain A was replaced by a carboxy group due to hydrolysis (Fig. 4). This transformation was previously observed under anaerobic conditions (Redeker et al. 2018). The pore water analysis indicated that IOP TPs originating from treated wastewater effluent had entered the Erpe River and subsequently infiltrated into the bank. The data further suggested that anaerobic transformations had already occurred along this pathway. Therefore, we assume that the biological community in the aquifer is well adapted to the transformation of ICM.

Based on the detected TPs and their time of occurrence (Figs. C.2.1–C.2.8), three potential pathways were proposed (Fig. 4). Pathway 1 follows the same sequence of reactions as the aerobic transformation pathway and includes the formation of several phase I and II TPs (Fig. 1). Among them, TPs II.3 A/B and II.4 A/B represent the most advanced transformation observed in the tests and were detected across nearly all iodination levels.

In contrast, pathways 2 and 3 involve TPs that were already present in the pore water at the beginning of the experiment (Fig. 4). Pathway 2 includes TP 467 (DAMI–2I) and TP 341 (DAMI–3I), as well as one further TP for each compound: TP 481 and TP 355, both of which likely resulted from the oxidation of a primary hydroxyl group either at chain A or chain B. However, their concentration profiles suggest that they were not stable throughout the test period, and no subsequent potential TPs were detected (Figs. C.2.3, C.2.4, C.2.7, C.2.8). Pathway 3 builds on TP 326 (Fig. 4), which was also initially present. Here, one follow-up product, TP 340, was detected, which likely formed via oxidation of a primary hydroxyl group. Similar to Pathway 2, TP 340 was unstable, and no further potential TPs were observed (Figs. C.2.3, C.2.8).

In both pathways 2 and 3, the concentrations of the already present TP 467 (DAMI–2I), TP 341 (DAMI–3I), and TP 326 initially increased (Figs. C.2.3, C.2.4, C.2.7, C.2.8). It could not be conclusively verified whether these compounds were directly connected to the applied IOP–2I and IOP–3I, or whether undetected intermediates were involved.

In summary, aquifer material–water tests with IOP derivatives showed that aerobic transformation pathways occurred also under nitrate-reducing conditions and, analogous to the ZWTs results, are unaffected by the number of iodine atoms. In contrast, only anaerobic TPs that had undergone single or double reductive deiodination were subject to subsequent oxidation under nitrate-reducing conditions. This demonstrates that under changing redox conditions, monoiodinated and deiodinated IOP TPs can follow a broader range of transformation pathways than initially assumed.

#### Transformation of DTZ derivatives

In contrast to the results obtained from the ZWTs, both DTZ and DTZ–1I remained stable during the nitrate-reducing aquifer material-water tests (110 d). This is in agreement with previous anaerobic findings showing that transformation at the amide-containing side chains requires cleavage of at least two iodine atoms (Redeker et al. 2014). However, the constant iodide concentration measured in all test suspensions (not shown) suggests that no deiodination took place under the prevailing nitrate-reducing conditions.

DTZ–2I and DTZ–3I underwent deacetylation during the nitrate-reducing tests, exhibiting comparable DT_50_ values between 5.60 and 8.24 d (Table C.2.2). TP 1 and TP 2 were detected for these derivatives in the nitrate-reducing aquifer material–water suspensions. Notably, TP 1 of DTZ–2I was already present in the pore water of both aquifer materials at the start of the experiment (Figs. C.2.13–C.2.16), indicating its formation during bank filtration at the Erpe river site. TP 2 of DTZ–2I and DTZ–3I (DABA) remained stable throughout the experiment. The persistence of TP 2 of DTZ–3I was previously reported in sediment–water tests under anaerobic conditions (Redeker et al. 2014) as well as under aerobic conditions (Kalsch 1999; Haiss and Kümmerer 2006). The comparison of the DT_50_ values and time of appearances of TP 2 between DTZ–2I and DTZ–3I suggests that the remaining iodine atom in DTZ–2I did not exert a significant influence on the two sequential deacetylation steps in the presence of the nitrate-reducing microbial aquifer community, which is in line with the observations of Redeker et al. (2014).

The findings imply that monoiodinated and deiodinated DTZ are more susceptible to microbial transformation in nitrate-reducing environments than the corresponding triiodinated compound and diiodinated derivative. Nevertheless, the persistence of TP 2 of DTZ–2I and DTZ–3I under nitrate-reducing conditions suggests that a subsequent shift in redox conditions may be essential to enable further transformation of these compounds. This underscores the conclusion drawn by Redeker et al. (2014) that complete mineralization of DTZ and its transformation products requires a succession of redox environments.

## Conclusion and environmental relevance

This study investigated how the degree of iodination influences the biodegradability and environmental behavior of ICM, specifically IOP, DTZ, and the ICM TP ATIA under aerobic and nitrate-reducing conditions.

ZWTs revealed immediate and rapid transformation of IOP derivatives, showing similar transformation pathways independent of the iodination degree. However, the detectability and stability of intermediate TPs were partially influenced by the number of iodine atoms. In addition, several TPs of IOP were identified beyond TP III.3 (DDPI), which had been previously reported as the final aerobic TP (Schulz et al. 2008). Based on these findings, the previously known aerobic transformation pathway was extended in this study by nine additional TPs, forming a fourth transformation phase.

In contrast, DTZ and ATIA derivatives displayed significant variability in biodegradation kinetics, strongly affected by the number and position of iodine substituents. The mineralization was significantly enhanced in the case of ATIA–2I B and ATIA–3I, demonstrated by substantial decrease of DOC compared to ATIA. These findings indicate that the influence of iodine atoms on transformation reactions decreases with increasing side-chain length, as in the case of the IOP derivative. When only functional groups remain bound directly to the aromatic ring, as seen in the ATIA derivatives, additional effects such as electron-withdrawing properties and the positional arrangement of iodine atoms become more relevant to biodegradation behavior.

In nitrate-reducing aquifer material–water tests, biodegradation proceeded much slower, accompanied by distinct adaptation periods. For IOP derivatives, aerobic transformation pathways also occurred under nitrate-reducing conditions and independent of the iodination degree, similar to the ZWT results. However, anaerobic TPs that were already present in the pore water and had undergone partial deiodination, exhibited additional oxidative transformations under nitrate-reducing conditions. This finding underscores the crucial influence of iodination degree in determining the diversity and extent of transformation pathways under changing redox conditions. The transformation of DTZ derivatives required at least two iodine atoms to be removed, determining whether a substance is accessible to microbial degradation under nitrate-reducing conditions. Thereby, the last iodine atom did not exert a significant influence on the degradation behavior of DTZ–2I compared to DTZ–3I.

These findings emphasize the environmental relevance of the reductive deiodination process, as it significantly enhances the biodegradability and potential mineralization of ICM and their TPs, particularly in contexts such as bank filtration and drinking water treatment. The persistence of iodinated aromatics presents challenges for their complete removal within water cycles, highlighting the necessity for specific monitoring and adaptive management strategies. Moreover, the study underscores the importance of understanding the biodegradability of TPs present in bank filtrates, especially when they re-enter oxic environments during drinking water treatment. This transition from anaerobic to aerobic conditions, typical for bank filtration followed by biological treatment, can enable further transformation and mineralization of iodinated aromatic compounds. Future risk assessments and management approaches must carefully consider how the iodination level affects both biodegradation kinetics and the complexity of transformation pathways under varying environmental conditions.

## Supplementary Information

Below is the link to the electronic supplementary material.

Supplementary file1 (PDF 1538 KB)

## Data Availability

No datasets were generated or analysed during the current study.

## Abbreviations

ATIA: 5-Amino-2,4,6-triiodoisophthalic acid
CV: Coefficient of variation
DABA: Desmethoxyacetyl iopromide
DAMI: 3,5-Diaminobenzoic acid
DDPI: Didespropanediol iopromide
DOC: Dissolved organic carbon
DT_50_: Degradation time 50
DTZ: Diatrizoate
HPLC–UV: High-performance liquid chromatography with UV detection
ICM: Iodinated contrast media
IOP: Iopromide
LC–ESI–MS: Liquid chromatography coupled to electrospray ionization mass spectrometry
LC–ESI–MS/MS: Liquid chromatography coupled to electrospray ionization tandem mass spectrometry
LOD: Limit of detection
LOQ: Limit of quantification
m/z: Mass-to-charge ratio
TiCi: Titanium(III) citrate
TP: Transformation product
ZWT: Zahn-Wellens test
