# Supplementary material for: Assessing the role of iodination degree on biodegradation kinetics and transformation pathways of iodinated contrast media and derivatives
Source: Biodegradation. 2025 Nov 23;37(1):1. doi: 10.1007/s10532-025-10213-6 (PMC12641039; doi:10.1007/s10532-025-10213-6)
Supplement: Supplementary file 1 — Supplementary file1 (PDF 1538 KB) [file 10532_2025_10213_MOESM1_ESM.pdf]

Appendices

Assessing the role of iodination degree on biodegradation kinetics and transformation pathways of iodinated contrast media and derivatives

Yuki Bartels[1], Martin Jekel[1] and Anke Putschew[1*]

[1]Technische Universität Berlin, Faculty III – Process Sciences, Institute of Environmental Technology, Department of Water Quality Control, Strasse des 17. Juni 135, 10623 Berlin, Germany

***Correspondence: anke.putschew@tu.berlin.de

Table of Contents

Appendix A Supporting Information for Materials and Methods 1

A.1 Chemicals, standards and solvents 1

A.2 LC-ESI-MS 3

A.3 HPLC-UV 7

Appendix B Results of Preparatory Studies and Monitoring Parameters 8

B.1 Nitrate-reducing aquifer material-water tests 8

B.2 Zahn-Wellens Tests 10

Appendix C Additional Results 14

C.1 Zahn-Wellens Tests 14

C.2 Nitrate-reducing aquifer material-water tests 38

Appendix A Supporting Information for Materials and Methods

A.1 Chemicals, standards and solvents

Iopromide (IOP) and diatrizoate (DTZ) were provided by Schering AG (Berlin, Germany). 5-amino-2,4,6-triiodoisophthalic acid (ATIA) was obtained from Merck KGaA (Darmstadt, Germany). The di-, mono-, and deiodinated derivatives of IOP, DTZ, and ATIA were produced via reductive deiodination of the respective triiodinated parent compounds following the procedures described by El-Athman et al. (2019a) and Stieber et al. (2011). The detailed production procedure is provided in Bartels et al. (2023).

All compounds were dissolved in ultrapure water produced using a Maxima Ultrapure Water System (ELGA, Upstadt-Weiher, Germany) with deionized feed water. Chemicals used for the preparation of the mineral medium followed the specifications of OECD Guideline 302B (OECD, 1992). Formaldehyde (37 wt% in H_2O, stabilized with 10–15% methanol) was used in sterile experiments. Methanol (J.T. Baker, USA) and formic acid (Merck KGaA, Darmstadt, Germany), both of HPLC grade, were employed for LC–MS analyses. Deuterated internal standards (iopromide-d_3 and diatrizoate-d_6) were purchased from Toronto Research Chemicals (Toronto, Canada) for compound quantification.

Table A.1: Concentrations of titanium(III) citrate and cyanocobalamin in the stock solutions and the corresponding target concentrations in the tests. For the IOP and DTZ single-compound tests, titanium(III) citrate and cyanocobalamin were additionally added at concentrations equivalent to those used in the IOP–3I and DTZ–3I tests, respectively. ATIA–3I for the single-compound tests was prepared according to the method of Stieber et al. (2011), without the addition of titanium(III) citrate and cyanocobalamin.

	**Substance**	**Concentration in the stock solution**	**Concentration in the test**
IOP single-compound test	Titanium(III) citrate	167 mM	228 µM
	Cyanocobalamin	5 g/L	0.46 µM
IOP–3I single-compound test	Titanium(III) citrate	5 mM	228 µM
	Cyanocobalamin	0.01 mM	0.46 µM
DTZ single-compound test	Titanium(III) citrate	167 mM	242 µM
	Cyanocobalamin	5 g/L	4.83 µM
DTZ–3I single-compound test	Titanium(III) citrate	5 mM	242 µM
	Cyanocobalamin	0.1 mM	4.84 µM
ATIA single-compound test	Titanium(III) citrate	-	-
	Cyanocobalamin	-	-
ATIA–3I single-compound test	Titanium(III) citrate	-	-
	Cyanocobalamin	-	-
IOP multi-compound test	Titanium(III) citrate	1.15 mM	111 µM
	Cyanocobalamin	0.01 mM	0.97 µM
DTZ multi-compound test	Titanium(III) citrate	1.25 mM	143 µM
	Cyanocobalamin	0.01 mM	1.15 µM
ATIA multi-compound test	Titanium(III) citrate	1.68 mM	195 µM
	Cyanocobalamin	0.03 mM	3.5 µM

A.2 LC-ESI-MS

For separation, a XSelect C18, 3.5 µm, 2.1×100 mm column was used (Waters, USA). As eluent, ultrapure water and methanol both acidified with formic acid (0.1 vol%) were used with an injection volume of 10 µL and a flow rate of 0.5 mL/min. For details concerning operational parameters, gradient elution, retention times and calibration see Tables A.2.1-A.2.3.

Triiodinated and deiodinated analytes were quantified using an external calibration, whereby the deiodinated compounds were produced in the lab using reductive deiodination (El-Athman et al., 2019a; Stieber et al., 2011). For the mono and diiodinated derivatives no certified standard or pure substance were available. For that reason, the concentrations were estimated on the basis of the consecutive first-order reaction with two partially deiodinated intermediate products using the quantified concentration of the triiodinated and deiodinated compound as well as the iodide concentration quantified by IC-UV (UV absorption at 226 nm) in the solution (El-Athman et al., 2019b).

Compounds for which no certified standards were available were semi-quantified based on the peak area of their corresponding m/z signals in the mass chromatogram. This applied to known aerobic ICM TPs described in the literature (Haiss and Kümmerer, 2006; Schulz et al., 2008), as well as to diiodinated, monoiodinated, and fully deiodinated ICM derivatives.

Potential TPs not previously proposed were hypothesized using the pathway prediction tool enviPath (Wicker et al., 2016) and the study by Helbling et al. (2010). The corresponding m/z values were calculated and screened for in the mass chromatograms. A signal was considered a confirmed TP if it was detected in all triplicates within a similar sampling time range and showed a signal-to-noise ratio of at least 3.

Table A.2.1: LC-ESI-MS operational parameters.

	**Selective detection of organic bound iodine**	**Detection of molecular ions**
Ionization mode	negative-ESI	positive-ESI
Detection mode	selective ion monitoring mode	scan mode
Mass detection (m/z)	127	80–900
Capillary voltage (kV)	2.3	3.0
Cone voltage (V)	50	25
Source temperature (°C)	120	120
Desolvation temperature (°C)	250	280
Desolvation gas	N_2	N_2
Desolvation gas flow (L/h)	400	400
Nebulizer gas	N_2	N_2
Nebulizer gas flow (L/h)	35	35

Table A.2.2: Gradient elution in LC-ESI-MS. *acidified with 0.1% formic acid.

**Time (min)**	**Water* (%)**	**Methanol* (%)**
0.0	95	5
0.5	95	5
10.0	90	10
19.0	20	80
20.0	20	80
20.5	95	5
26.0	95	5

Table A.2.3: Molar mass, retention time (t_R), and calibration range of the initial compounds.

Compound	Molar mass (g/mol)	t_R (min)	Calibration range (mg/L)
IOP	791.12	7.3	0.1–20.0
IOP–1I	665.22	13.5	-
IOP–2I	539.32	16.7	-
IOP–3I	413.43	10.9	0.1–20.0
DTZ	613.91	2.0	0.1–20.0
DTZ–1I	488.01	2.0	-
DTZ–2I	363.00	7.9	-
DTZ–3I	236.21	15.3	0.1–20.0
ATIA	558.84	1.7	0.5–25.0
ATIA–1I	432.94	1.9	-
ATIA–2I A	307.04	1.8	-
ATIA–2I B	307.00	10.7	
ATIA–3I	413.43	4.2	0.5–20.0

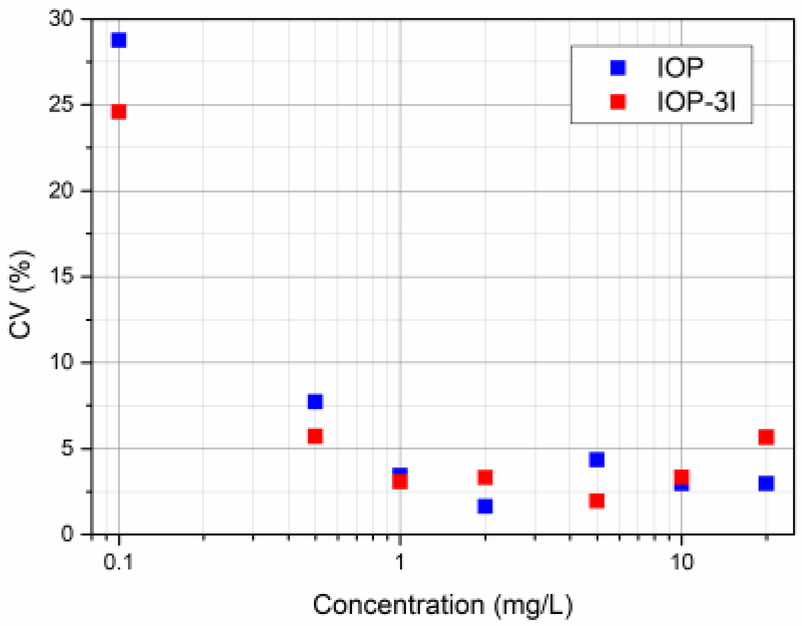

Fig. A.2.1: Variation coefficient (CV) of the MS signal areas over different target concentrations for IOP and IOP–3I; CV is determined using mean and standard deviation of n = 10 external calibration measurements.

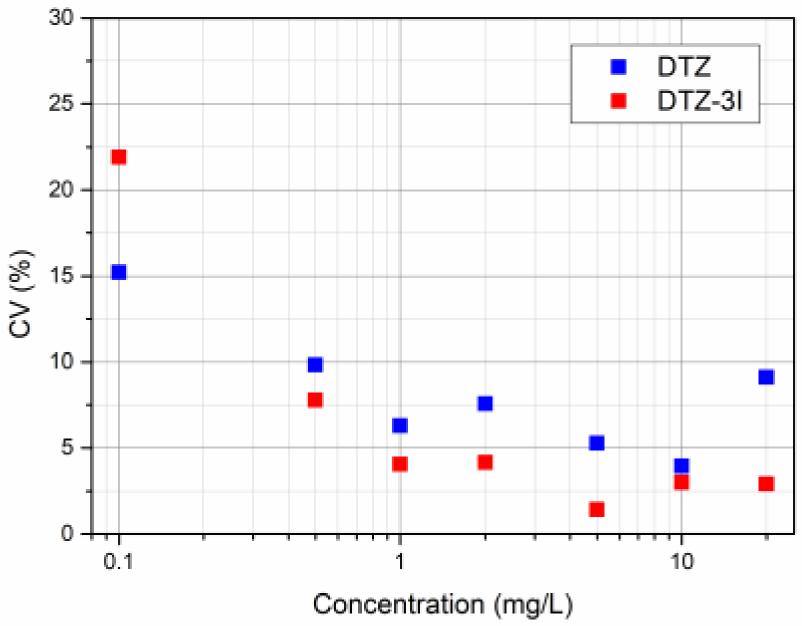

Fig. A.2.2: Variation coefficient (CV) of the MS signal areas over different target concentrations for DTZ and DTZ–3I; CV is determined using mean and standard deviation of n = 10 external calibration measurements.

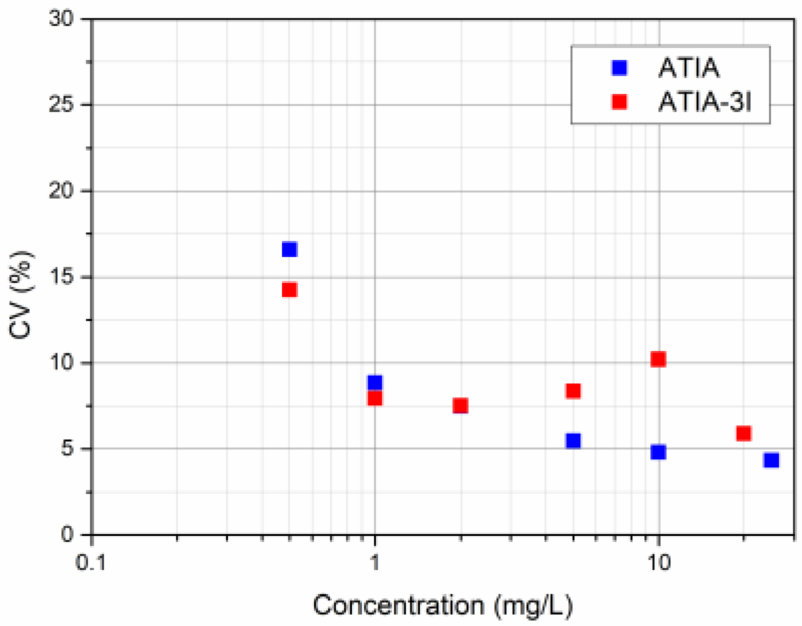

Fig. A.2.3: Variation coefficient (CV) of the MS signal areas over different target concentrations for ATIA and ATIA–3I; CV is determined using mean and standard deviation of n = 27 (ATIA) and n = 20 (ATIA–3I) external calibration measurements.

A.3 HPLC-UV

An HPLC-UV system (Agilent 1200, Waldbronn, Germany) was used for the quantification of the reference compound aniline. The lowest calibration concentration was 0.5 mg/L with a signal-to-noise ratio higher than 3. For separation, a reversed phase column was used (Phenomenex Luna 3 mm C18(2), 150×2 mm) using a column temperature of 30 °C and a flow rate of 0.25 mL/min. The injection volume was 10 µL. The mobile phase consisted of water and methanol, both acidified with 0.05% trifluoroacetic acid. Aniline was detected at a wavelength of 254 nm.

Table A.3: Gradient elution in HPLC-UV. * acidified with 0.05% trifluoroacetic acid.

Time (min)	Water* (%)	Methanol* (%)
0.0	95	5
5.0	90	10
6.0	50	50
6.2	95	5
15.0	95	5

Appendix B Results of Preparatory Studies and Monitoring Parameters

B.1 Nitrate-reducing aquifer material-water tests

Table B.1.1: Concentrations (mean and standard deviation) of DOC and redox potential indicating ions measured in the river water (n = 17) and in the bank filtrate (pore water) at sampling point P1 and P2 (using piezometers, n = 4). Concentrations are given in mg/L. All data were obtained from Schaper et al. (2025).

Sampling point	DOC	NH_4^+-N	NO_3^--N	SO_4^{2-}	Mn^{2+}	Fe^{2+}
River water	11.83 ± 1.02	0.4 ± 0.5	6.4 ± 0.7	137.2 ± 16.1	0.11 ± 0.03	0.04 ± 0.01
P1	8.31 ± 0.37	0.6 ± 0.4	0.0 ± 0.0	141.0 ± 23.4	0.46 ± 0.10	4.29 ± 0.60
P2	8.28 ± 0.52	0.6 ± 0.3	0.0 ± 0.0	156.7 ± 6.4	0.38 ± 0.04	3.50 ± 0.36

Table B.1.2: pH (in 0.01 M $CaCl_2$ solution), sand content, organic content and fraction of organic carbon (f_{OC}) determined in the aquifer materials S1 and S2.

Material	Sampling Point	Distance from riverbank (m)	pH	Sand content (%)	Organic content (%)	f_{OC} (%)
S1	P1	6.9	7.38	> 99	0.3	0.07
S2	P2	10.4	7.50	> 99	0.1	0.06

Table B.1.3: Concentrations of redox potential indicating ions measured in the suspensions of the nitrate-reducing aquifer material-water tests. * day of sodium nitrate dosing.

Aquifer material	Compound mix	Sampling day	Duplicate a				Duplicate b				Sterile test			
			NO_3^-	Mn^{2+}	Fe^{2+}	SO_4^{2-}	NO_3^-	Mn^{2+}	Fe^{2+}	SO_4^{2-}	NO_3^-	Mn^{2+}	Fe^{2+}	SO_4^{2-}
S1	IOP mix	–9	20.3	<0.01	0.47	15.9	21.1	<0.01	0.38	16.7	21.3	<0.01	0.30	15.9
		40	8.6	<0.01	0.42	17.6	7.1	<0.01	0.28	18.3	13.5	<0.01	0.40	16.0
		54	6.9	–	–	17.6	3.8	–	–	18.3	13.3	–	–	15.2
		57*	31.9	–	–	16.8	26.5	–	–	17.1	–	–	–	–
		68	28.1	–	–	17.5	22.7	–	–	18.1	13.3	–	–	15.2
		89	23.9	<0.01	0.65	17.7	16.9	<0.01	0.53	18.5	13.7	<0.01	0.51	16.7
		117	15.3	–	–	16.9	11.1	–	–	17.6	–	–	–	–
	DTZ mix	–9	19.6	<0.01	0.51	15.9	19.2	<0.01	0.39	16.1	19.4	<0.01	0.48	16.4
		40	5.4	<0.01	0.47	18.2	4.7	<0.01	0.52	18.3	12.3	<0.01	0.38	16.5
		54	3.5	–	–	18.2	2.4	–	–	18.2	12.0	–	–	16.2
		57*	28.0	–	–	17.5	26.9	–	–	17.1	–	–	–	–
		68	25.3	–	–	18.0	24.7	–	–	18.2	11.8	–	–	16.0
		89	24.7	<0.01	0.58	18.4	23.6	<0.01	0.52	18.5	12.1	<0.01	0.47	17.1
		117	21.3	–	–	17.5	20.0	–	–	18.0	–	–	–	–
S2	IOP mix	–9	21.8	<0.01	0.42	11.4	22.6	<0.01	0.36	12.0	22.8	<0.01	0.29	11.3
		40	17.0	<0.01	0.34	12.5	16.9	<0.01	0.43	12.6	19.9	<0.01	0.44	10.7
		68	4.0	–	–	12.8	4.2	–	–	12.5	19.7	–	–	10.5
		70*	28.4	–	–	11.6	28.5	–	–	11.7	19.8	–	–	10.2
		89	28.7	<0.01	0.58	11.9	28.0	<0.01	0.42	12.1	–	<0.01	0.38	–
		117	27.7	–	–	11.2	26.3	–	–	11.3	–	–	–	–
	DTZ mix	–9	21.6	<0.01	0.35	12.2	20.5	<0.01	0.25	11.1	21.1	<0.01	0.35	11.9
		40	17.7	<0.01	0.55	13.4	17.4	<0.01	0.32	13.0	18.6	<0.01	0.57	12.1
		68	16.8	–	–	13.2	16.2	–	–	12.8	18.1	–	–	11.8
		89	16.4	<0.01	0.45	12.9	15.6	<0.01	0.45	12.7	18.8	<0.01	0.48	11.8
		117	15.0	–	–	12.2	14.0	–	–	11.9	–	–	–	–

B.2 Zahn-Wellens Tests

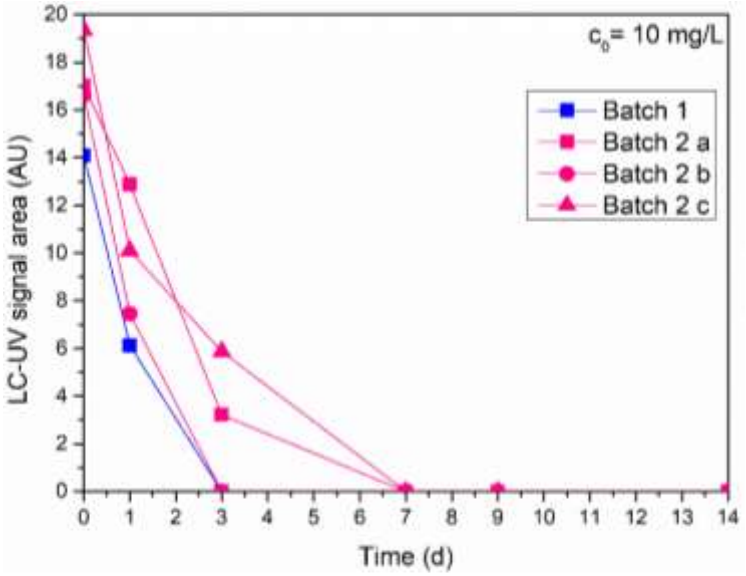

Fig. B.2.1: Concentration (plotted as HPLC-UV signal area) of aniline during the course of the ZWTs using sludge from **batch 1 and 2**.

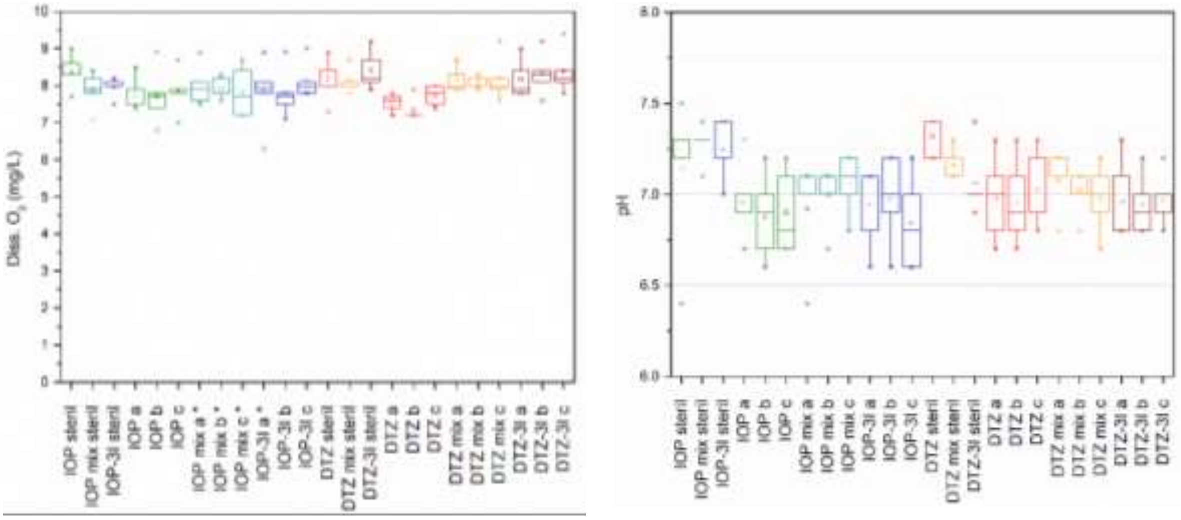

Fig. B.2.2: Concentration of dissolved oxygen (left) and pH (right) in the suspensions of the ZWTs using sludge from **batch 1**, measured over a period of 200 d; n = 5, * n = 6.

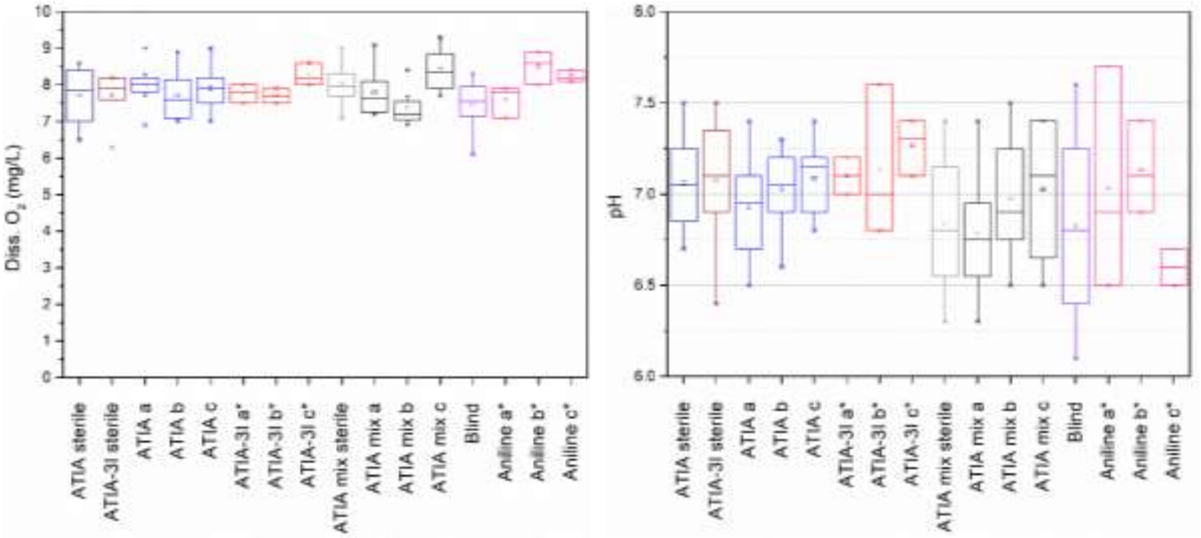

Fig. B.2.3: Concentration of dissolved oxygen (left) and pH (right) in the suspensions of the ZWT using sludge from **batch 2**, measured over a period of 217 d; n = 8, * n = 3.

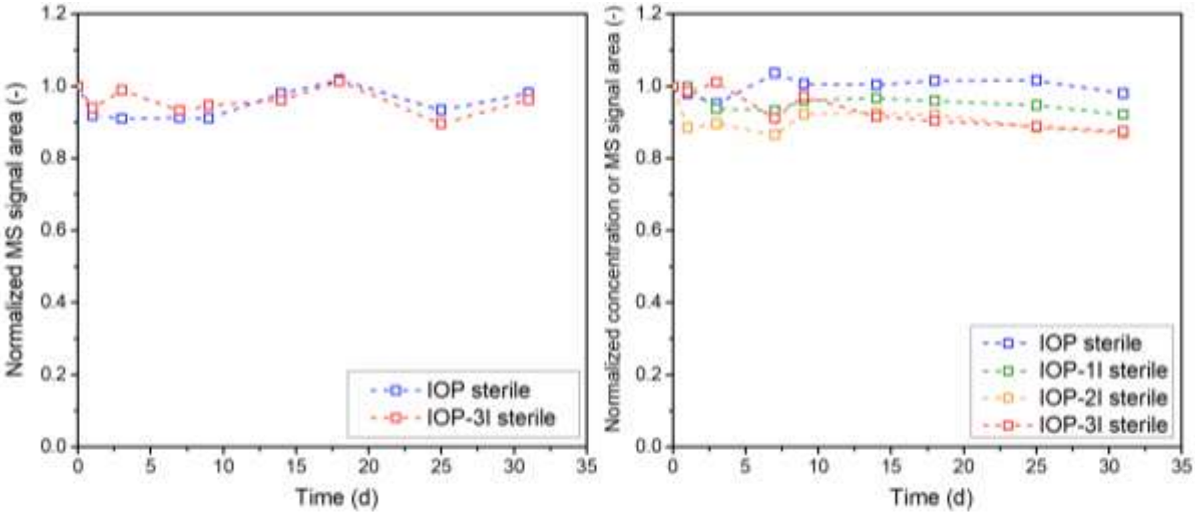

Fig. B.2.4: Normalized concentration (IOP, IOP–3I) or MS signal area (IOP–1I, IOP–2I) of the sterile controls determined in the single- (left) and multi-compound (right) ZWTs. Mean concentrations over time: IOP 11.6 ± 0.5 mg/L, IOP–3I 8.0 ± 0.3 mg/L (left); IOP 2.9 ± 0.2 mg/L, IOP–3I 0.4 ± 0.0 mg/L (right).

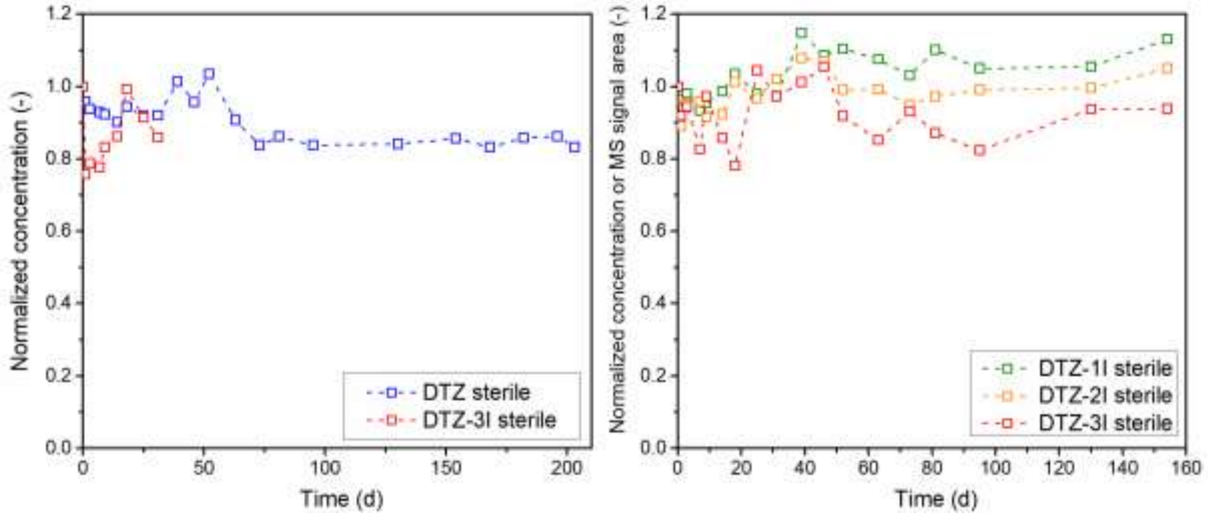

Fig. B.2.5: Normalized concentration (DTZ, DTZ–3I) or MS signal area (DTZ–1I, DTZ–2I) of the sterile controls determined in the single- (left) and multi-compound (right) ZWTs. Mean concentrations over time: DTZ 8.9 ± 0.6 mg/L, DTZ–3I 5.0 ± 0.5 mg/L (left); DTZ–3I 1.2 ± 0.1 mg/L (right).

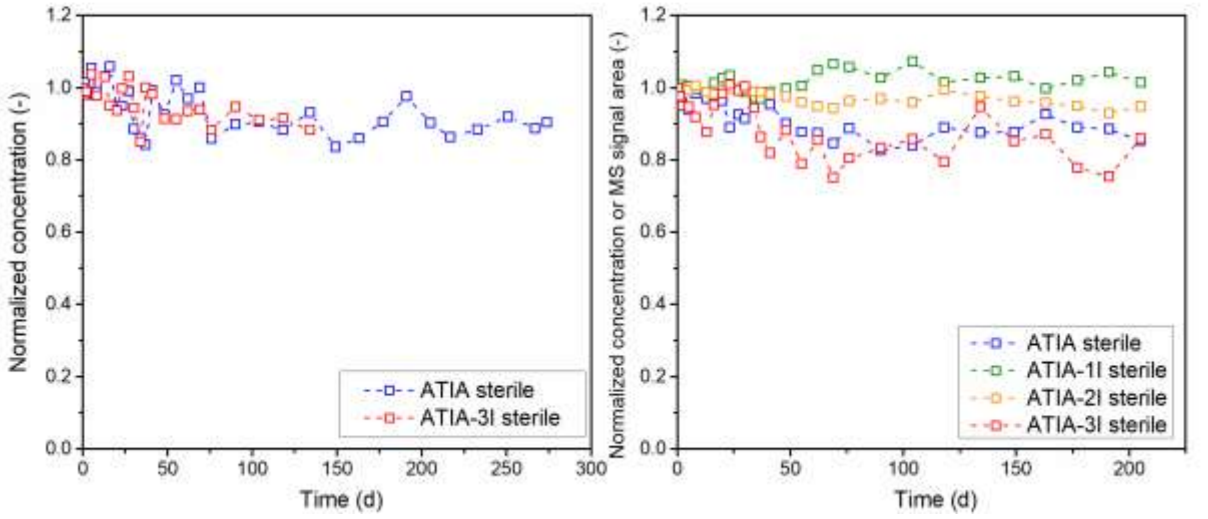

Fig. B.2.6.: Normalized concentration (ATIA, ATIA–3I) or MS signal area (ATIA–1I, ATIA–2I) of the sterile controls determined in the single- (left) and multi-compound (right) ZWT; mean concentrations over time: ATIA 53.3 ± 3.6 mg/L, ATIA–3I 19.2 ± 1.0 mg/L (left); ATIA 8.1 ± 0.5 mg/L, ATIA–3I 4.1 ± 0.4 mg/L (right).

Table B.2.1: Difference in concentration (mg/L) or MS signal area (AU) between the test suspension and sterile control at t_0 (3 h) to assess initial sorption to activated sludge in ZWT. Sorption was considered valid if the mean deviation exceeded the calibration CV (triiodinated and deiodinated) or 10% (monoiodinated and diiodinated).

Test	Compound	Difference	Deviation from sterile control	Mean deviation from sterile control	Assessment	Sorption?
Single-compound test	IOP a	–0.13 mg/L	–1.0%	–0.7 ± 1.9%	Individual deviations lower than calib. CV at 0.1 mg/L; negative deviations.	No
	IOP b	–0.30 mg/L	–2.4%			
	IOP c	0.17 mg/L	1.4%			
	IOP–3I a	1.30 mg/L	15.3%	16.4 ± 2.2%	Deviation significantly higher than calib. CV at 1 mg/L.	Yes
	IOP–3I b	1.29 mg/L	15.1%			
	IOP–3I c	1.62 mg/L	18.9%			
Multi-compound test	IOP a	0.17 mg/L	5.5%	0.5 ± 4.6%	Individual deviations are ambiguous; deviation lower than calib. CV at 0.1 mg/L; negative deviations.	No
	IOP b	–0.02 mg/L	–0.6%			
	IOP c	–0.11 mg/L	–3.6%			
	IOP–1I a	–735 AU	–0.6%	0.7 ± 1.1%	Individual deviations low; negative deviation.	No
	IOP–1I b	1666 AU	1.4%			
	IOP–1I c	1609 AU	1.3%			
	IOP–2I a	–820 AU	–0.7%	–0.8 ± 1.9%	Individual deviations low; negative deviations.	No
	IOP–2I b	1331 AU	1.1%			
	IOP–2I c	–3132 AU	–2.7%			
	IOP–3I a	Concentration differences < LOD				
	IOP–3I b					
	IOP–3I c					
Single-compound test	DTZ a	–0.42 mg/L	–4.3%	–1.1 ± 3.1%	Negative deviations; Deviation lower than calib. CV at 0.1 mg/L.	No
	DTZ b	–0.09 mg/L	–1.0%			
	DTZ c	0.19 mg/L	2.0%			
	DTZ–3I a	0.32 mg/L	5.6%	5.2 ± 0.5%	All individual values show similar tendency; However, still uncertain as deviations are lower than calib. CV at 0.1 and 0.5 mg/L.	Not significant
	DTZ–3I b	0.27 mg/L	4.7%			
	DTZ–3I c	0.30 mg/L	5.2%			
Multi-compound test	DTZ a	–	–	–	-	–
	DTZ b	–	–			
	DTZ c	–	–			
	DTZ–1I a	3686 AU	3.1%	3.7 ± 0.5%	All individual values show similar tendency, but low deviations and missing external calibration data.	Not significant
	DTZ–1I b	4560 AU	3.9%			
	DTZ–1I c	4836 AU	4.1%			
	DTZ–2I a	2763 AU	2.1%	2.4 ± 1.4%	All individual values show similar tendency, but low deviations and missing external calibration data	Not significant
	DTZ–2I b	1598 AU	1.2%			
	DTZ–2I c	5126 AU	4.0%			
	DTZ–3I a	0.05 mg/L	4.0%	6.0 ± 4.7%	Not all individual values show similar tendency; mean deviation is lower than calib. CV at 0.1 mg/L.	Not significant
	DTZ–3I b	0.03 mg/L	2.6%			
	DTZ–3I c	0.14 mg/L	11.3%			
Single-compound test	ATIA a	0.43 mg/L	0.7%	–1.1 ± 4.6%	Individual deviations are ambiguous; deviation lower than calib. CV at 1 mg/L; negative deviation.	No
	ATIA b	–3.62 mg/L	–6.4%			
	ATIA c	1.28 mg/L	2.2%			
	ATIA–3I a	1.55 mg/L	7.7%	9.5 ± 1.6%	All individual values show similar tendency; Deviations higher than calib. CV at 1 and 2 mg/L.	Yes
	ATIA–3I b	2.16 mg/L	10.7%			
	ATIA–3I c	2.01 mg/L	10.0%			
Multi-compound test	ATIA a	0.82 mg/L	9.3%	4.6 ± 4.1%	All individual values show similar tendency, but deviations are lower than calib. CV at 0.5 and 1 mg/L.	No
	ATIA b	0.17 mg/L	1.9%			
	ATIA c	0.22 mg/L	2.4%			
	ATIA–1I a	19743 AU	15.2%	16.2 ± 1.8%	All individual values show similar tendency; deviations higher than 10%.	Yes
	ATIA–1I b	23864 AU	18.3%			
	ATIA–1I c	19776 AU	15.2%			
	ATIA–2I A a	49116 AU	28.5%	18.2 ± 8.9%	All individual values show similar tendency; deviations higher than 10%.	Yes
	ATIA–2I A b	22447 AU	13.0%			
	ATIA–2I A c	22448 AU	13.0%			
	ATIA–2I B a	–2249 AU	–1.3%	0.0 ± 1.6%	Individual deviations low; negative deviations.	No
	ATIA–2I B b	3014 AU	1.8%			
	ATIA–2I B c	–778 AU	–0.5%			
	ATIA–3I a	0.40 mg/L	8.6%	6.0 ± 2.5%	All individual values show similar tendency, but deviations are lower than calib. CV at 0.5 mg/L.	Not significant
	ATIA–3I b	0.50 mg/L	5.6%			
	ATIA–3I c	0.32 mg/L	3.6%			

Appendix C Additional Results

C.1 Zahn-Wellens Tests

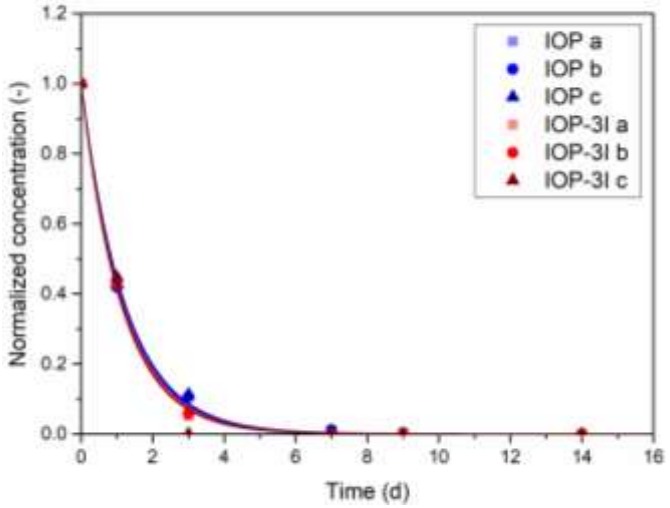

Fig. C.1.1: Fitting of the (normalized) concentration decrease of IOP and IOP–3I during the course of the single-compound ZWTs conducted in triplicates (a, b and c) using a first order reaction kinetic model (exponential decay).

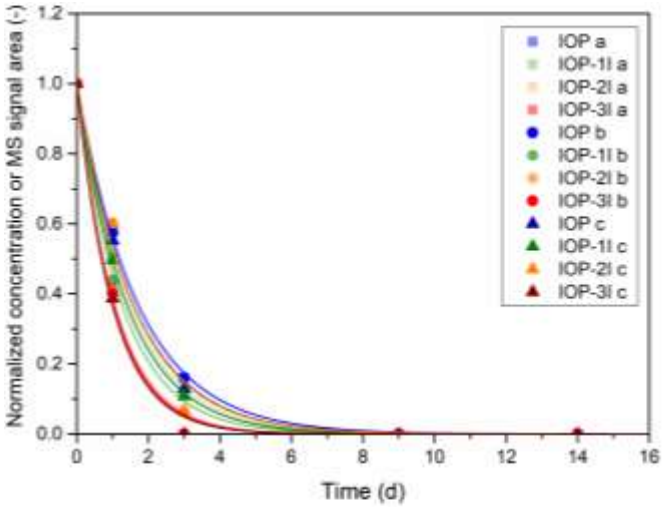

Fig. C.1.2: Fitting of the (normalized) concentration decrease of IOP, IOP–1I and IOP–2I during the course of the multi-compound ZWTs conducted in triplicates (a, b and c) using a first order reaction kinetic model (exponential decay).

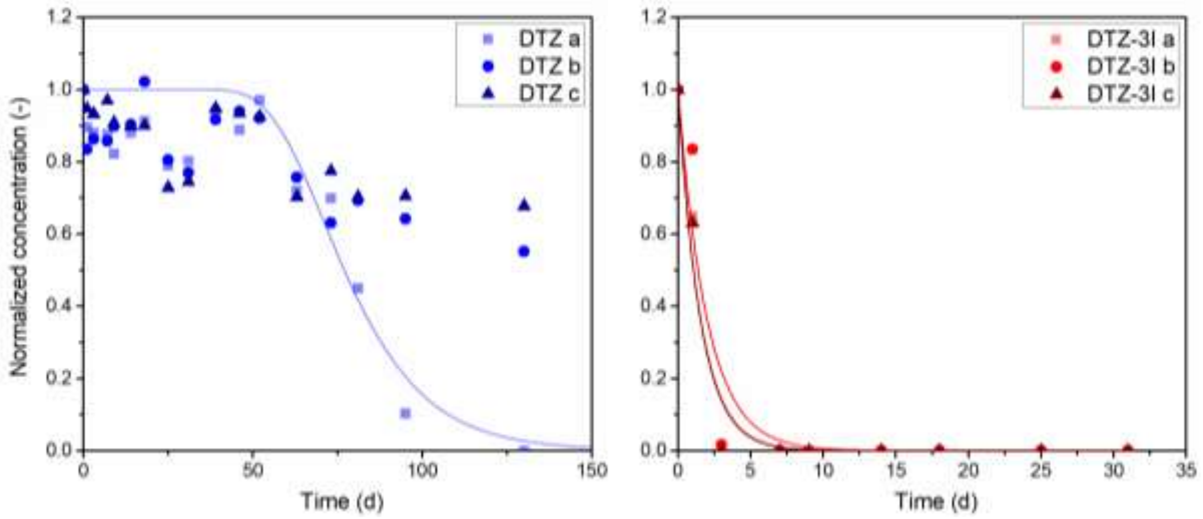

Fig. C.1.3: Fitting of the (normalized) concentration decrease of DTZ (left) and DTZ–3I (right) during the course of the single-compound ZWTs conducted in triplicates (a, b and c). A modified Gompertz model was used for DTZ and a first order reaction kinetic model (exponential decay) was used for DTZ–3I.

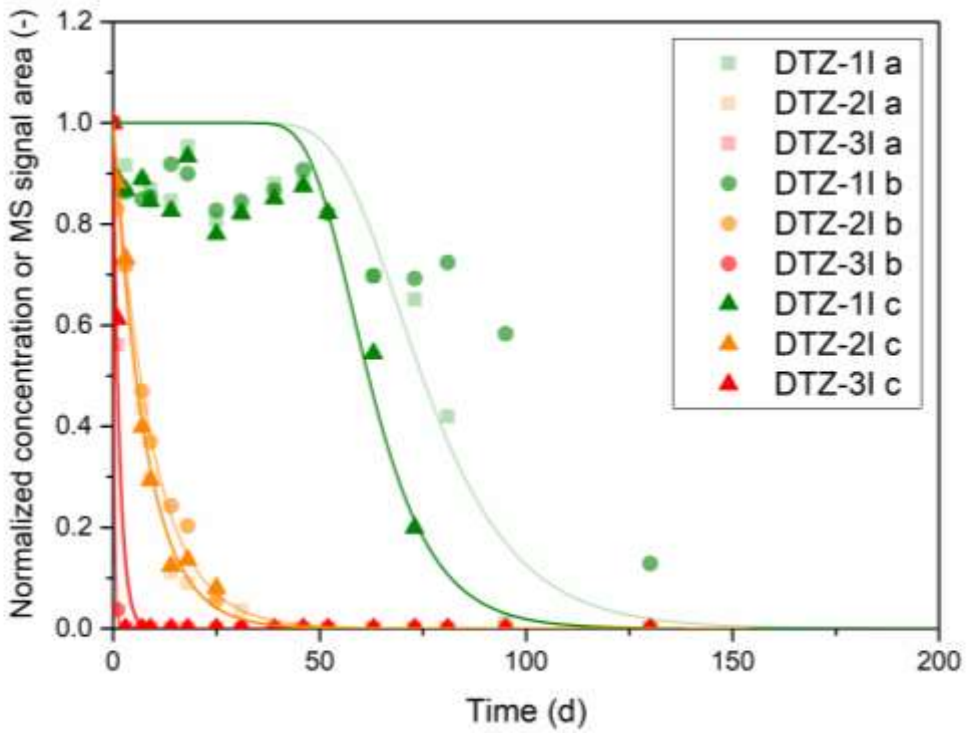

Fig. C.1.4: Fitting of the (normalized) concentration decrease of DTZ–1I, DTZ–2I and DTZ–3I during the course of the multi-compound ZWTs conducted in triplicates (a, b and c). A modified Gompertz model was used for DTZ–1I and DTZ–2I and a first order reaction kinetic (exponential decay) was used for DTZ–3I.

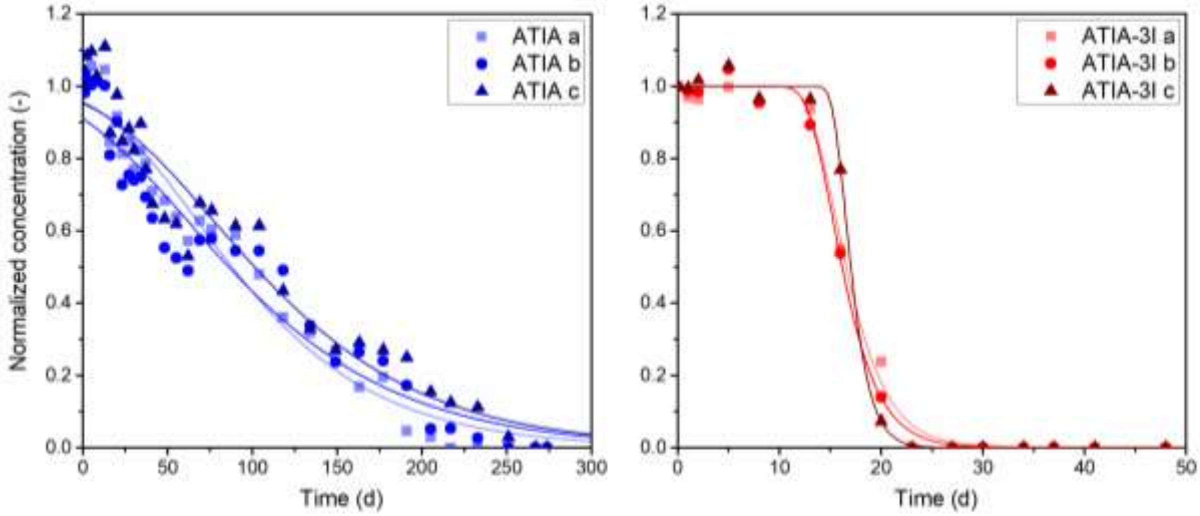

Fig. C.1.5: Fitting of the (normalized) concentration decrease of ATIA (left) and ATIA–3I (right) during the course of the single-compound ZWTs conducted in triplicates (a, b and c). A modified Gompertz model was used for fitting.

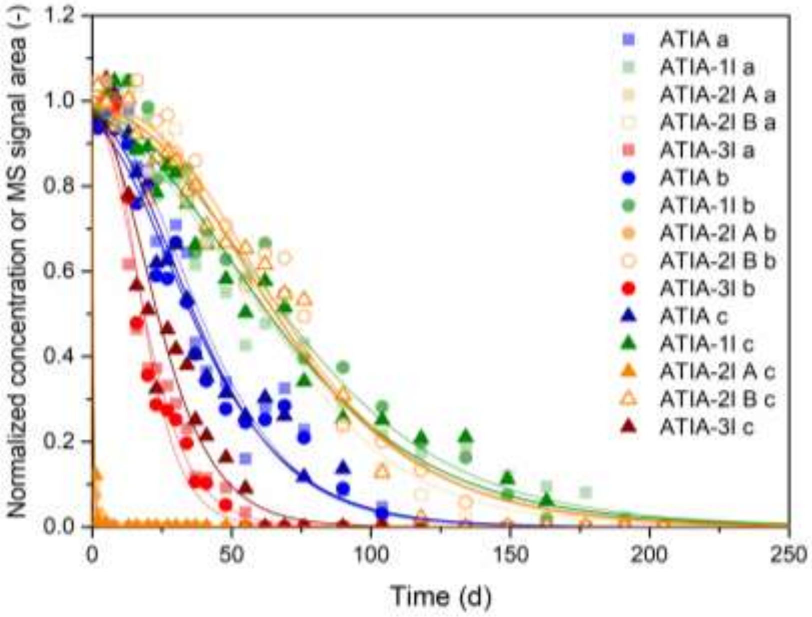

Fig. C.1.6: Fitting of the (normalized) concentration decrease of ATIA, ATIA–1I, ATIA–2I and ATIA–3I during the course of the multi-compound ZWTs conducted in triplicates (a, b and c). A modified Gompertz model was used for ATIA, ATIA–1I, and ATIA–3I and a first order reaction kinetic (exponential decay) was used for ATIA–2I.

Table C.1.1: DT_{50} values for IOP, DTZ and ATIA as well as their mono (–2I), di (–1I) and deiodinated (–3I) derivatives determined in ZWT.

Test	Compound	-k (1/d)	DT_{50} (d)	L (d)	λ (d)	c_{start} (fixed)	c_{end}(fixed)	R^2	Model
Single-compound	DTZ a	0.06	65.0	71.1	54.7	1.00	0.00	0.82	Mod. Gompertz
	DTZ b	-	-	-	-	-	-	-	-
	DTZ c	-	-	-	-	-	-	-	-
	DTZ–3I a	0.64	1.08	-	-	1.00	-	0.97	1st Order Decay
	DTZ–3I b	0.51	1.37	-	-	1.00	-	0.93	1st Order Decay
	DTZ–3I c	0.66	1.05	-	-	1.00	-	0.97	1st Order Decay
Multi-compound	DTZ–1I a	0.06	64.7	69.2	53.3	1.00	0.00	0.87	Mod. Gompertz
	DTZ–1I b	-	-	-	-	-	-	-	-
	DTZ–1I c	0.09	54.0	58.0	47.1	1.00	0.00	0.90	Mod. Gompertz
	DTZ–2I a	0.13	5.37	-	-	1.00	-	1.00	1st Order Decay
	DTZ–2I b	0.11	6.48	-	-	1.00	-	0.99	1st Order Decay
	DTZ–2I c	0.13	5.42	-	-	1.00	-	1.00	1st Order Decay
	DTZ–3I a	0.73	0.95	-	-	1.00	-	0.98	1st Order Decay
	DTZ–3I b	1.03	0.67	-	-	1.00	-	1.00	1st Order Decay
	DTZ–3I c	0.68	1.03	-	-	1.00	-	0.98	1st Order Decay
Single-compound	IOP a	0.81	0.86	-	-	1.00	-	1.00	1st Order Decay
	IOP b	0.84	0.83	-	-	1.00	-	1.00	1st Order Decay
	IOP c	0.82	0.85	-	-	1.00	-	1.00	1st Order Decay
	IOP–3I a	0.85	0.81	-	-	1.00	-	1.00	1st Order Decay
	IOP–3I b	0.87	0.79	-	-	1.00	-	1.00	1st Order Decay
	IOP–3I c	0.89	0.78	-	-	1.00	-	0.99	1st Order Decay
Multi-compound	IOP a	0.58	1.19	-	-	1.00	-	1.00	1st Order Decay
	IOP b	0.58	1.19	-	-	1.00	-	1.00	1st Order Decay
	IOP c	0.63	1.10	-	-	1.00	-	1.00	1st Order Decay
	IOP–1I a	0.68	1.02	-	-	1.00	-	0.99	1st Order Decay
	IOP–1I b	0.79	0.88	-	-	1.00	-	0.99	1st Order Decay
	IOP–1I c	0.71	0.97	-	-	1.00	-	0.99	1st Order Decay
	IOP–2I a	0.63	1.11	-	-	1.00	-	0.99	1st Order Decay
	IOP–2I b	0.64	1.09	-	-	1.00	-	0.99	1st Order Decay
	IOP–2I c	0.64	1.09	-	-	1.00	-	0.99	1st Order Decay
	IOP–3I a	0.94	0.74	-	-	1.00	-	1.00	1st Order Decay
	IOP–3I b	0.97	0.71	-	-	1.00	-	1.00	1st Order Decay
	IOP–3I c	1.00	0.69	-	-	1.00	-	1.00	1st Order Decay
Single-compound	ATIA a	0.02	45.2	66.7	7.89	1.00	0.00	0.97	Mod. Gompertz
	ATIA b	0.01	34.7	60.9	10.5	1.00	0.00	0.93	Mod. Gompertz
	ATIA c	0.02	52.0	76.5	9.80	1.00	0.00	0.94	Mod. Gompertz
	ATIA–3I a	0.37	14.6	15.6	12.8	1.00	0.00	1.00	Mod. Gompertz
	ATIA–3I b	0.40	14.3	15.2	12.7	1.00	0.00	1.00	Mod. Gompertz
	ATIA–3I c	0.75	16.0	16.5	15.2	1.00	0.00	1.00	Mod. Gompertz
Multi-compound	ATIA a	0.05	22.6	30.6	8.85	1.00	0.00	0.97	Mod. Gompertz
	ATIA b	0.04	18.4	26.9	3.62	1.00	0.00	0.98	Mod. Gompertz
	ATIA c	0.05	20.3	28.4	6.18	1.00	0.00	0.98	Mod. Gompertz
	ATIA–1I a	0.03	32.4	47.1	7.05	1.00	0.00	0.97	Mod. Gompertz
	ATIA–1I b	0.03	39.4	54.1	14.1	1.00	0.00	0.98	Mod. Gompertz
	ATIA–1I c	0.03	34.9	49.0	10.5	1.00	0.00	0.98	Mod. Gompertz
	ATIA–2I A a	2.30	0.30	-	-	1.00	-	1.00	First Order Decay
	ATIA–2I A b	2.56	0.27	-	-	1.00	-	1.00	First Order Decay
	ATIA–2I A c	2.10	0.33	-	-	1.00	-	1.00	First Order Decay
	ATIA–2I B a	0.03	40.9	52.0	21.7	1.00	0.00	0.98	Mod. Gompertz
	ATIA–2I B b	0.03	43.6	55.8	22.5	1.00	0.00	0.98	Mod. Gompertz
	ATIA–2I B c	0.03	40.5	53.1	18.6	1.00	0.00	0.97	Mod. Gompertz
	ATIA–3I a	0.09	10.8	14.8	3.89	1.00	0.00	0.98	Mod. Gompertz
	ATIA–3I b	0.11	12.0	15.2	6.43	1.00	0.00	0.98	Mod. Gompertz
	ATIA–3I c	0.07	13.4	18.4	4.69	1.00	0.00	0.97	Mod. Gompertz

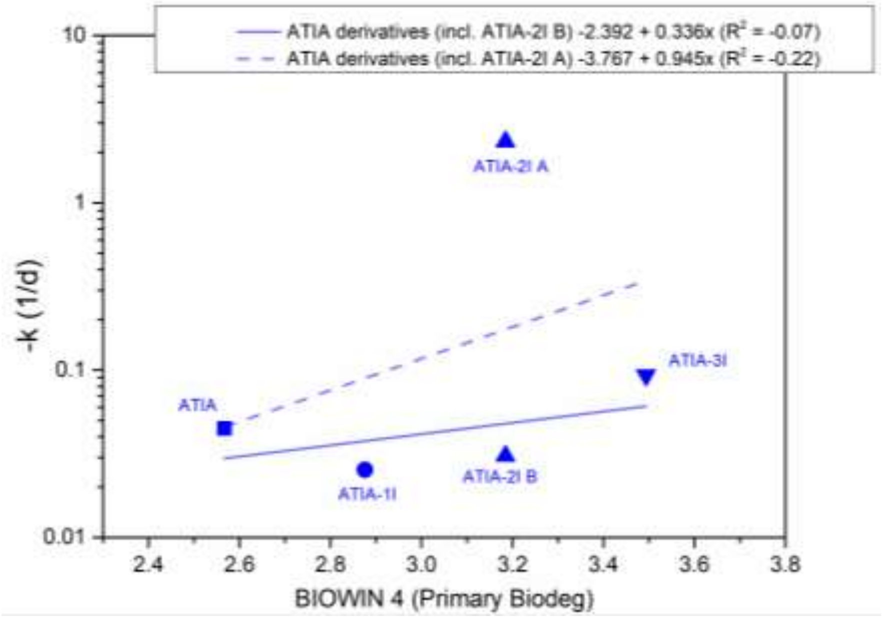

Fig. C.1.7: Comparison of the experimentally determined biodegradation rate constants (k) with the theoretical BIOWIN4 values (primary biodegradation). BIOWIN4 values were estimated using EPI Suite™ v4.11 (U.S. Environmental Protection Agency, 2012).

BIOWIN4 values means primary degradation in:
5–4.75: hours
4.75–4.25: hours to days
4.25–3.75: days
3.75–3.25: days to weeks
3.25–2.75: weeks
2.75–2.25: weeks to months
2.25–1.75: in months
<1.75: refractory

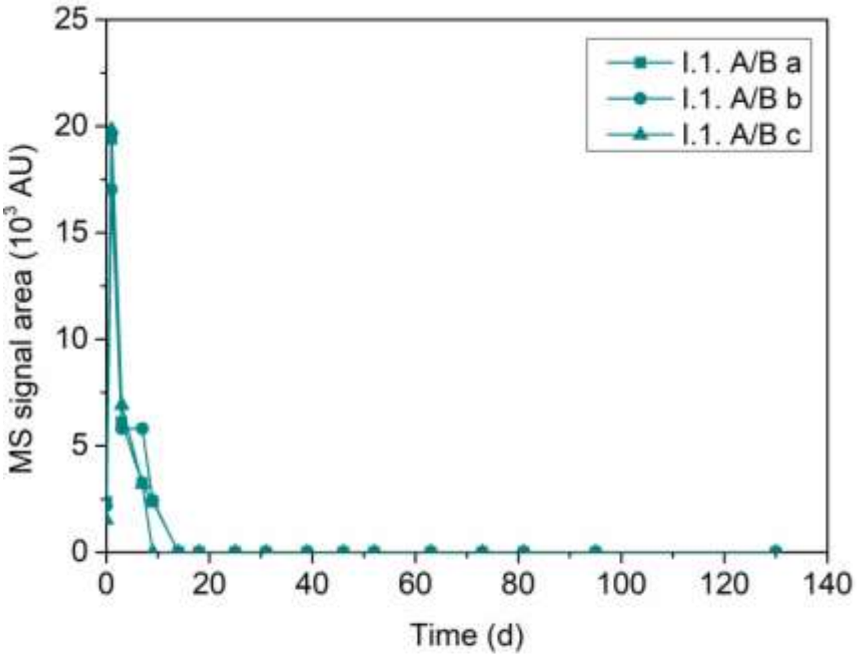

Fig. C.1.8: Concentration of the first phase TP I.1 A/B (805) of **IOP** during the course of the single-compound ZWT conducted in triplicates (a, b and c). Concentration in MS signal area unit (AU).

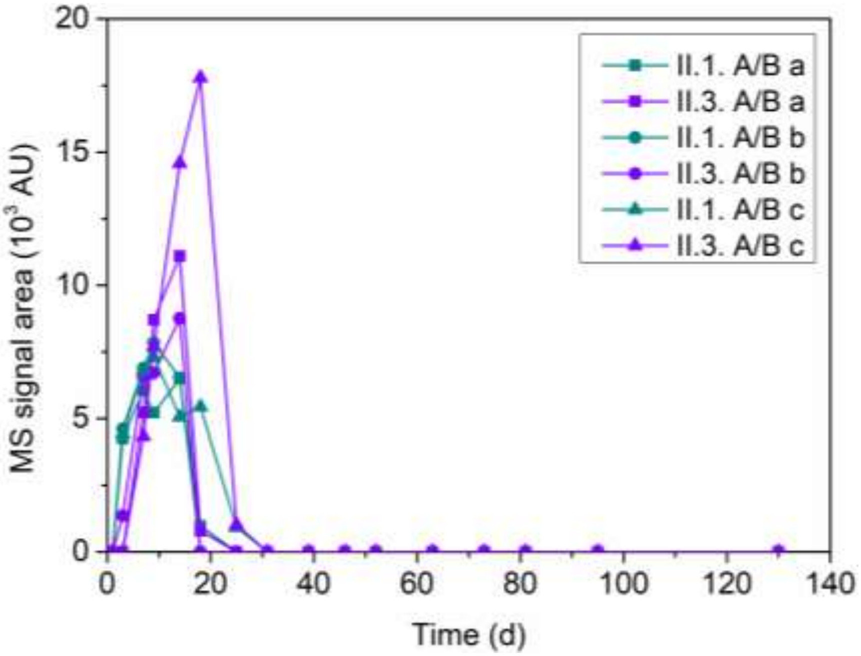

Fig. C.1.9: Concentration of the second phase TP II.1 A/B (817) and II.3 A/B (729) of **IOP** during the course of the single-compound ZWT conducted in triplicates (a, b and c). Concentration in MS signal area unit (AU).

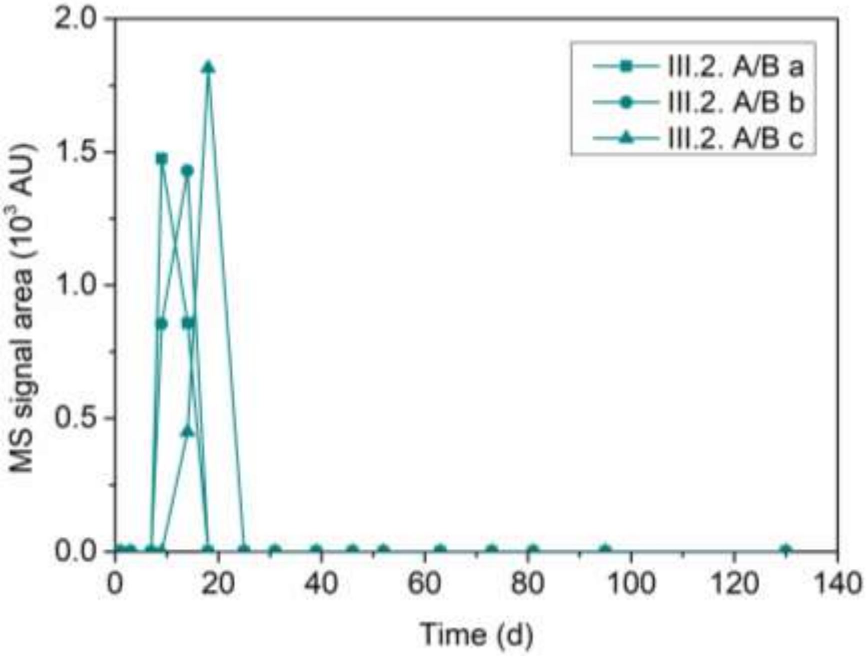

Fig. C.1.10: Concentration of the third phase TP III.2 A/B (701) of **IOP** during the course of the single-compound ZWT conducted in triplicates (a, b and c). Concentration in MS signal area unit (AU).

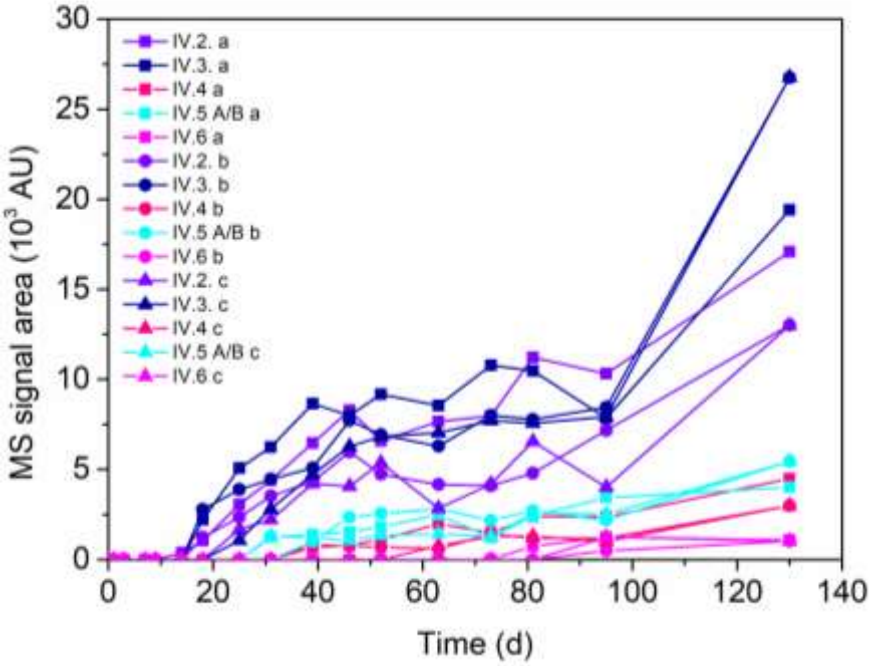

Fig. C.1.11: Concentration of the fourth phase TP IV.2 (571), IV.3 (629), IV.4 (572), IV.5 A/B (630) and IV.6 (557) of **IOP** during the course of the single-compound ZWT conducted in triplicates (a, b and c). Concentration in MS signal area unit (AU).

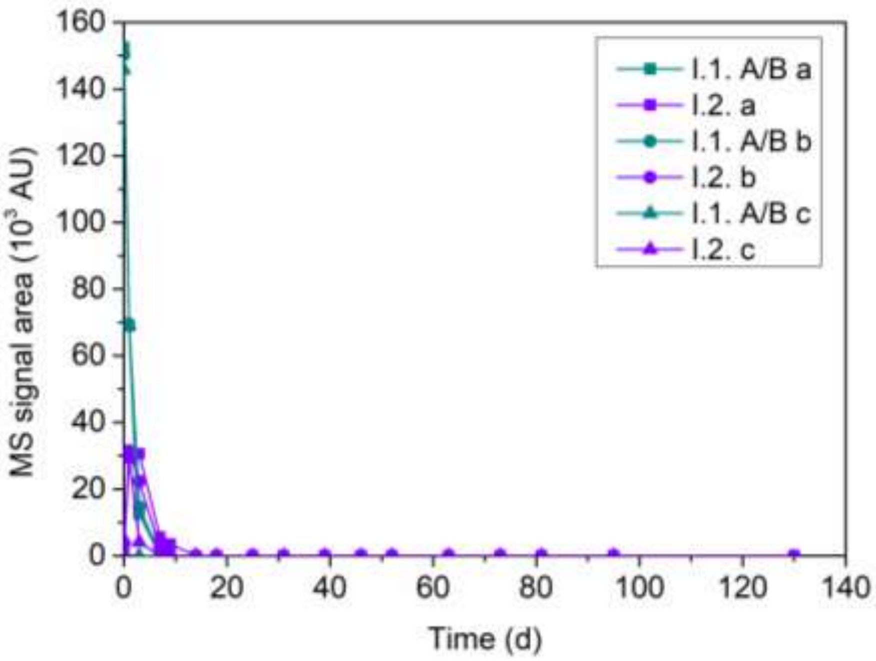

Fig. C.1.12: Concentration of the first phase TP I.1 A/B (413) and I.2 (427) of **IOP–3I** during the course of the single-compound ZWT conducted in triplicates (a, b and c). Concentration in MS signal area unit (AU).

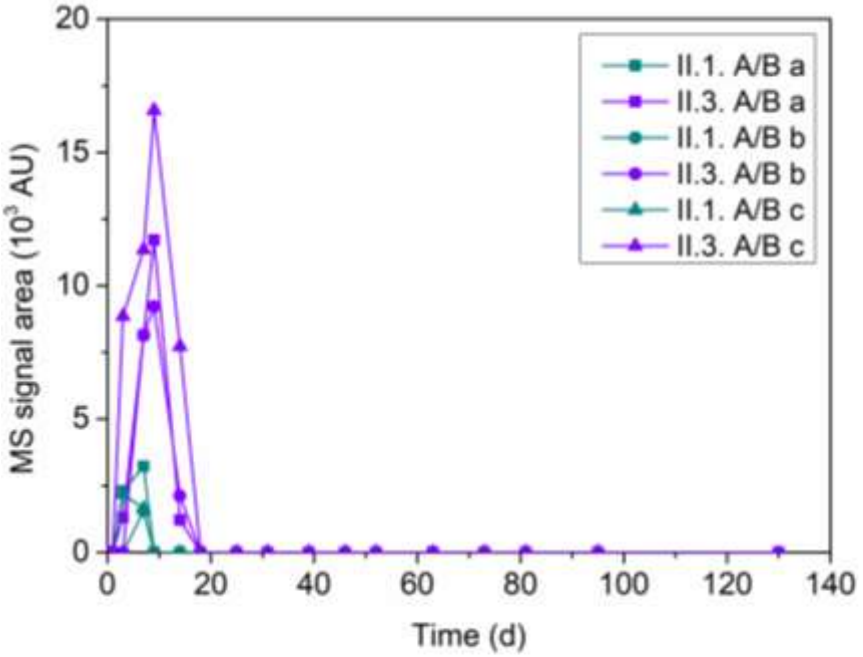

Fig. C.1.13: Concentration of the second phase TP II.1 A/B (439) and II.3 A/B (351) of **IOP–3I** during the course of the single-compound ZWT conducted in triplicates (a, b and c). Concentration in MS signal area unit (AU).

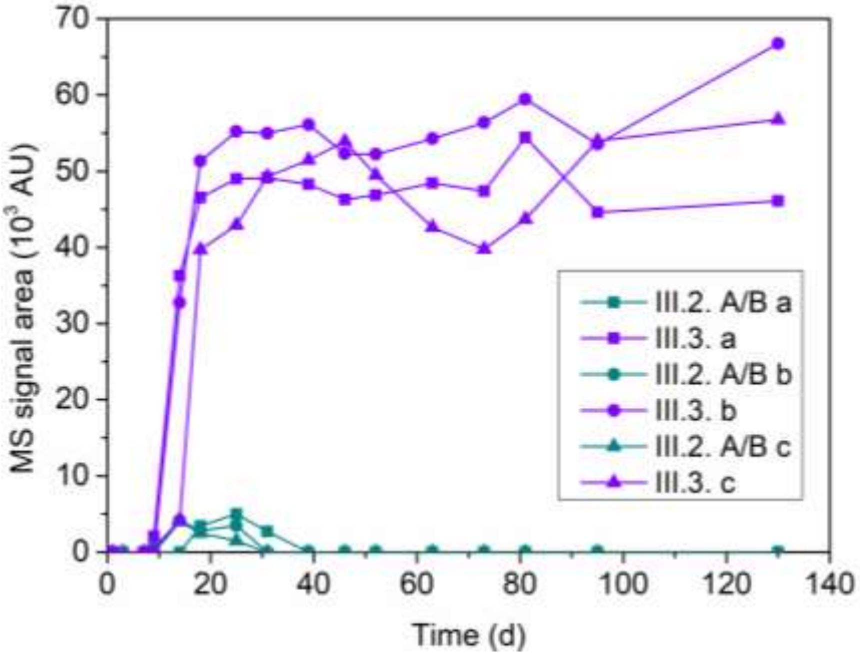

Fig. C.1.14: Concentration of the third phase TP III.2 A/B (323) and III.3 (265) of **IOP–3I** during the course of the single-compound ZWT conducted in triplicates (a, b and c). Concentration in MS signal area unit (AU).

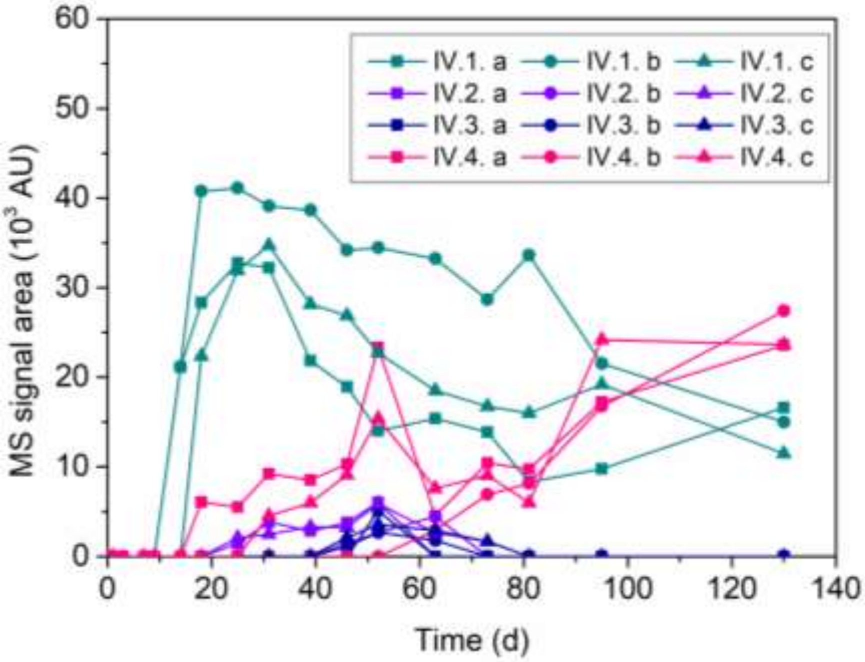

Fig. C.1.15: Concentration of the fourth phase TP IV.1 (266), IV.2 (193), IV.3 (251) and IV.4 (194) of **IOP–3I** during the course of the single-compound ZWT conducted in triplicates (a, b and c). Concentration in MS signal area unit (AU).

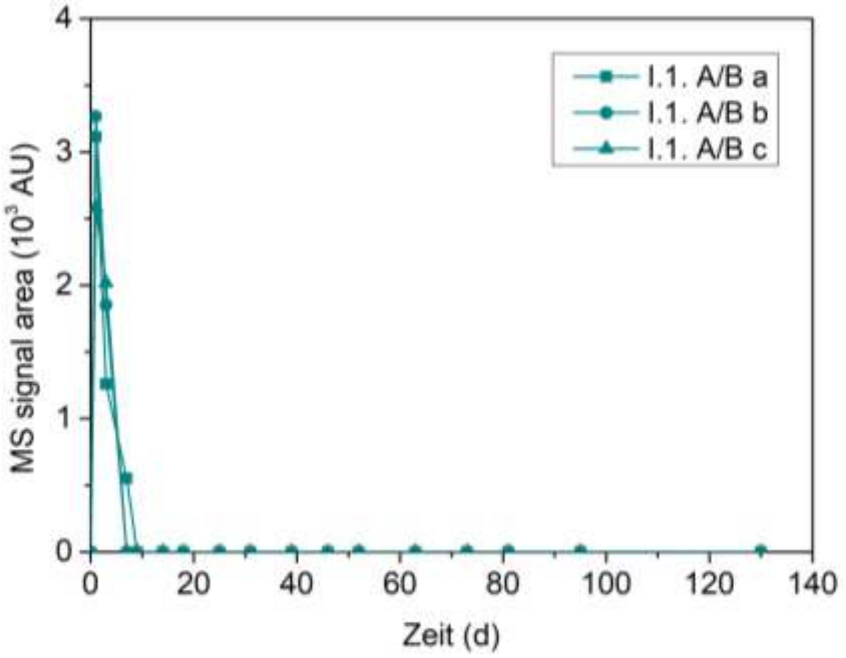

Fig. C.1.16: Concentration of the first phase TP I.1. A/B (805) of **IOP** during the course of the multi-compound ZWT conducted in triplicates (a, b and c). Concentration in MS signal area unit (AU).

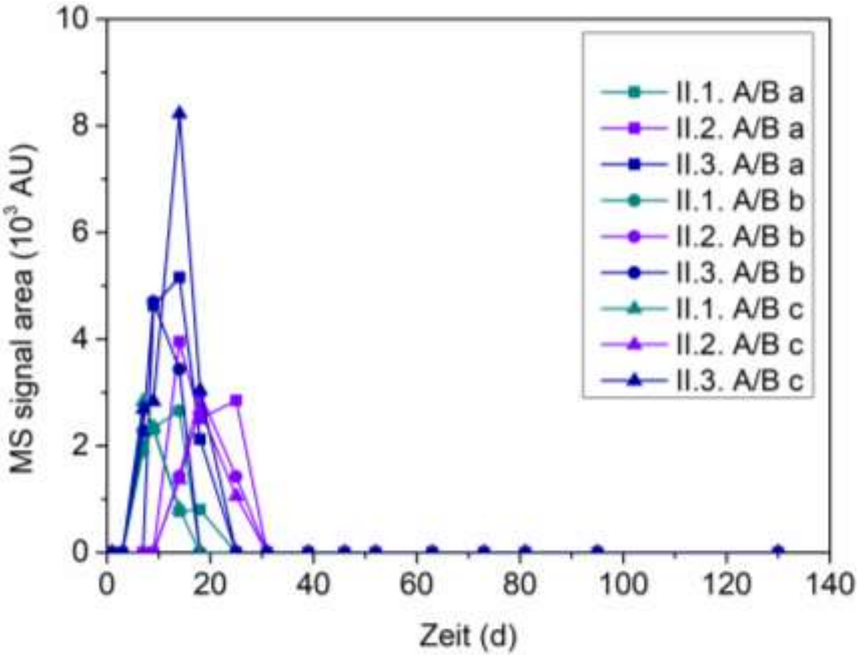

Fig. C.1.17: Concentration of the second phase TP II.1. A/B (817), TP II.2. A/B (731) and TP II.3. A/B (729) of **IOP** during the course of the multi-compound ZWT conducted in triplicates (a, b and c); concentration in MS signal area unit (AU).

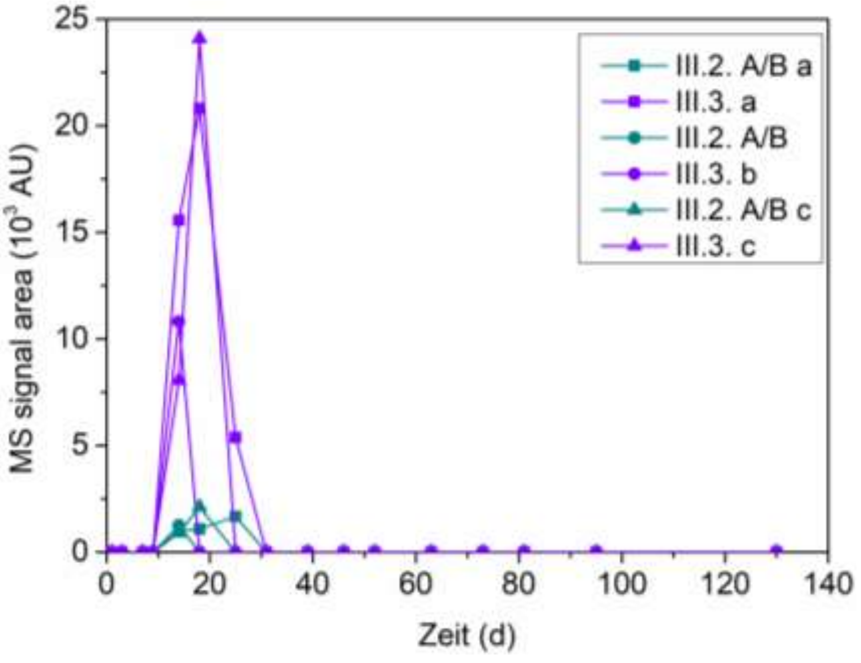

Fig. C.1.18: Concentration of the third phase TP III.2. A/B (701) and TP III.3. (643) of **IOP** during the course of the multi-compound ZWT conducted in triplicates (a, b and c). Concentration in MS signal area unit (AU).

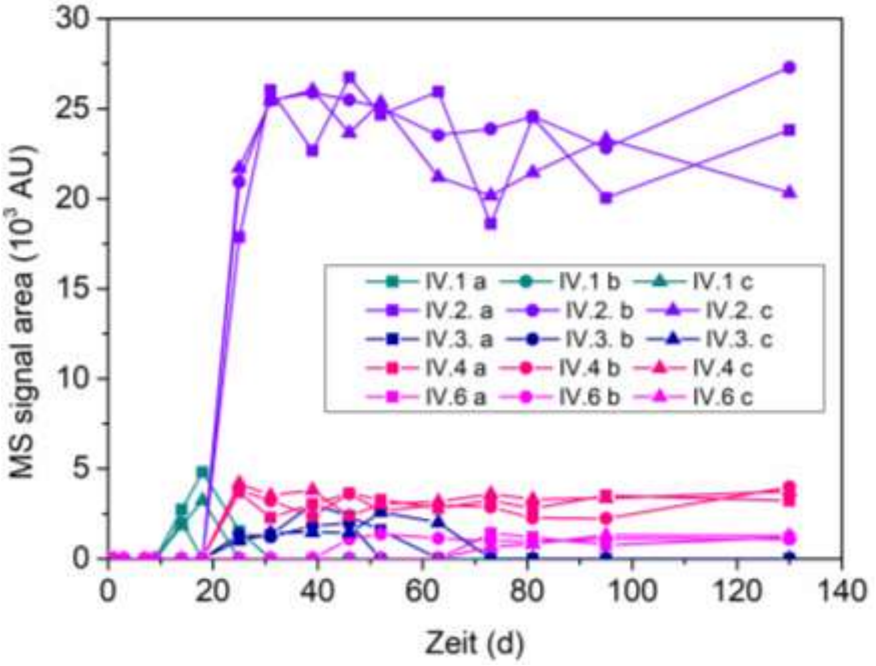

Fig. C.1.19: Concentration of the fourth phase TP IV.1 (644), IV.2 (571), IV.3 (629), IV.4 (572) and IV.6 (557) of **IOP** during the course of the multi-compound ZWT conducted in triplicates (a, b and c). Concentration in MS signal area unit (AU).

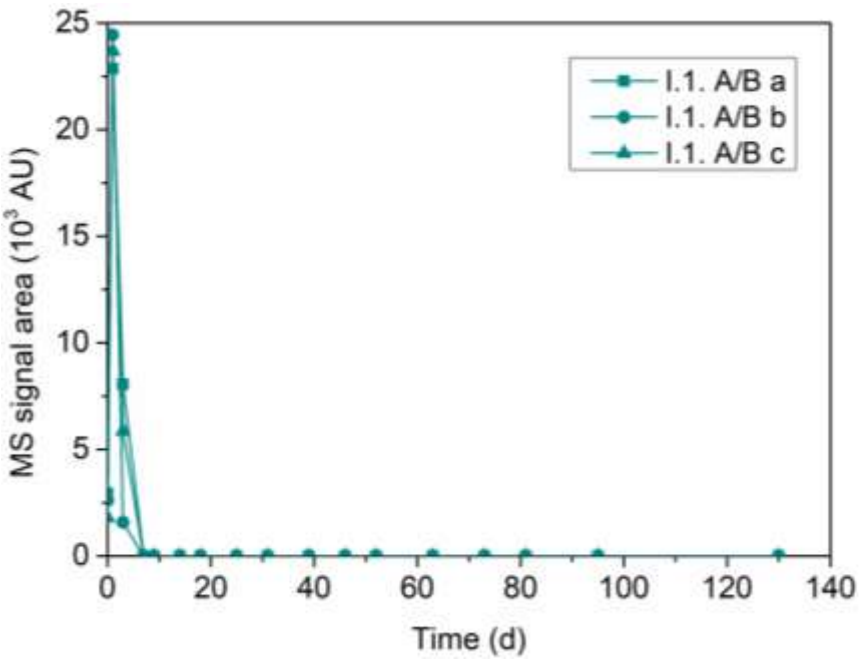

Fig. C.1.20: Concentration of the first phase TP I.1 A/B (679) of **IOP–1I** during the course of the single-compound ZWT conducted in triplicates (a, b and c). Concentration in MS signal area unit (AU).

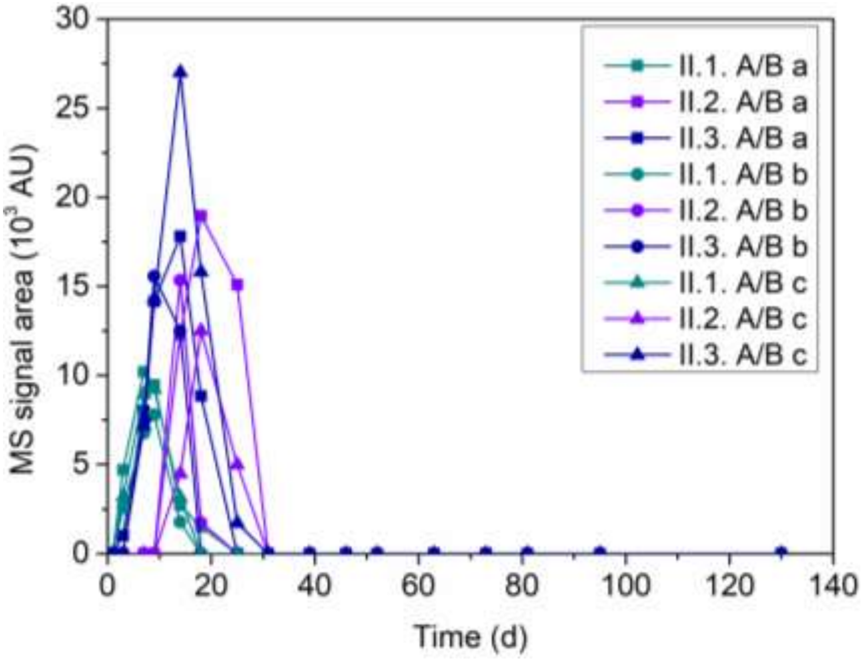

Fig. C.1.21: Concentration of the second phase TP II.1 A/B (691), II.2 A/B (605) and II.3 A/B (603) of **IOP–1I** during the course of the multi-compound ZWT conducted in triplicates (a, b and c). Concentration in MS signal area unit (AU).

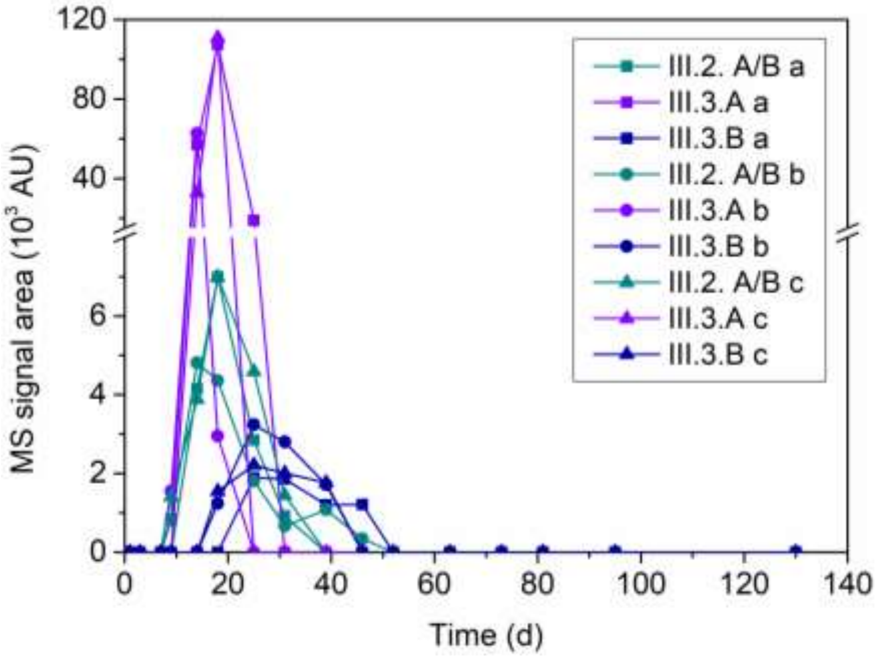

Fig. C.1.22: Concentration of the third phase TP III.2 A/B (575), III.3 A and B (517) of **IOP–1I** during the course of the multi-compound ZWT conducted in triplicates (a, b and c). Concentration in MS signal area unit (AU).

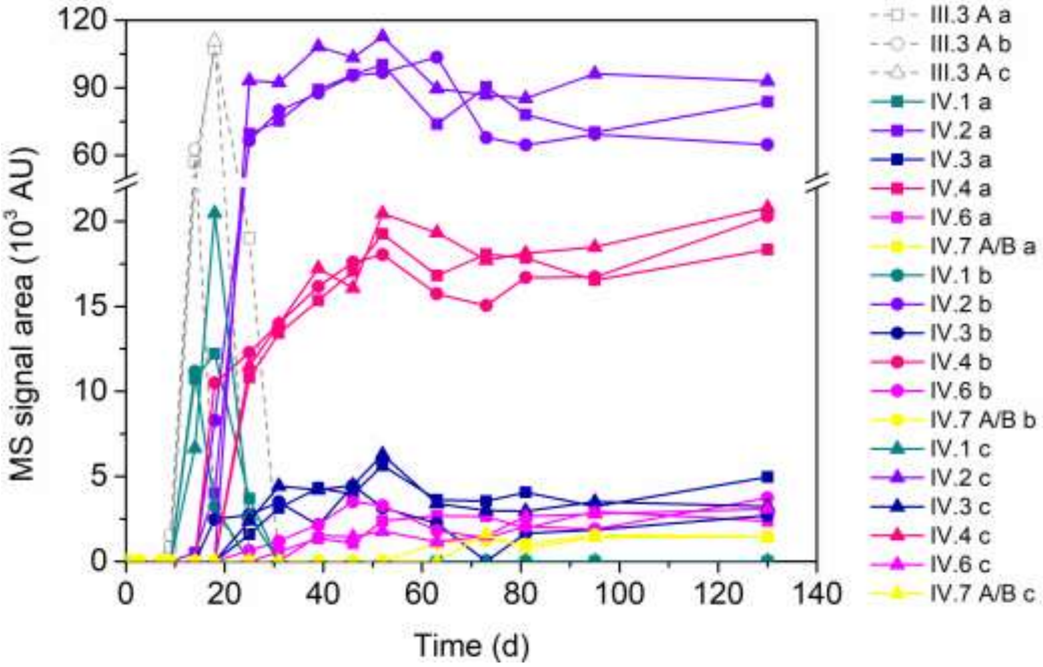

Fig. C.1.23: Concentration of the fourth phase TP IV.1 (518), IV.2 (445), IV.3 (503), IV.4 (446), IV.6 (431) and IV.7 (432) of **IOP–1I** during the course of the multi-compound ZWT conducted in triplicates (a, b and c). Concentration in MS signal area unit (AU).

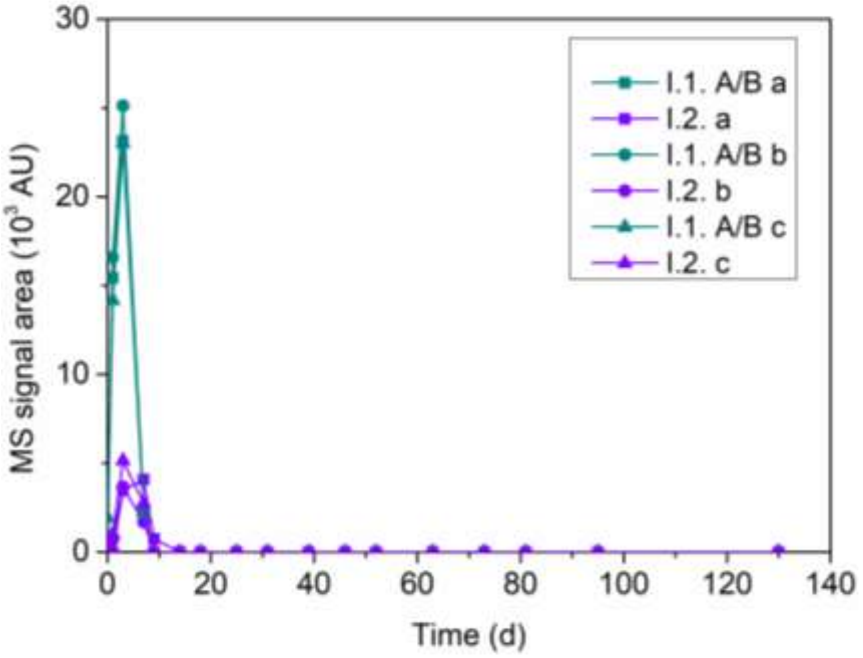

Fig. C.1.24: Concentration of the first phase TP I.1 A/B (553) and I.2 (567) of **IOP–2I** during the course of the multi-compound ZWT conducted in triplicates (a, b and c). Concentration in MS signal area unit (AU).

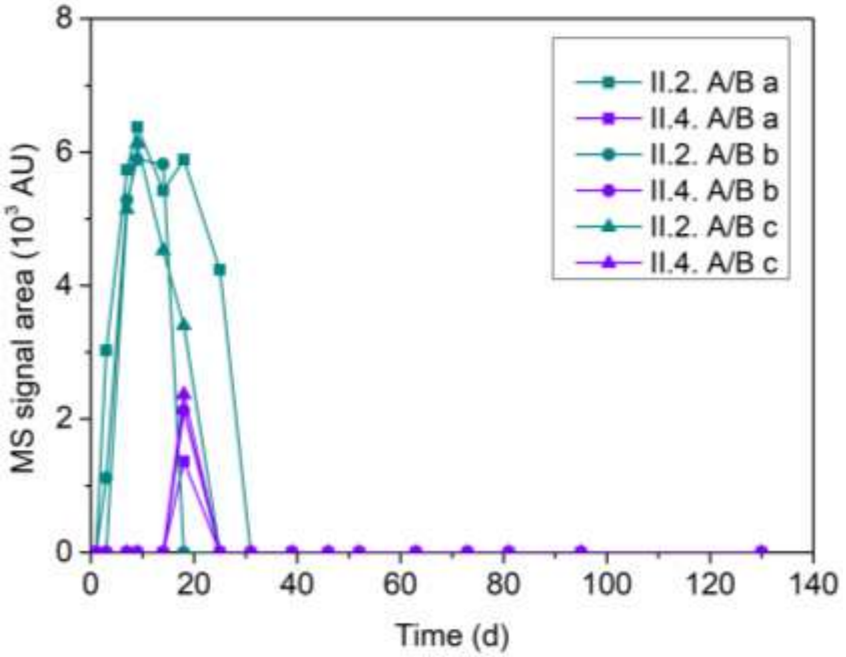

Fig. C.1.25: Concentration of the second phase TP II.2 A/B (479) and II.4 (535) of **IOP–2I** during the course of the multi-compound ZWT conducted in triplicates (a, b and c). Concentration in MS signal area unit (AU).

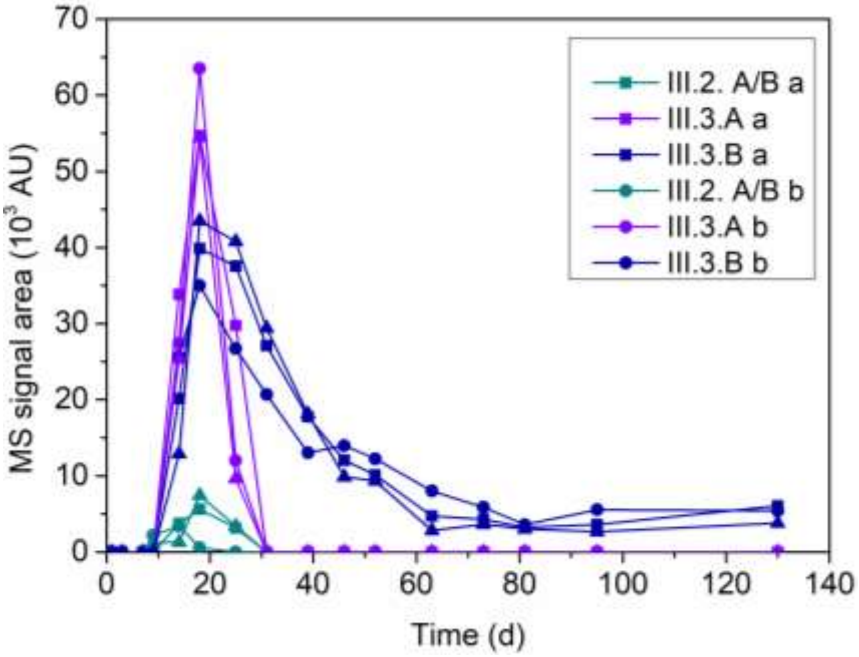

Fig. C.1.26: Concentration of the third phase TP III.2 A/B (449) and III.3 A and B (391) of **IOP–2I** during the course of the multi-compound ZWT conducted in triplicates (a, b and c). Concentration in MS signal area unit (AU).

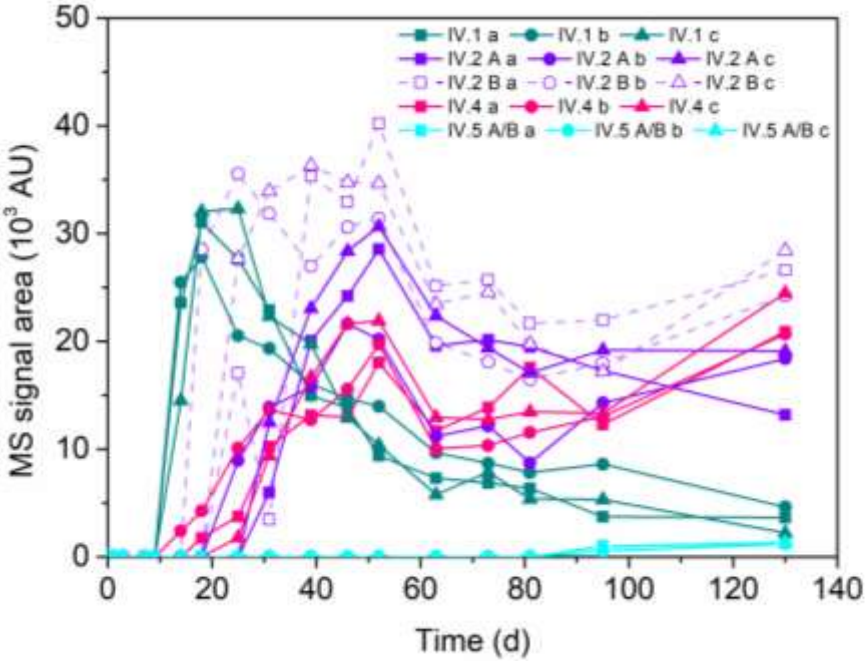

Fig. C.1.27: Concentration of the fourth phase TP IV.1 (392), IV.2 A and B (319), IV.4 (320) and IV.5 A/B (378) of **IOP–2I** during the course of the multi-compound ZWT conducted in triplicates (a, b and c). Concentration in MS signal area unit (AU).

Phase IV

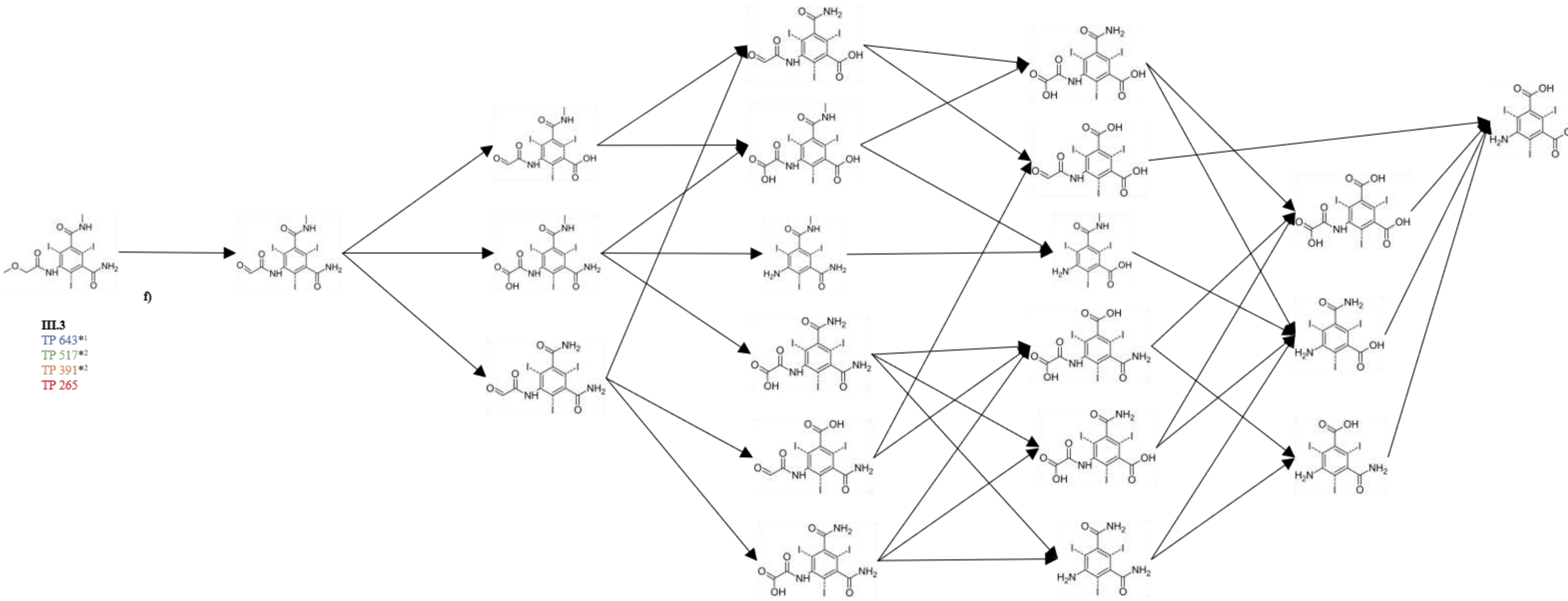

Fig. C.1.28: Demethylation of the methoxy group and oxidation of the aldehyde of chain C of DDPI derivatives.

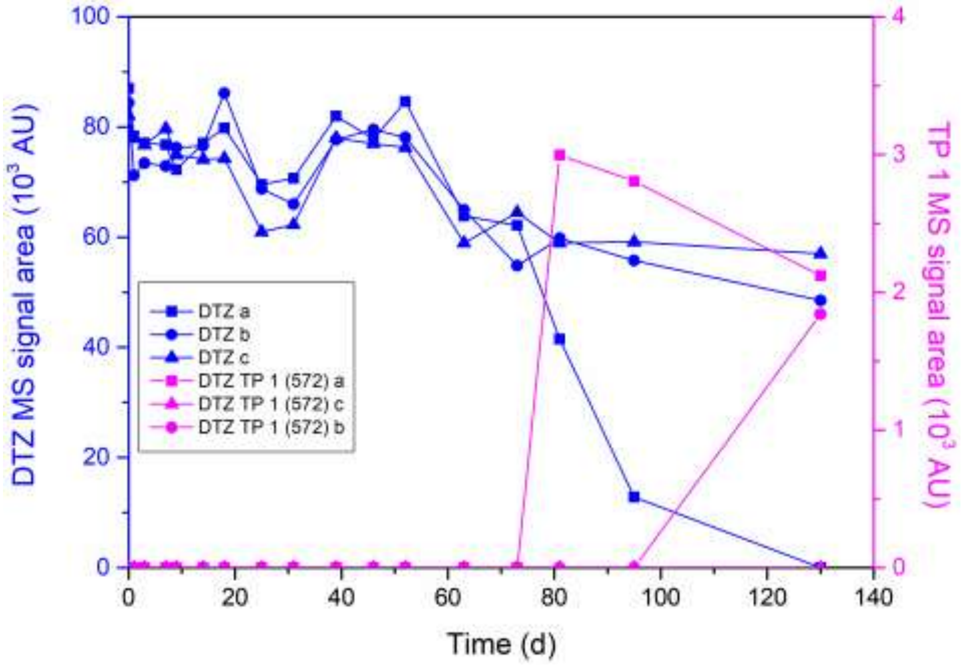

Fig. C.1.29: Concentration of **DTZ** and its TP 1 (572) during the course of the single-compound ZWT conducted in triplicates (a, b and c). Concentration is given in MS signal area unit (AU). Left axis: DTZ, right axis: TP 1.

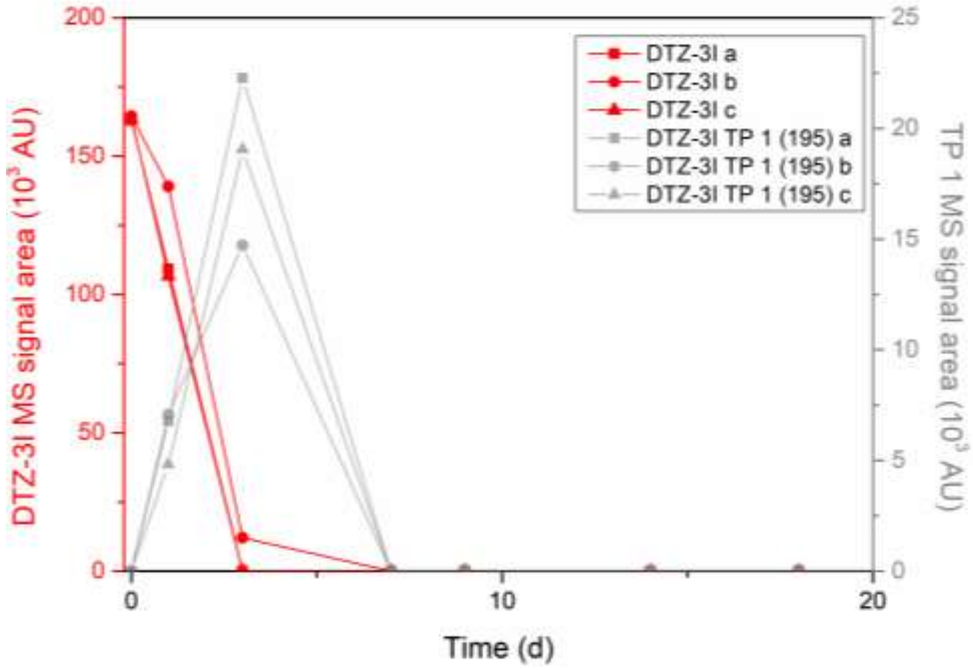

Fig. C.1.30: Concentration of **DTZ–3I** and its TP 1 (194) during the course of the single-compound ZWT conducted in triplicates (a, b and c). Concentration is given in MS signal area unit (AU). Left axis: DTZ–3I, right axis: TP 1.

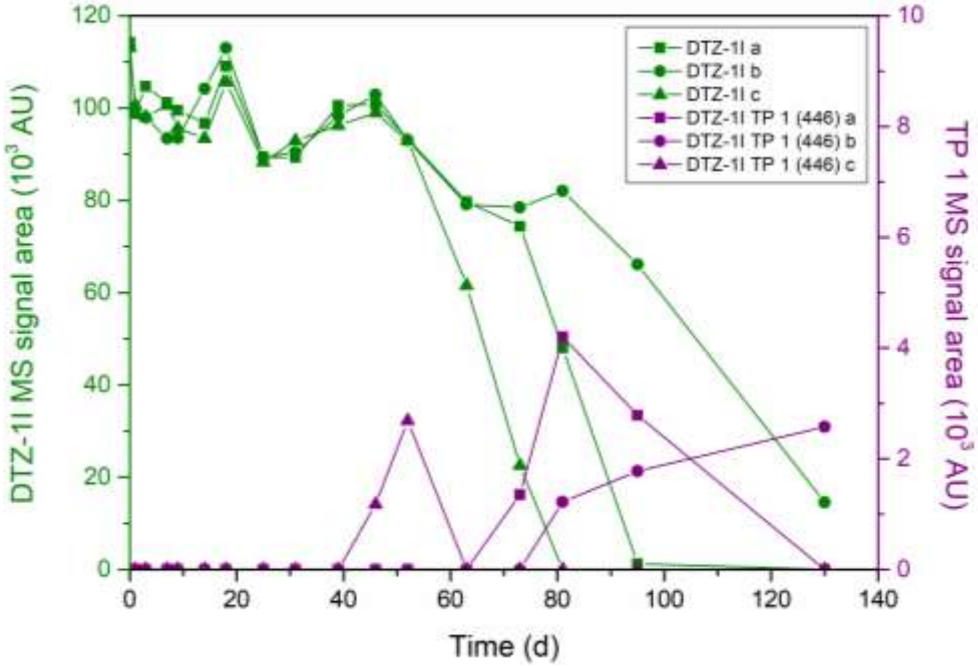

Fig. C.1.31: Concentration of **DTZ–1I** and its TP 1 (446) during the course of the multi-compound ZWT conducted in triplicates (a, b and c). Concentration is given in MS signal area units. Left axis: DTZ–1I, right axis: TP 1.

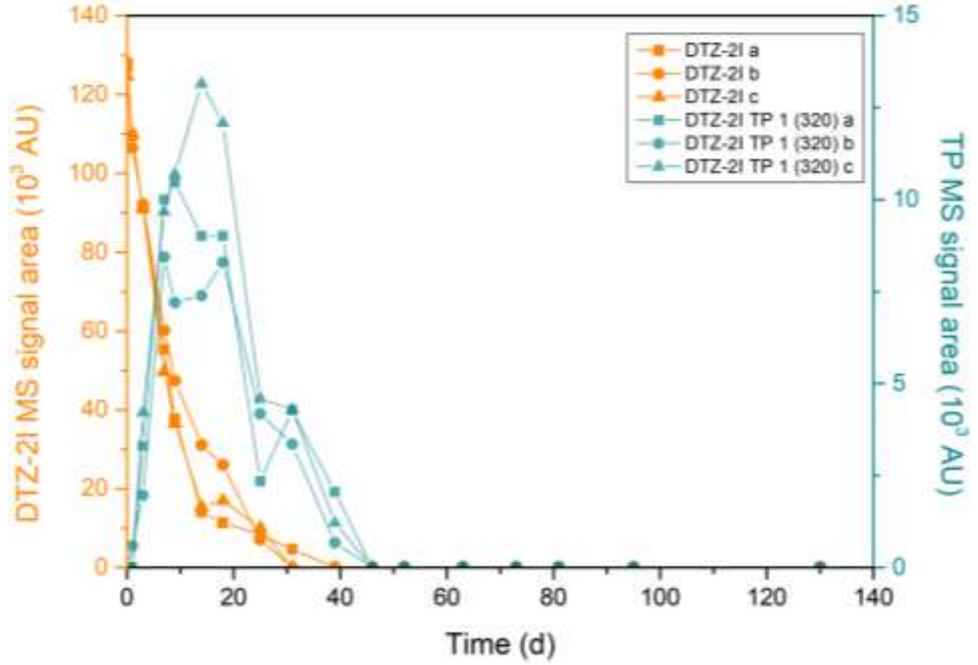

Fig. C.1.32: Concentration of **DTZ–2I** and its TP 1 (320) during the course of the multi-compound ZWT conducted in triplicates (a, b and c). Concentration in MS signal area unit (AU). Left axis: DTZ–2I, right axis: TP 1.

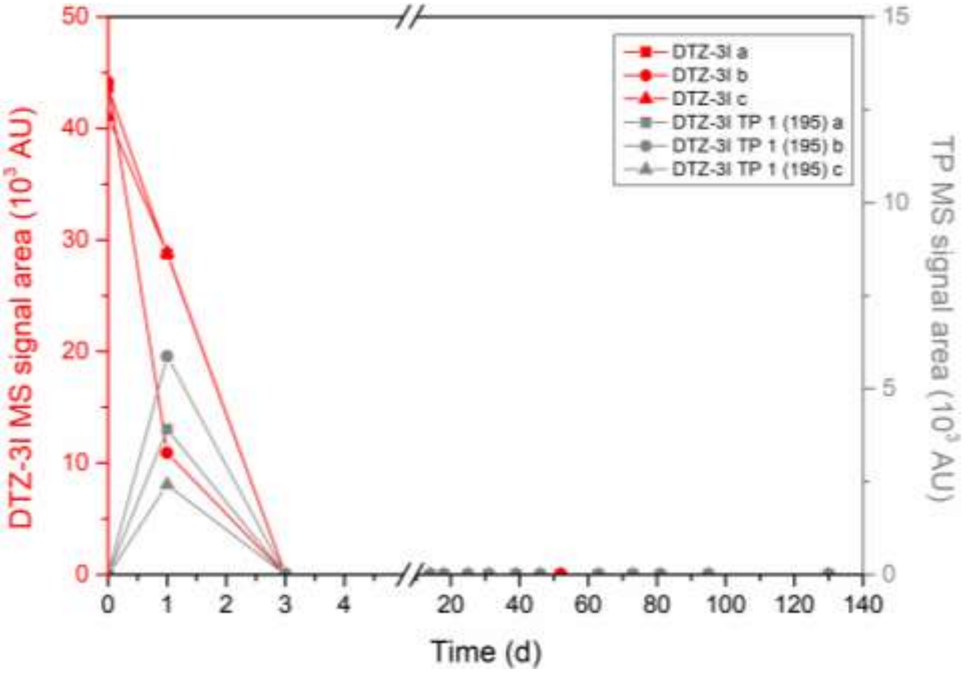

Fig. C.1.33: Concentration of **DTZ–3I** and its TP 1 (194) during the course of the multi-compound ZWT conducted in triplicates (a, b and c). Concentration in MS signal area unit (AU); Left axis: DTZ–3I, right axis: TP 1.

Table C.1.2: Hypothetical TPs of DTZ–2I and DTZ–3I following TP 2.

Initial compound	Molar mass	Structure
DTZ–3I	169	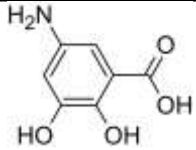
DTZ–3I	170	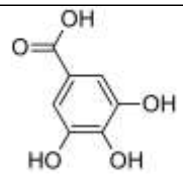
DTZ–3I	147	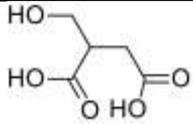
DTZ–3I	176	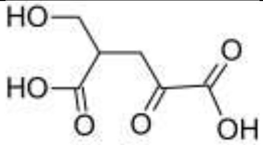
DTZ–3I	202	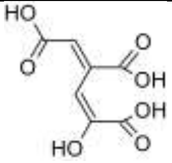
DTZ–3I	158	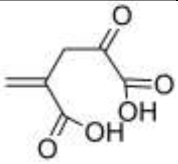
DTZ–3I	199	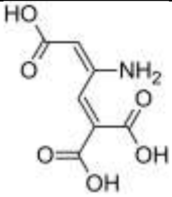
DTZ–2I	296	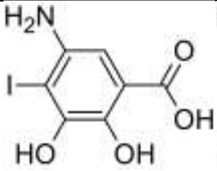 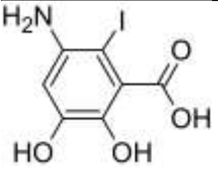
DTZ–2I	297	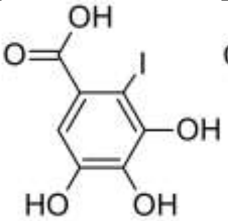 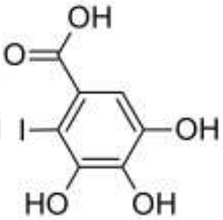
DTZ–2I	274	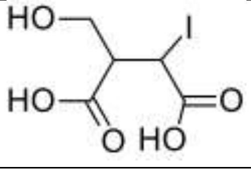

DTZ–2I	302	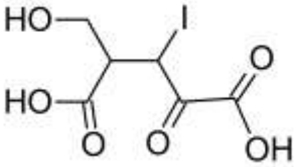
DTZ–2I	328	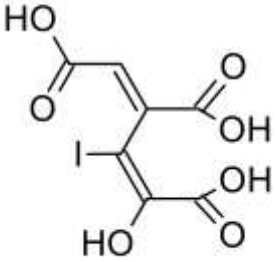
DTZ–2I	284	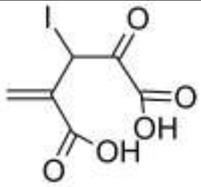
DTZ–2I	327	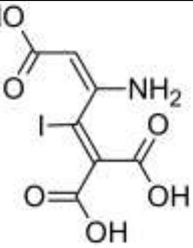

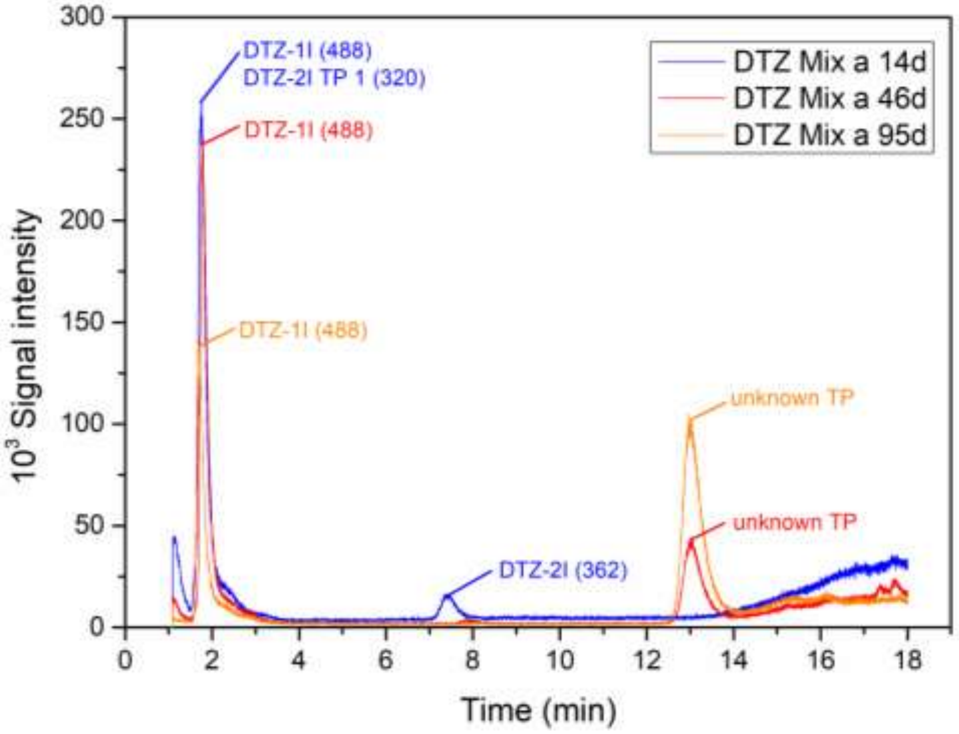

Fig. C.1.34: LC-MS chromatograms using the selective detection of organically bound iodine. The retention times of the peaks were compared with those observed in the chromatograms obtained from the scan mode. The peaks were then assigned to the corresponding masses (initial compounds and TPs).

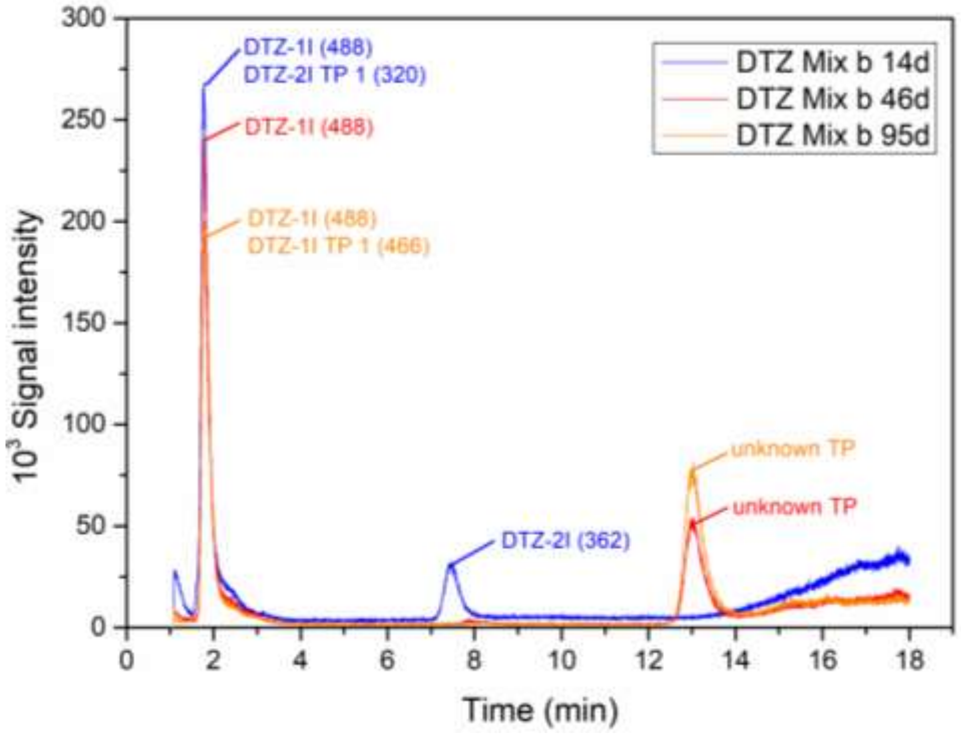

Fig. C.1.35: LC-MS chromatograms using the selective detection of organically bound iodine. The retention times of the peaks were compared with those observed in the chromatograms obtained from the scan mode. The peaks were then assigned to the corresponding masses (initial compounds and TPs).

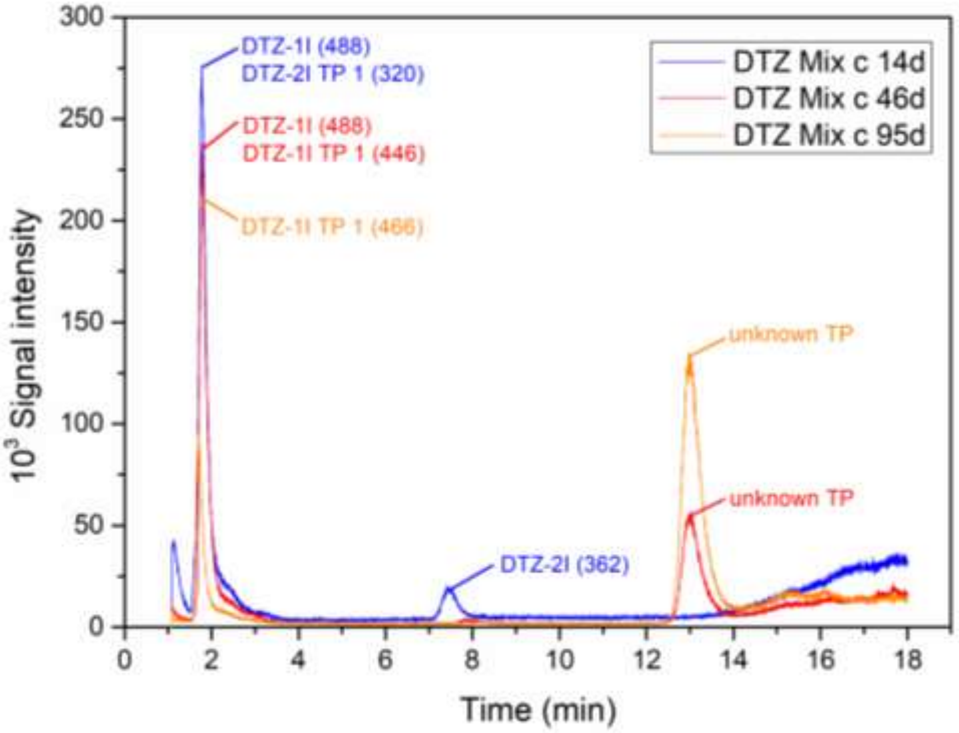

Fig. C.1.36: LC-MS chromatograms using the selective detection of organically bound iodine. The retention times of the peaks were compared with those observed in the chromatograms obtained from the scan mode. The peaks were then assigned to the corresponding masses (initial compounds and TPs).

Table C.1.3: Retention times (t_R) of all masses related to DTZ and verification of the recovery of these masses by selective ion monitoring (SIM) mode for iodide. "Yes" = matching retention times in the same sample, "overlap" = overlap of the retention time with other masses.

	**DTZ (single-compound test)**			**DTZ–1I (multi-compound test)**			**DTZ–2I (multi-compound test)**			**DTZ–3I (single- and multi-compound test)**		
	**Molar mass (g/mol)**	**t_R (min)**	**Verified in SIM for iodide**	**Molar mass (g/mol)**	**t_R (min)**	**Verified in SIM for iodide**	**Molar mass (g/mol)**	**t_R (min)**	**Verified in SIM for iodide**	**Molar mass (g/mol)**	**t_R (min)**	**Verified in SIM for iodide**
Init. Comp.	614	2.0	No measurement	488	2.2	Yes	362	7.7	Yes	236	15.3	DTZ–3I contains no iodine
TP 1	572	2.8		446	2.0	Overlap	320	1.8	Overlap	195	2.8	

C.2 Nitrate-reducing aquifer material-water tests

Table C.2.1: Difference in concentration (mg/L) or MS signal area (AU) between t_0 and t_1 (48 h) to assess initial sorption to the aquifer material. Sorption was considered valid if the mean deviation exceeded the calibration CV (triiodinated and deiodinated) or 10% (monoiodinated and diiodinated).

Aquifer material	Compound	Difference	Deviation from t_0	Mean deviation from t_0	Assessment	Sorption?
S1	IOP a<br>IOP b	0.08 mg/L<br>0.16 mg/L	3.5%<br>7.2%	**5.4 ± 2.6%**	All individual values show similar tendency, but deviations are lower than calib. CV at 0.5 mg/L.	No
	IOP–1I a<br>IOP–1I b	15,529 AU<br>11,422 AU	8.8%<br>6.2%	**7.5 ± 1.9%**	All individual values show similar tendency, but low deviations and missing external calibration data	Not significant
	IOP–2I a<br>IOP–2I b	17,709 AU<br>19,594 AU	6.6%<br>6.5%	**6.6 ± 0.0%**	All individual values show similar tendency, but low deviations and missing external calibration data	Not significant
	IOP–3I a<br>IOP–3I b	0.89 mg/L<br>1.33 mg/L	10.3%<br>13.2%	**11.8 ± 2.1%**	Deviation significantly higher than calib. CV at 1.3 mg/L.	Yes
S2	IOP a<br>IOP b	0.13 mg/L<br>0.12 mg/L	5.8%<br>5.3%	**5.6 ± 0.4%**	All individual values show similar tendency, but deviations are slightly lower than calib. CV at 0.5 mg/L.	No
	IOP–1I a<br>IOP–1I b	6,463 AU<br>13,782 AU	3.9%<br>7.6%	**5.7 ± 2.6%**	All individual values show similar tendency, but low deviations and missing external calibration data	Not significant
	IOP–2I a<br>IOP–2I b	20,937 AU<br>13,789 AU	7.8%<br>4.8%	**6.3± 2.1%**	All individual values show similar tendency, but low deviations and missing external calibration data	Not significant
	IOP–3I a<br>IOP–3I b	1.16 mg/L<br>0.91 mg/L	13.3%<br>10.3%	**11.8 ± 2.1%**	Deviation significantly higher than calib. CV at 1.3 mg/L.	Yes
S1	DTZ a<br>DTZ b	0.03 mg/L<br>0.21 mg/L	0.7%<br>5.5%	**3.1 ± 3.4%**	Deviations are lower than calib. CV at 0.5 mg/L.	No
	DTZ–1I a<br>DTZ–1I b	–1,051 AU<br>965 AU	–2.0%<br>2.1%	**0.1 ± 2.9%**	Individual deviations are ambiguous; negative deviation and missing external calibration data.	No
	DTZ–2I a<br>DTZ–2I b	165 AU<br>429 AU	3.6%<br>8.3%	**5.9 ± 3.3%**	All individual values show similar tendency, but low deviations and missing external calibration data	Not significant
	DTZ–3I a<br>DTZ–3I b	0.56 mg/L<br>1.00 mg/L	12.7%<br>20.0%	**16.3 ± 5.2%**	Deviation significantly higher than calib. CV at 1.3 mg/L.	Yes
S2	DTZ a<br>DTZ b	0.18 mg/L<br>–0.04 mg/L	4.7%<br>–1.0%	**1.8 ± 4.0%**	Individual deviations are ambiguous; negative deviation; deviations are lower than calib. CV at 0.5 mg/L.	No
	DTZ–1I a<br>DTZ–1I b	3,239 AU<br>2,848 AU	6.9%<br>5.4%	**6.1 ± 1.0%**	All individual values show similar tendency, but low deviations and missing external calibration data	Not significant
	DTZ–2I a<br>DTZ–2I b	555 AU<br>803 AU	12.6%<br>14.2%	**13.4± 1.2%**	All individual values show similar tendency; deviations higher than 10%,	Yes
	DTZ–3I a<br>DTZ–3I b	0.45 mg/L<br>0.66 mg/L	11.3%<br>12.3%	**11.8 ± 0.7%**	Deviations significantly higher than calib. CV at 0.5 mg/L.	Yes

Table C.2.2: DT_{50} values for IOP and DTZ as well as their diiodinated (-1I), monoiodinated (-2I), and deiodinated (-3I) derivatives using a modified Gompertz model determined in aquifer material-water tests. Empty field indicates that no (complete) transformation of the initial compound was observed within the test period.

Aquifer material	Compound	-k (1/d)	DT50 (d)	L (d)	λ (d)	c_{start} (fixed)	c_{end} (fixed)	R^2
S1	IOP a	0.09	69.2	73.1	62.5	1.00	0.00	0.96
	IOP b	0.10	44.7	48.4	38.3	1.00	0.00	0.98
	IOP–1I a	0.06	23.9	30.4	12.5	1.00	0.00	0.98
	IOP–1I b	0.04	38.2	48.1	21.1	1.00	0.00	0.93
	IOP–2I a	0.09	49.8	53.7	43.0	1.00	0.00	0.97
	IOP–2I b	0.10	74.4	78.3	67.8	1.00	0.00	0.91
	IOP–3I a	0.04	33.6	41.9	19.2	1.00	0.00	0.93
	IOP–3I b	0.04	58.0	67.2	42.2	1.00	0.00	0.83
S2	IOP a	0.17	38.7	40.9	35.1	1.00	0.00	0.99
	IOP b	0.17	41.1	43.2	37.4	1.00	0.00	1.00
	IOP–1I a	0.16	36.4	38.6	32.5	1.00	0.00	1.00
	IOP–1I b	0.17	40.1	42.3	36.2	1.00	0.00	0.99
	IOP–2I a	0.18	40.5	42.5	37.0	1.00	0.00	0.99
	IOP–2I b	0.19	42.3	44.3	38.9	1.00	0.00	1.00
	IOP–3I a	0.18	39.3	41.4	35.7	1.00	0.00	0.99
	IOP–3I b	0.18	42.3	44.3	38.8	1.00	0.00	1.00
S1	DTZ a	-	-	-		-	-	-
	DTZ b	-	-	-		-	-	-
	DTZ–1I a	-	-	-		-	-	-
	DTZ–1I b	-	-	-		-	-	-
	DTZ–2I a	0.31	6.37	7.56	4.30	1.00	0.00	1.00
	DTZ–2I b	0.29	5.60	6.21	2.73	1.00	0.00	0.97
	DTZ–3I a	0.54	7.75	8.44	6.57	1.00	0.00	1.00
	DTZ–3I b	0.37	7.49	8.48	5.77	1.00	0.00	0.98
S2	DTZ a	-	-	-		-	-	-
	DTZ b	-	-	-		-	-	-
	DTZ–1I a	-	-	-		-	-	-
	DTZ–1I b	-	-	-		-	-	-
	DTZ–2I a	0.29	7.74	9.00	5.56	1.00	0.00	0.98
	DTZ–2I b	0.32	8.12	9.26	6.15	1.00	0.00	1.00
	DTZ–3I a	0.66	8.24	8.80	7.28	1.00	0.00	1.00
	DTZ–3I b	0.69	7.53	8.07	6.61	1.00	0.00	0.99

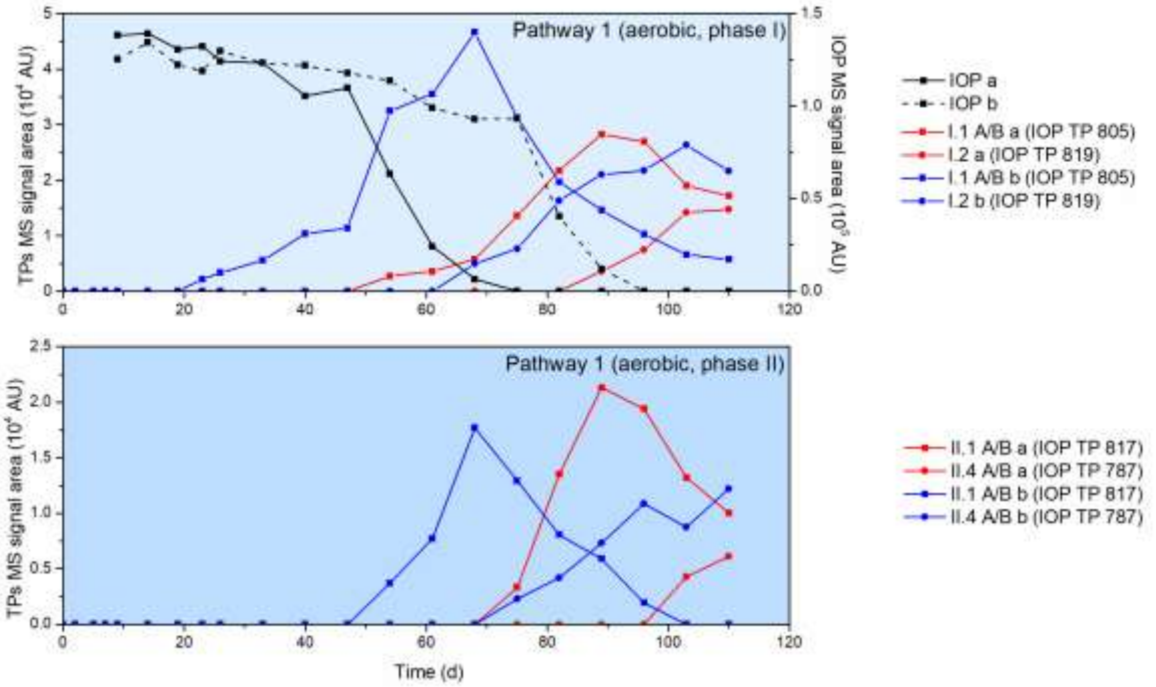

Fig. C.2.1: Concentration of TPs following the aerobic transformation pathway (pathway 1) of **IOP** occurring during the course of the aquifer material-water tests using material **S1** conducted in duplicates (a and b). Concentration is given in MS signal area unit (AU).

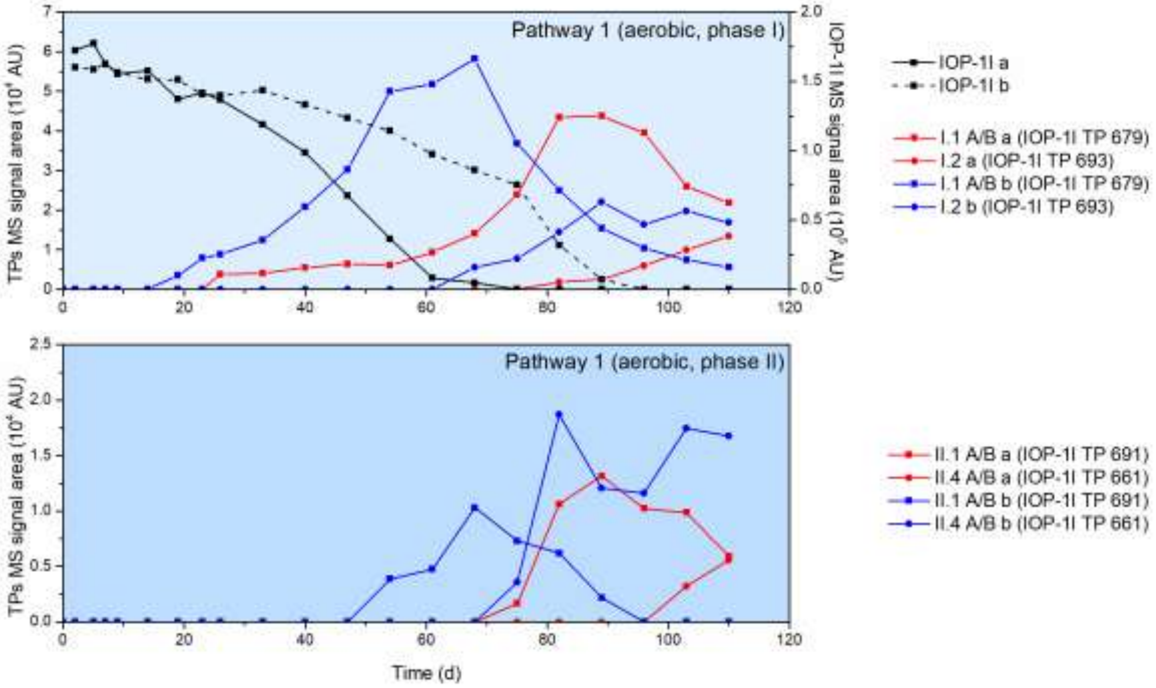

Fig. C.2.2: Concentration of TPs following the aerobic transformation pathway (pathway 1) of **IOP–1I** occurring during the course of the aquifer material-water tests using material **S1** conducted in duplicates (a and b). Concentration is given in MS signal area unit (AU).

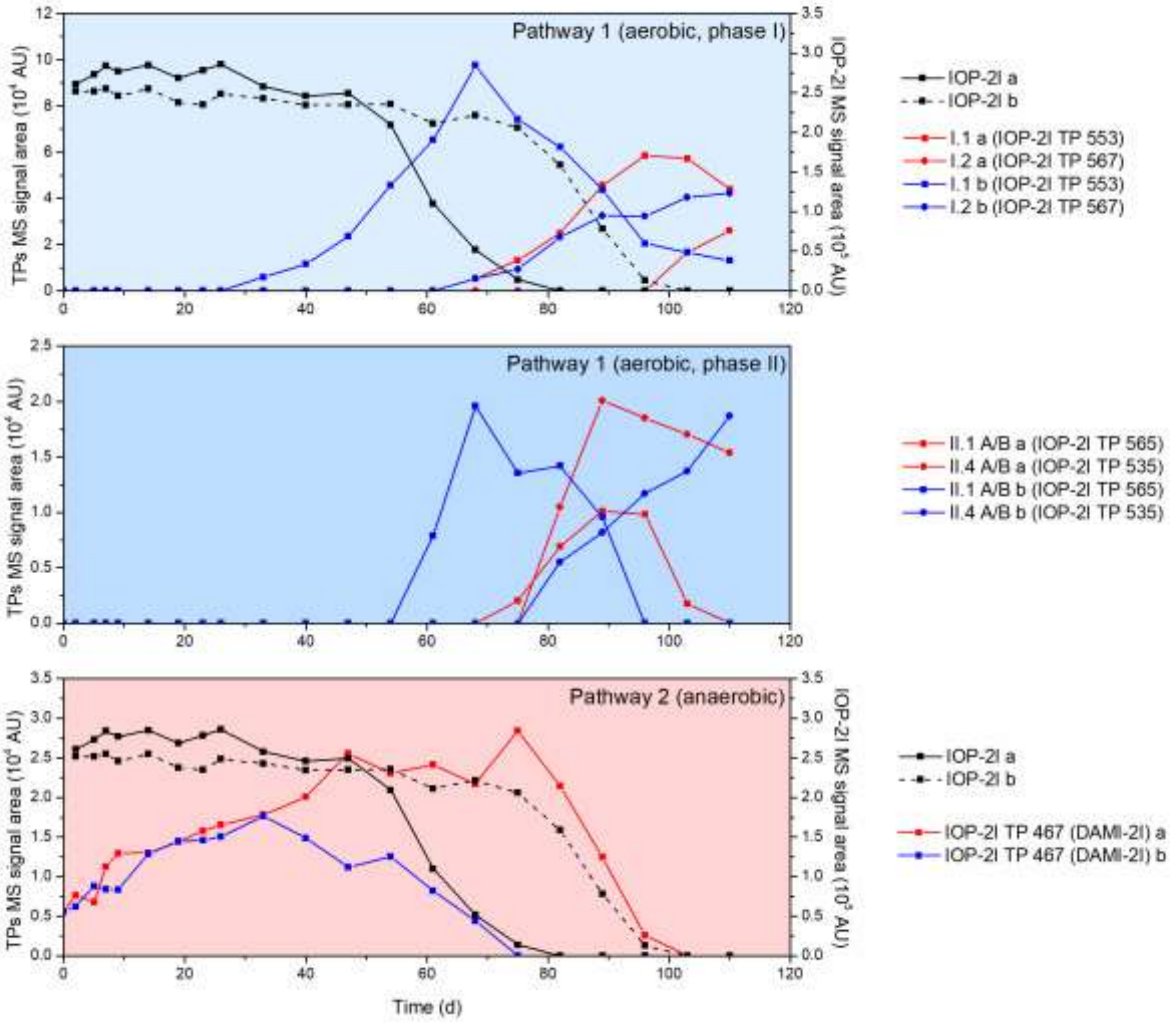

Fig. C.2.3: Concentration of TPs following the aerobic transformation pathway (pathway 1) and pathway 2 of **IOP–2I** occurring during the course of the aquifer material-water tests using material **S1** conducted in duplicates (a and b). Concentration is given in MS signal area unit (AU).

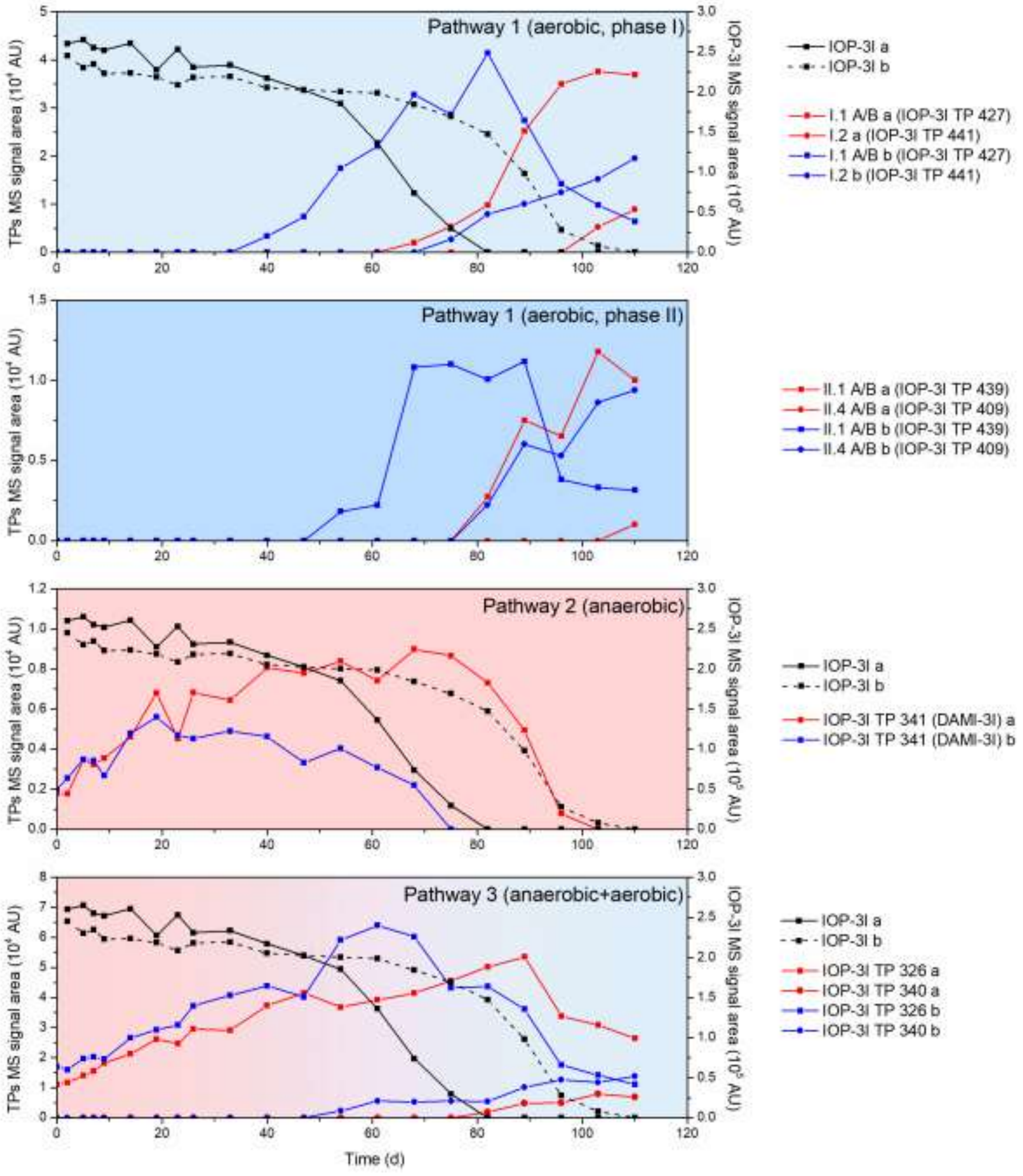

Fig. C.2.4: Concentration of TPs following the aerobic transformation pathway (pathway 1), pathway 2 and pathway 3 of **IOP–3I** occurring during the course of the aquifer material-water tests using material **S1** conducted in duplicates (a and b). Concentration is given in MS signal area unit (AU).

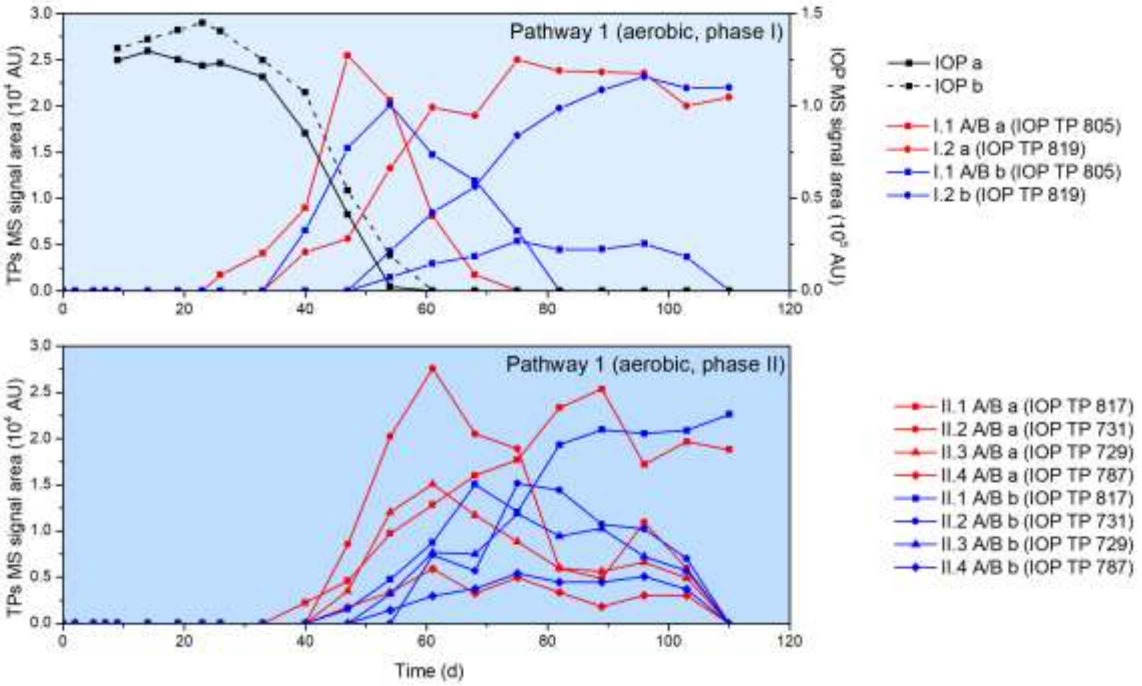

Fig. C.2.5: Concentration of TPs following the aerobic transformation pathway (pathway 1) of **IOP** occurring during the course of the aquifer material-water tests using material **S2** conducted in duplicates (a and b). Concentration is given in MS signal area units.

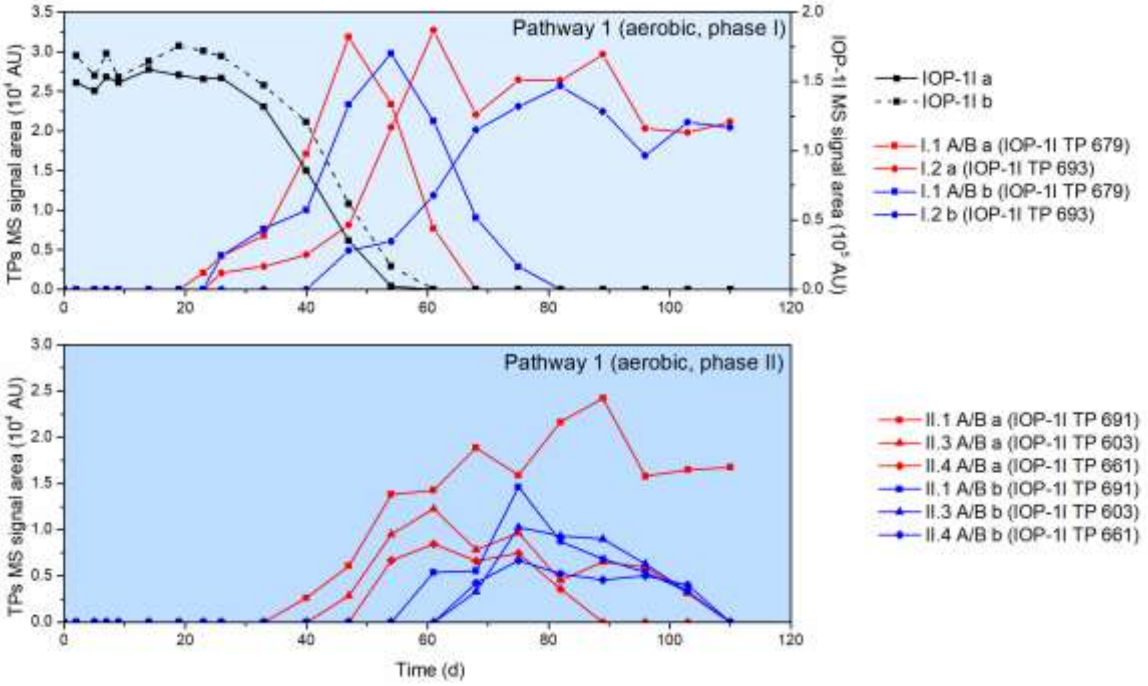

Fig. C.2.6: Concentration of TPs following the aerobic transformation pathway (pathway 1) of **IOP–1I** occurring during the course of the aquifer material-water tests using material **S2** conducted in duplicates (a and b). Concentration is given in MS signal area unit (AU).

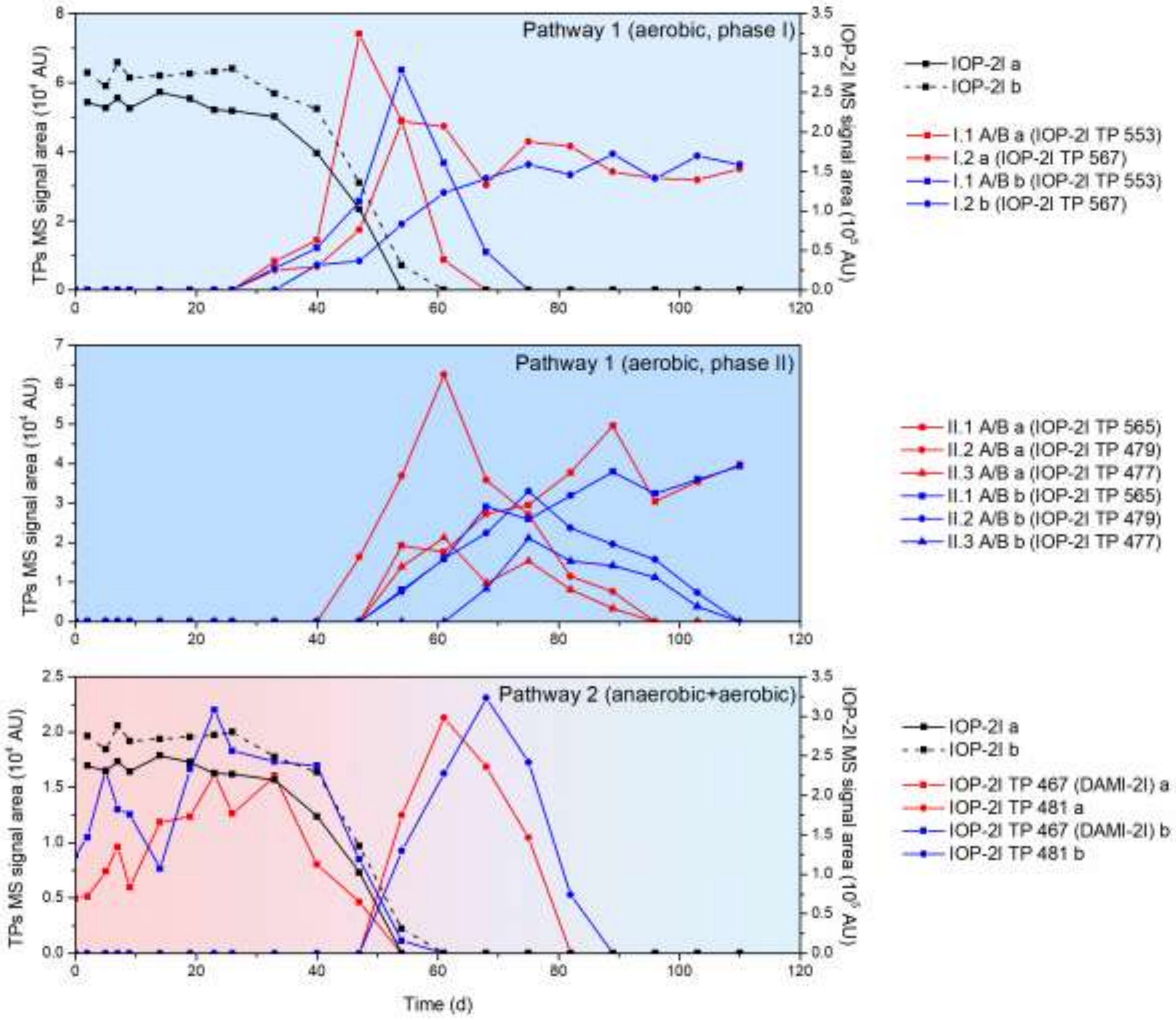

Fig. C.2.7: Concentration of TPs following the aerobic transformation pathway (pathway 1) and pathway 2 of **IOP–2I** occurring during the course of the aquifer material-water tests using material **S2** conducted in duplicates (a and b). Concentration in MS signal area unit (AU).

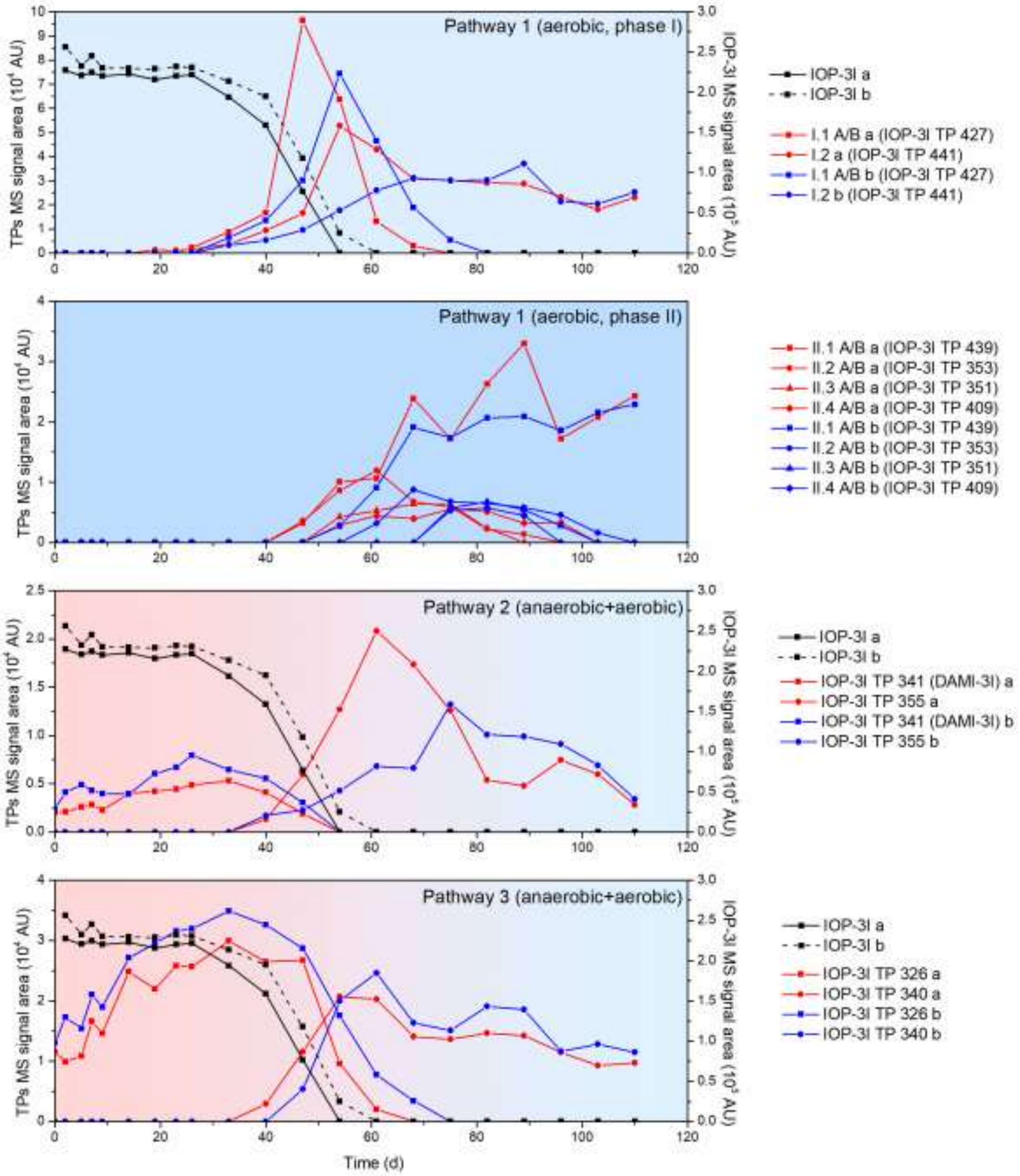

Fig. C.2.8: Concentration of TPs following the aerobic transformation pathway (pathway 1), pathway 2 and pathway 3 of **IOP–3I** occurring during the course of the aquifer material-water tests using material **S2** conducted in duplicates (a and b). Concentration is given in MS signal area unit (AU).

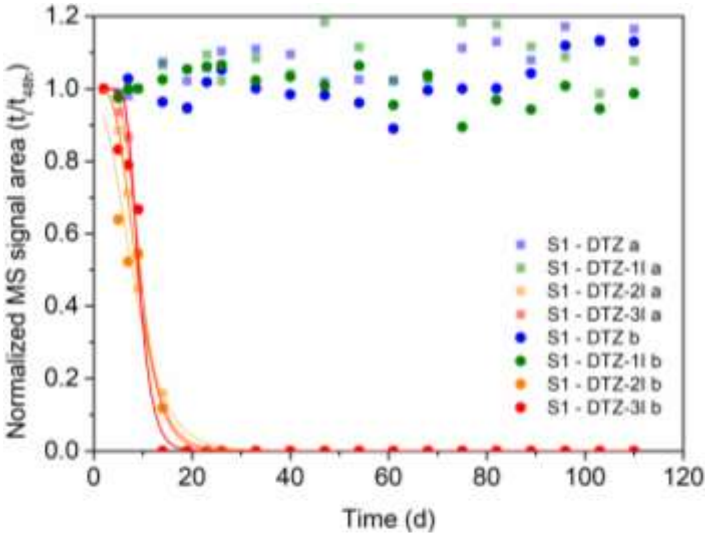

Fig. C.2.9: Fitting of the (normalized) concentration decrease of **DTZ, DTZ–1I, DTZ–2I and DTZ–3I** during the course of the multi-compound aquifer material-water tests using material **S1** conducted in duplicates (a and b). A modified Gompertz model was used for fitting.

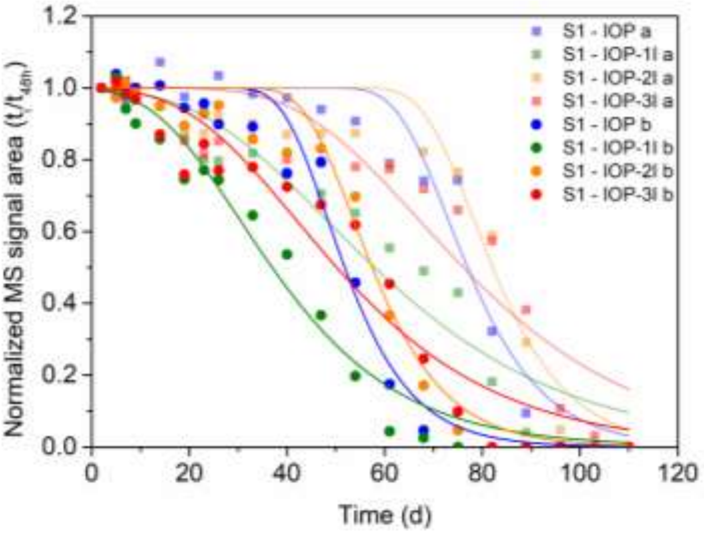

Fig. C.2.10: Fitting of the (normalized) concentration decrease of **IOP, IOP–1I, IOP–2I and IOP–3I** during the course of the multi-compound aquifer material-water tests using material **S1** conducted in duplicates (a and b). A modified Gompertz model was used for fitting.

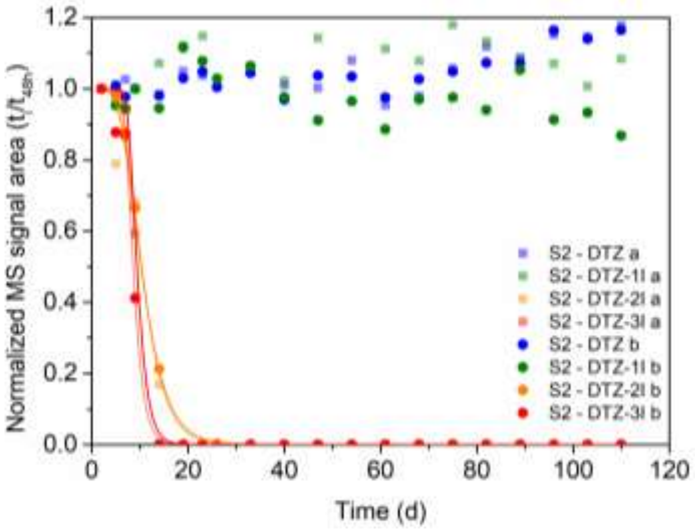

Fig. C.2.11: Fitting of the (normalized) concentration decrease of **DTZ, DTZ–1I, DTZ–2I and DTZ–3I** during the course of the multi-compound aquifer material-water tests using material **S2** conducted in duplicates (a and b). A modified Gompertz model was used for fitting.

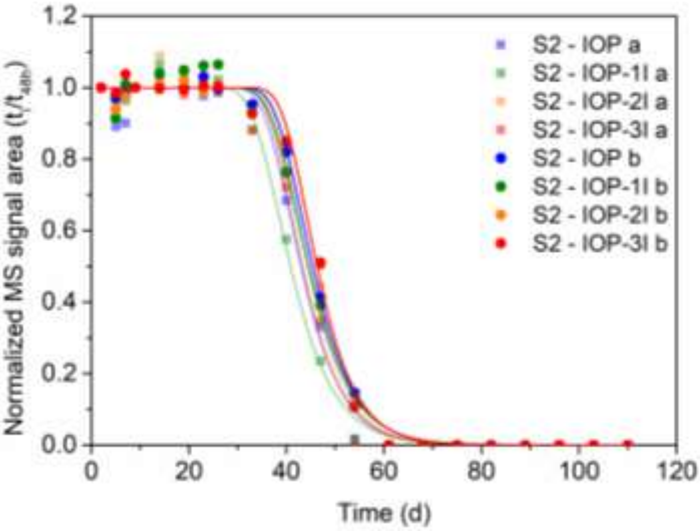

Fig. C.2.12: Fitting of the (normalized) concentration decrease of **IOP, IOP–1I, IOP–2I and IOP–3I** during the course of the multi-compound aquifer material-water tests using material **S2** conducted in duplicates (a and b). A modified Gompertz model was used for fitting.

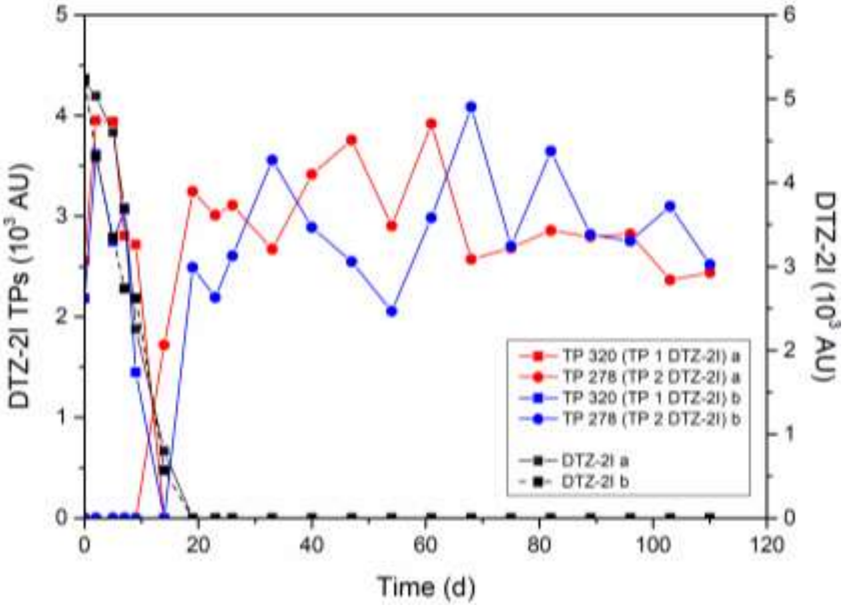

Fig. C.2.13: Concentration of TP 320 (TP 1 of DTZ–2I), TP 278 (TP 2 of DTZ–2I) and DTZ–2I during the course of the aquifer material-water tests using material **S1** conducted in duplicates (a and b). Concentration is given in MS signal area unit (AU). Left axis: TPs, right axis: DTZ–2I.

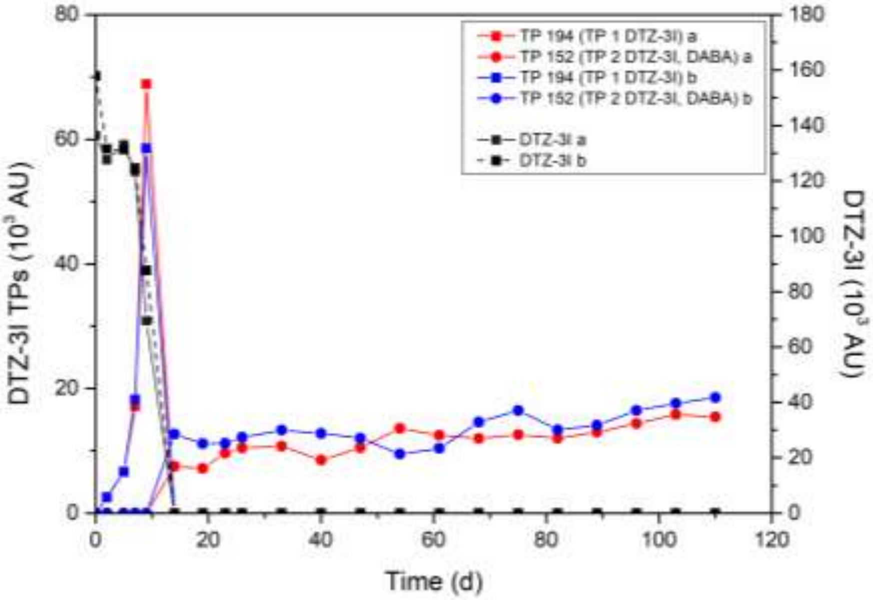

Fig. C.2.14: Concentration of TP 194 (TP 1 of DTZ–3I), TP 152 (TP 2 of DTZ–3I) and DTZ–3I during the course of the aquifer material-water test using material **S1** conducted in duplicates (a and b). Concentration is given in MS signal area unit (AU). Left axis: TPs, right axis: DTZ–3I.

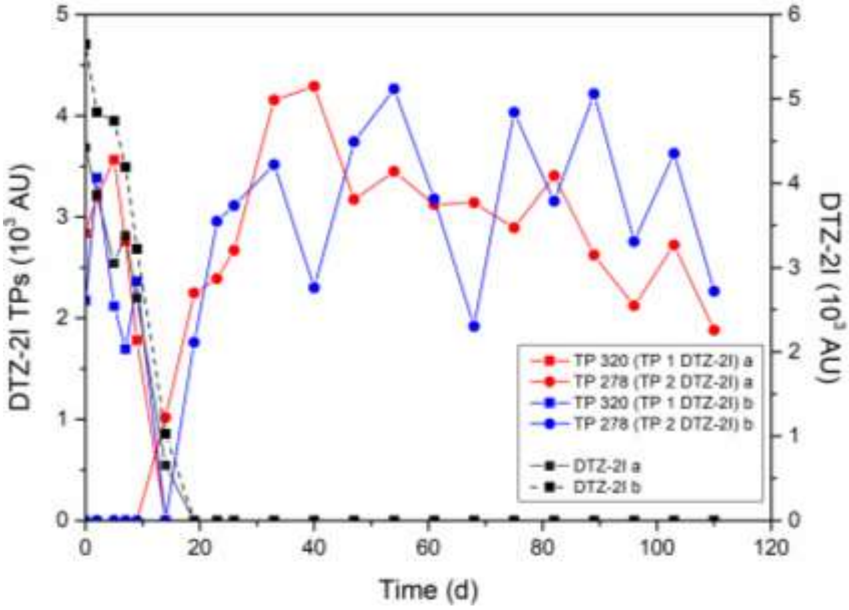

Fig. C.2.15: Concentration of TP 320 (TP 1 of DTZ–2I), TP 278 (TP 2 of DTZ–2I) and DTZ–2I during the course of the aquifer material-water test using material **S2** conducted in duplicates (a and b). Concentration is given in MS signal area unit (AU). Left axis: TPs, right axis: DTZ–2I.

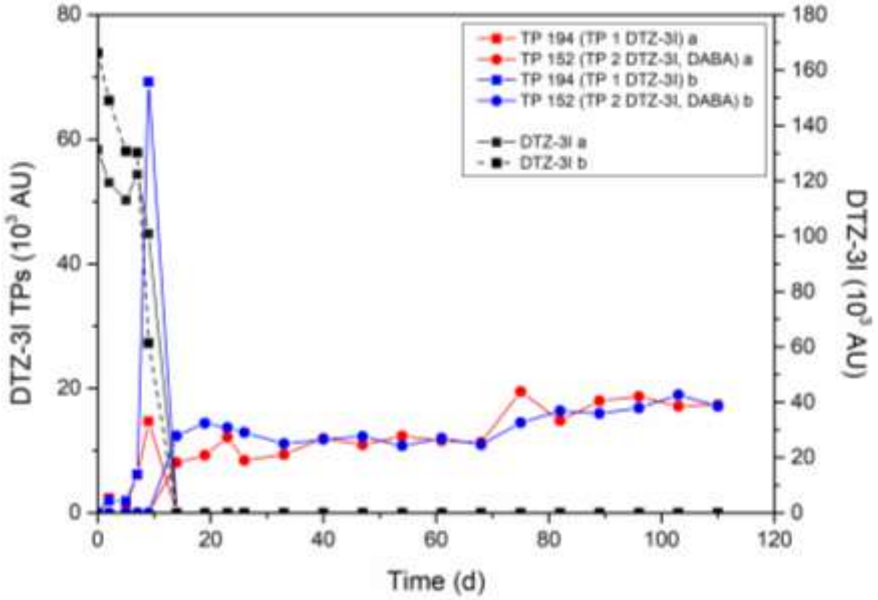

Fig. C.2.16: Concentration of TP 320 (TP 1 of DTZ–3I), TP 278 (TP 2 of DTZ–3I) and DTZ–3I during the course of the aquifer material-water test using material **S2** conducted in duplicates (a and b). Concentration is given in MS signal area units. Left axis: TPs, right axis: DTZ–3I.

References

Bartels Y, Jekel M, Putschew A. Can reductive deiodination improve the sorption of iodinated X-ray contrast media to aquifer material during bank filtration? Chemosphere 2023:138438.

El-Athman F, Adrian L, Jekel M, Putschew A. Abiotic reductive deiodination of iodinated organic compounds and X-ray contrast media catalyzed by free corrinoids. Chemosphere 2019a;221:212–8.

El-Athman F, Jekel M, Putschew A. Reaction kinetics of corrinoid-mediated deiodination of iodinated X-ray contrast media and other iodinated organic compounds. Chemosphere 2019b;234:971–7.

Haiss A, Kümmerer K. Biodegradability of the X-ray contrast compound diatrizoic acid, identification of aerobic degradation products and effects against sewage sludge micro-organisms. Chemosphere 2006;62(2):294–302.

Helbling DE, Hollender J, Kohler H-PE, Fenner K. Structure-based interpretation of biotransformation pathways of amide-containing compounds in sludge-seeded bioreactors. Environ. Sci. Technol. 2010;44(17):6628–35.

OECD. Test No. 302B: Inherent Biodegradability: Zahn-Wellens/ EVPA Test. Paris: OECD Publishing; 1992.

Schaper JL, Cirpaka OA, Posselt M, Lewandowski J, Meinikmann K, Putschew A et al. Spatial variability in trace organic compound reactivity during urban river infiltration into an alluvial aquifer. Manuscript submitted for publication. Water Resources Research 2025.

Schulz M, Löffler D, Wagner M, Ternes TA. Transformation of the X-ray Contrast Medium Iopromide In Soil and Biological Wastewater Treatment. Environ. Sci. Technol. 2008;42(19):7207–17.

Stieber M, Putschew A, Jekel M. Treatment of pharmaceuticals and diagnostic agents using zero-valent iron--kinetic studies and assessment of transformation products assay. Environmental science & technology 2011;45(11):4944–50.

U.S. Environmental Protection Agency. Estimation Programs Interface Suite™ for Microsoft® Windows, v 4.11. Washington, DC, USA: U.S. Environmental Protection Agency; 2012.

Wicker J, Lorsbach T, Gütlein M, Schmid E, Latino D, Kramer S et al. enviPath--The environmental contaminant biotransformation pathway resource. Nucleic Acids Research 2016;44(D1):D502-8.
